# Structural magnetic resonance imaging findings and histopathological correlations in motor neuron diseases—A systematic review and meta-analysis

**DOI:** 10.3389/fneur.2022.947347

**Published:** 2022-08-30

**Authors:** Charlotte Zejlon, Dominik Nakhostin, Sebastian Winklhofer, Athina Pangalu, Zsolt Kulcsar, Sebastian Lewandowski, Johannes Finnsson, Fredrik Piehl, Caroline Ingre, Tobias Granberg, Benjamin Victor Ineichen

**Affiliations:** ^1^Department of Neuroradiology, Karolinska University Hospital, Stockholm, Sweden; ^2^Department of Clinical Neuroscience, Karolinska Institutet, Stockholm, Sweden; ^3^Department of Neuroradiology, Clinical Neuroscience Center, University Hospital Zurich, University of Zurich, Zürich, Switzerland; ^4^Center of Neurology, Academic Specialist Center, Stockholm Health Services, Stockholm, Sweden; ^5^Department of Neurology, Karolinska University Hospital, Stockholm, Sweden

**Keywords:** amyotrophic lateral sclerosis, motor neuron disease, magnetic resonance imaging (MRI), systematic review, neuroimaging, meta-analysis

## Abstract

**Objectives:**

The lack of systematic evidence on neuroimaging findings in motor neuron diseases (MND) hampers the diagnostic utility of magnetic resonance imaging (MRI). Thus, we aimed at performing a systematic review and meta-analysis of MRI features in MND including their histopathological correlation.

**Methods:**

In a comprehensive literature search, out of 5941 unique publications, 223 records assessing brain and spinal cord MRI findings in MND were eligible for a qualitative synthesis. 21 records were included in a random effect model meta-analysis.

**Results:**

Our meta-analysis shows that both T2-hyperintensities along the corticospinal tracts (CST) and motor cortex T2^*^-hypointensitites, also called “motor band sign”, are more prevalent in ALS patients compared to controls [OR 2.21 (95%-CI: 1.40–3.49) and 10.85 (95%-CI: 3.74–31.44), respectively]. These two imaging findings correlate to focal axonal degeneration/myelin pallor or glial iron deposition on histopathology, respectively. Additionally, certain clinical MND phenotypes such as amyotrophic lateral sclerosis (ALS) seem to present with distinct CNS atrophy patterns.

**Conclusions:**

Although CST T2-hyperintensities and the “motor band sign” are non-specific imaging features, they can be leveraged for diagnostic workup of suspected MND cases, together with certain brain atrophy patterns. Collectively, this study provides high-grade evidence for the usefulness of MRI in the diagnostic workup of suspected MND cases.

**Systematic review registration:**

https://www.crd.york.ac.uk/PROSPERO/, identifier: CRD42020182682.

## Key points

- Structural MRI helps clinicians to assess patients with suspected motor neuron diseases.
- Motor band sign and CST hyperintensities are more prevalent in ALS patients.

## Introduction

Motor neuron diseases (MNDs) are a group of mostly fatal disorders, including entities such as amyotrophic lateral sclerosis (ALS) and progressive muscular atrophy (PMA) (1). Particularly ALS has initially been classified as a disease confined solely to the motor domain. However, increasing insights into its pathomechanisms by neuroimaging and pathology studies also indicate widespread involvement of non-motor central nervous system (CNS) domains (2). Thus, ALS is now accepted to constitute a continuum with frontotemporal dementia (FTD) (3) with pure ALS and pure FTD representing polar opposites of the spectrum which is also corroborated by shared genetic risk factors (4). Intermediate phenotypes along the spectrum are ALS with behavioral or cognitive impairment, ALS-FTD (ALS patients meeting the Neary criteria for FTD) and FTD-MND, i.e., FTD patients without sufficient motor neuron involvement for an ALS diagnosis. To further increase the complexity, upper and lower motor neurons can be affected to different extents in ALS: Affection of both motor neuron classes constitutes classical ALS. Lower motor neuron predominance has been classified as progressive muscular atrophy (PMA), whereas upper motor neuron predominance as primary lateral sclerosis (PLS) (3).

Based on these phenotypic variabilities, and to some degree also the overlap with other neurodegenerative or neuroinflammatory conditions, the diagnostic workup of suspected MND cases requires diligence. One key aspect is ruling out alternative diagnoses clinically mimicking ALS. For this, magnetic resonance imaging (MRI) has become an important paraclinical tools (5–7). And a growing understanding of MND from the imaging perspective has also revealed more candidate imaging biomarkers for sub-phenotypes of MND such as distinct CNS atrophy patterns. Such imaging features have been systematically reviewed for certain MND entities such as for C9orf72-carriers (8). Also, the association of such MRI features to clinical disability has been systematically assessed (9). However, there is a lack of a comprehensive and systematic overview summarizing potential neuroimaging biomarkers for MND diagnosis facilitating diagnostic workup for (neuro-)radiologists (10).

Based on these shortcomings, this systematic review had the following three objectives: (1) Summarize the available evidence on imaging biomarkers in MND on structural MRI to facilitate diagnostic workup for (neuro-)radiologists, (2) Assessment of the underlying pathological tissue signature of these imaging features; and (3) Assessment of how well these imaging features can be leveraged to discriminate clinical MND phenotypes.

## Methods

We registered the study protocol in the International prospective register of systematic reviews (PROSPERO, CRD42020182682, https://www.crd.york.ac.uk/PROSPERO/) and used the Preferred Reporting Items for Systematic Reviews and Meta-Analysis (PRISMA) Guidelines for reporting (11).

### Search strategy

We searched for original publications up to April 08, 2021, in PubMed, Web of Science and Ovid EMBASE. The search was created for Medline *via* Pubmed by information specialists at the Karolinska Institute, using the MeSH term “Motor Neuron Disease” as well as title and abstract search for motor neuron diseases. This search was adjusted for Web of Science and Ovid EMBASE. See Supplementary table 1 for the detailed search strings in each of these databases.

### Inclusion and exclusion criteria

We included original publications (including case reports) that reported on any structural MRI outcome related to MND in humans. Conference abstracts, non-English articles, and publications which reiterated previously reported quantitative data were excluded. Reviews were excluded but retained as potential sources for additional records.

### Study selection and data extraction

Titles and abstracts of studies were screened for their relevance in the web-based application Rayyan (12) by two reviewers (CZ and BVI) followed by full-text screening. From eligible full texts, the following data was extracted: title, authors, publication year, study design, type of MND, and number of subjects per group. For studies with n≥5 cases, also MRI sequences/field strength, main study findings (in narrative fashion), and proportions of focal imaging signs in disease and control groups were extracted.

### Quality assessment

Risk of bias was assessed against pre-defined criteria by two reviewers (CZ and BVI) using the Newcastle Ottawa Scale (13).

### Data synthesis and analysis

All analyses were carried out using R (version 3.6.1) (14) with the *meta* and *metafor* package (version 2.4.0) (15). For the meta-analysis, we used summary-level data only. As primary outcome, we assessed log-transformed odds ratios (OR) of focal MRI sign in MND patients vs. controls. Since absolute differences in focal MRI signs were assessed using different methods across studies, standardized mean differences (SMDs) with 95% confidence interval (CI) were reported as a measure of association for continuous outcome measures.

For each association of interest, between-study heterogeneity was assessed using the *I*^2^ statistics (16). SMDs and ORs were pooled using random effects models. A two-tailed *p*-value < 0.05 was considered statistically significant.

### Publication bias

Publication bias was assessed by using Egger's regression test and the rank correlation test.

## Results

### General study characteristics

#### Eligible publications

**Figure 1:**
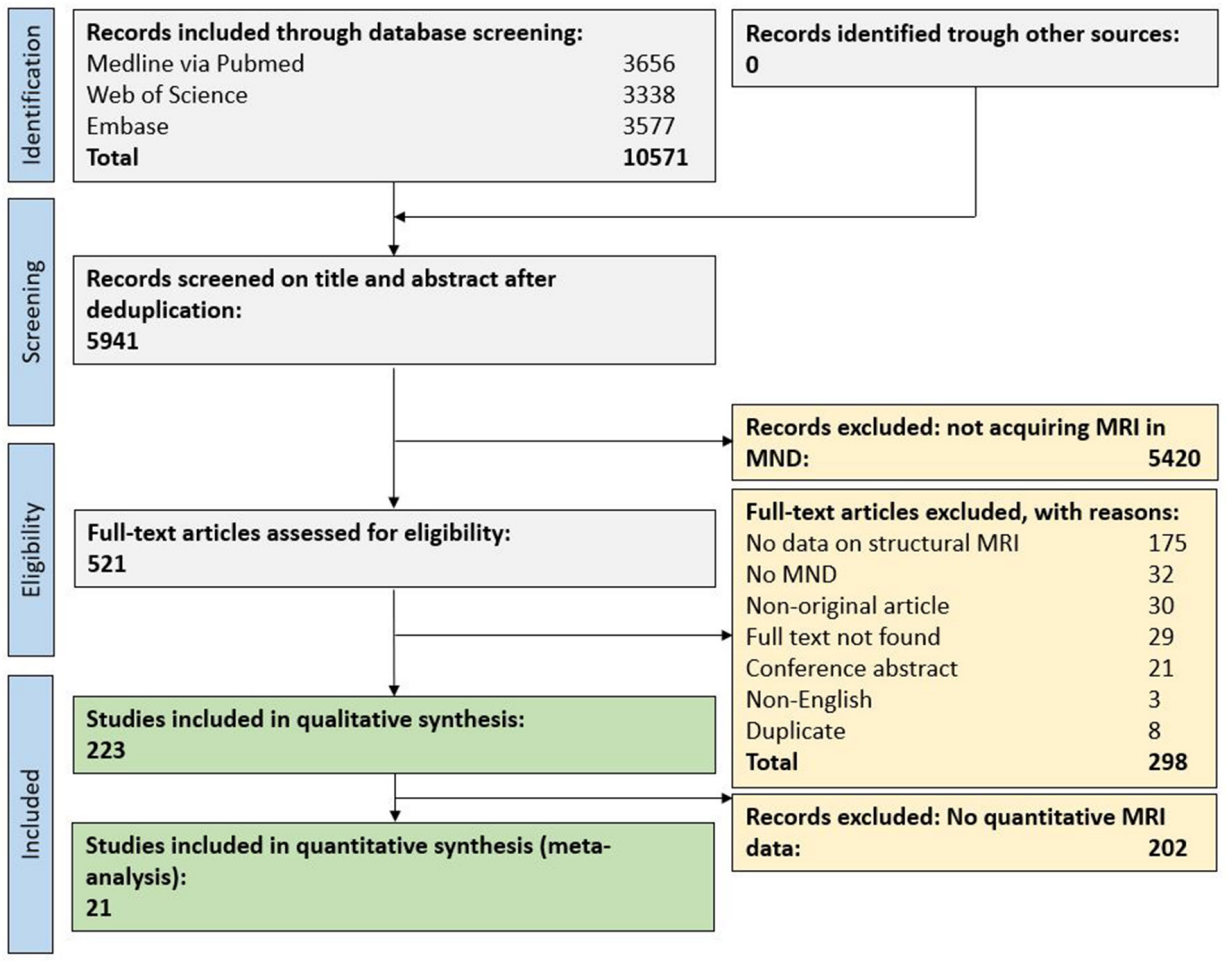
Flow chart for study inclusion. MND, motor neuron disease; MRI, magnetic resonance imaging.

In total, 10,571 original publications were retrieved from our comprehensive database search. After automated deduplication as well as abstract and title screening, 521 publications were eligible for full-text search. After screening the full text of these publications, 223 publications (4% of deduplicated references) were included for qualitative synthesis and 21 publications (0.4%) for the quantitative synthesis (Figure 1).

#### Diseases of eligible publications

Of the eligible publications, 192 investigated neuroimaging findings in ALS (*n* = 7,235 cases), 35 in ALS-FTD or FTD-MND (*n* = 1,808), 14 in PLS (*n* = 272), three in familial ALS (i.e., with mutations in SOD1), one in monomelic amyotrophy (*n* = 109), one in O'Sullivan–McLeod syndrome (*n* = 7) and one in pre-symptomatic carriers of C9orf72 (*n* = 15). These publications together included a total of 5,704 control subjects.

#### Risk of bias assessment

A majority of studies showed a low risk of bias for the selection domain, i.e., whether patients and controls were defined according to acknowledged diagnostic criteria; see Supplementary table 2. Many publications did not report on adjusting their statistical analyses for subject age, sex, or other potential confounders (comparability domain), thus potentially inducing biases.

### Magnetic resonance imaging acquisition and assessed imaging biomarkers

MRI findings are summarized in Table 1. The most frequently assessed MRI biomarker in motor neuron diseases were whole-brain or regional CNS volumes, i.e., white and/or gray matter atrophy (156 publications) or spinal cord atrophy (six publications). Another frequently assessed MRI feature was hyperintensities along the corticospinal tracts (CST) on T2-weighted (T2w) imaging (40 publications) or proton density-weighted (PDw) imaging (10 publications). Less commonly assessed imaging biomarkers were signal loss in the motor cortex on susceptibility-weighted (SWI) or T2^*^-weighted (T2^*^w) imaging (also termed the “motor band sign”) (16 publications), motor cortex hypointensities (on T1w or T2w imaging, five publications), quantitative susceptibility mapping (two publications), iron deposition (based on T2^*^w contrast, two publications), cerebral microbleeds (one publication), microinfarcts (one publication), and ADC values (one publication).

**Table 1:** Synopsis of brain and spinal cord MRI findings in human motor neuron disease studies.

**Main MRI findings**	**ALS phenotype**	**Evidence level**
**Overall atrophy patterns**		
Total brain volume: total brain volume (17, 18) and brain parenchymal fraction loss (19, 20)	Higher rates in C9+ ALS-FTLD, ALS-FTD	2
Cerebellum: cerebellar atrophy (21, 22); no cerebellar atrophy (23)	ALS/bvFTD and C9+ ALS	1/2
Lobar atrophy: frontal and temporal lobe atrophy (24, 25)		1
Brain stem/spinal cord: brain stem atrophy (26, 27) and spinal cord atrophy (28, 29)		2
Hippocampal volume loss (29–32)		2
**Cortical gray matter volume loss**		
Motor cortex thinning (33–36); no cortical thinning in ALS (17, 32, 37–39)		2
Cortical volume loss in pre- and postcentral gyrus (30) as well as prefrontal regions (40)		2
**Subcortical gray matter volume loss**		
Thalamic volume loss (41–43)	ALS/bvFTD, C9+ ALS, FTD-MND	1
Longitudinal atrophy of the thalamus, caudate nucleus, putamen, amygdala (44), and basal ganglia (45)		4/2
No subcortical volume loss (32, 37–39)	ALS	1
**White matter volume loss**		
White matter volume loss (46)		2
Corpus callosum volume loss (45, 47)	PLS	1
**Signal changes**		
CST hyperintensity in T2w-FLAIR, but also T2w, PDw, T2*w (48, 49)	Advanced stage ALS, PLS	2
Motor cortex hypointensity (motor band sign), on T2w, T2*w, T2w-FLAIR, or SWI (50–53)		2
**Other MRI features**		
Increased iron deposition in deep subcortical gray matter structures, e.g., the caudate nucleus and subthalamic nuclei (54)		1

Imaging findings (including potentially conflicting data) are sorted according to localization/imaging phenotype. Evidence grade is also provided, i.e., the total number of pooled subjects for each individual finding with lower number indicating higher evidence levels (Evidence level 1: >100 cases; 2: 20–100 cases; 3: 10–20 cases; 4: <10 cases).

ALS, amyotrophic lateral sclerosis; C9, C9orf72; CST, corticospinal tract; FLAIR, fluid-attenuated inversion recovery; FTD, frontotemporal dementia (bv, behavioral variant); FTLD, fronto-temporal lobar degeneration; MND, motor neuron disease; PDw, proton density-weighted; PLS, primary lateral sclerosis.

Out of 223 eligible publications, most publications (202, 91%) acquired MRI at field strengths ≤ 3 tesla (T). Seven publications acquired MRI at 7T (3%), including two publications acquiring at both 7 and 3T. The remaining 16 publications did not report on the used static magnetic field strength.

#### CNS atrophy in motor neuron diseases

A majority of publications (166 of 223; 74%) reported whole-brain or regional CNS volume measures. Together, these studies demonstrated volume loss in a wide variety of CNS regions as assessed by several different automated and manual volumetric approaches. We restricted the narrative summary to studies employing automated volumetric segmentation methods and comprising a healthy control group as comparator; comprehensive study findings are reported in Supplementary table 3.

#### Whole-brain and spinal cord atrophy

A reduced total brain volume (17) or reduced brain parenchymal fraction (BPF) have been reported in ALS patients (19, 20). One study only noted BPF reduction in ALS-FTD but not in non-demented ALS (55). One longitudinal study showed higher rates of brain atrophy (around 1.4% per year) in C9orf72+ ALS-FTD patients (18). In ALS, two publications described frontal and temporal lobe atrophy (24, 25). In ALS/bvFTD (56) and C9orf72+ ALS (21, 22), cerebellar atrophy has been reported. This was not confirmed in ALS patients in another study (23). Several publications also described hippocampal volume loss in ALS (41, 57).

In ALS, brain stem atrophy has been shown cross-sectionally (26) and longitudinally (27). Similarly, reduced spinal cord volumes have been observed in ALS (28, 29).

#### Strategic cortical structures

Motor cortex thinning has been described in both ALS and PLS (33, 34), also longitudinally (35). However, two publications did not confirm this finding (17), one of them only observing motor cortex thinning in lower or upper motor neuron dominant ALS phenotypes but not in classical ALS (36). Cortical volume loss in both pre- and postcentral gyri has been shown in two publications (30, 31). Yet, several publications did not observe a difference in cortical volumes between ALS and healthy controls (32, 37–39). One publication described decreased cortical thickness also in prefrontal regions (40). Intriguingly, pre-PLS subjects show subtle thinning of the right precentral gyrus (58).

#### Strategic subcortical gray matter structures

Thalamic volume loss has been described by several publications and in different clinical phenotypes, i.e., ALS/bvFTD (41, 56), C9orf72+ ALS (21), and FTD-MND (42). Longitudinal atrophy of the basal ganglia has been observed in sporadic ALS (45). In ALS, one publication described longitudinal atrophy of several strategic subcortical gray matter structures, including the thalamus, caudate nucleus, putamen and amygdala (44). However, another longitudinal study did not find changes in (subcortical) gray matter volumes in ALS (59). Other studies with a cross-sectional design did not confirm volume loss in subcortical structures in ALS (32, 37–39).

#### Strategic white matter structures

In sporadic ALS, corpus callosum volume loss has been described in one publication (45), while another failed to corroborate the finding (60). Another publication reported subregional corpus callosum volume loss in PLS but not in ALS (47). Volume loss of the CST has been observed in C9orf72+ ALS (22) and sporadic ALS (45). One publication reported on only minimal loss of white matter in ALS (46).

#### Disease phenotypes

Several publications assessed distinctive atrophy features between clinical MND phenotypes/genotypes.

C9orf72+ MND patients seem to show more pronounced atrophy compared to C9orf72- MND patients, e.g., in cortical brain regions (61–63) [such as the motor cortex (64)] or the thalami (62–65). Along these lines, a 5-year follow up study observed more precuneal atrophy in C9orf72+ compared to C9orf72- (66). In contrast, C9orf72+ MND patients showed relative sparing of insular, orbitofrontal, anterior cingulate and temporal pole regions. One publication showed a partly overlapping atrophy pattern between these two genotypes, with the C9orf72+ patients showing volume loss in the accumbens nucleus and C9orf72- patients in the thalami and putamina (67). Interestingly, also presymptomatic C9orf72+ carriers above 40 years of age show more pronounced thalamic, cerebellar, parietotemporal (68), and cervical spinal cord volume loss (69).

Limb onset ALS patients have been shown to have lower cortical volumes in the limb part of the motor homunculus and similarly the corresponding regions for bulbar onset ALS (70). Furthermore, limb onset ALS patients seem to show volume loss in adjacent pre- and postcentral regions. In contrast, bulbar onset ALS seems to have more widespread volume loss, also extending to the bilateral frontotemporal and left superior temporal and supramarginal gyri (71).

ALS and PLS seem to present with distinct atrophy patterns: Whereas ALS patients show more volume loss in the postcentral gyrus, lateral parts of the primary motor cortex, genu of the corpus callosum, amygdala and putamen, PLS patients have an atrophy predominance in the medial parts of the primary motor cortex, splenium of the corpus callosum, cerebellum and thalami (72, 73). One publication described callosal volume loss only in PLS but not in ALS (47).

Along the ALS-FTD spectrum, partly overlapping but also distinct imaging phenotypes based are evident. BvFTD and non-fluent variant primary progressive aphasia (nfvPPA) patients show predominant motor cortex and CST degeneration (74). C9orf72+ ALS-FTD patients show widespread extra-motor pathology and precentral gyrus atrophy compared to ALS patients without cognitive disability. Both bvFTD and ALS exhibit volume loss in orbital and cingulate cortices, CST and corpus callosum, but bvFTD patients have higher orbitofrontal and frontomedial volume loss whereas ALS patients show higher atrophy motor pathways in ALS (75). One study also found widespread volume loss in frontal and temporal regions in FTD-ALS but no volume loss in ALS (76). A more pronounced volume loss in various motor and premotor cortices in ALS-FTD compared to ALS has been confirmed by another publication (77).

It is of note that although three publications did include familial ALS patients (78–80), no imaging data specific for this sub-population was reported and thus, no comparison can be made with sporadic ALS.

### Methodology for CNS brain volumetry

It is noteworthy that eligible publications employed a broad variety of different approaches to quantify CNS volumes. Of 166 publications assessing CNS volumetry, most commonly used automated approaches were SPM (various versions, 44 publications, 27%), FreeSurfer (36 publications, 22%) and FSL (35 publications, 21%). Twenty-four publications used visual rating of atrophy or manual segmentation of regions of interests (14%). Five publications did not report how they quantified CNS volumes.

Due to considerable heterogeneity in the methodological approach to quantify CNS volumes, as well as the various clinical MND phenotypes, we deemed it unfeasible to perform a valid meta-analysis of atrophy patterns of MND patients.

#### Corticospinal tract hyperintensities

**Figure 2:**
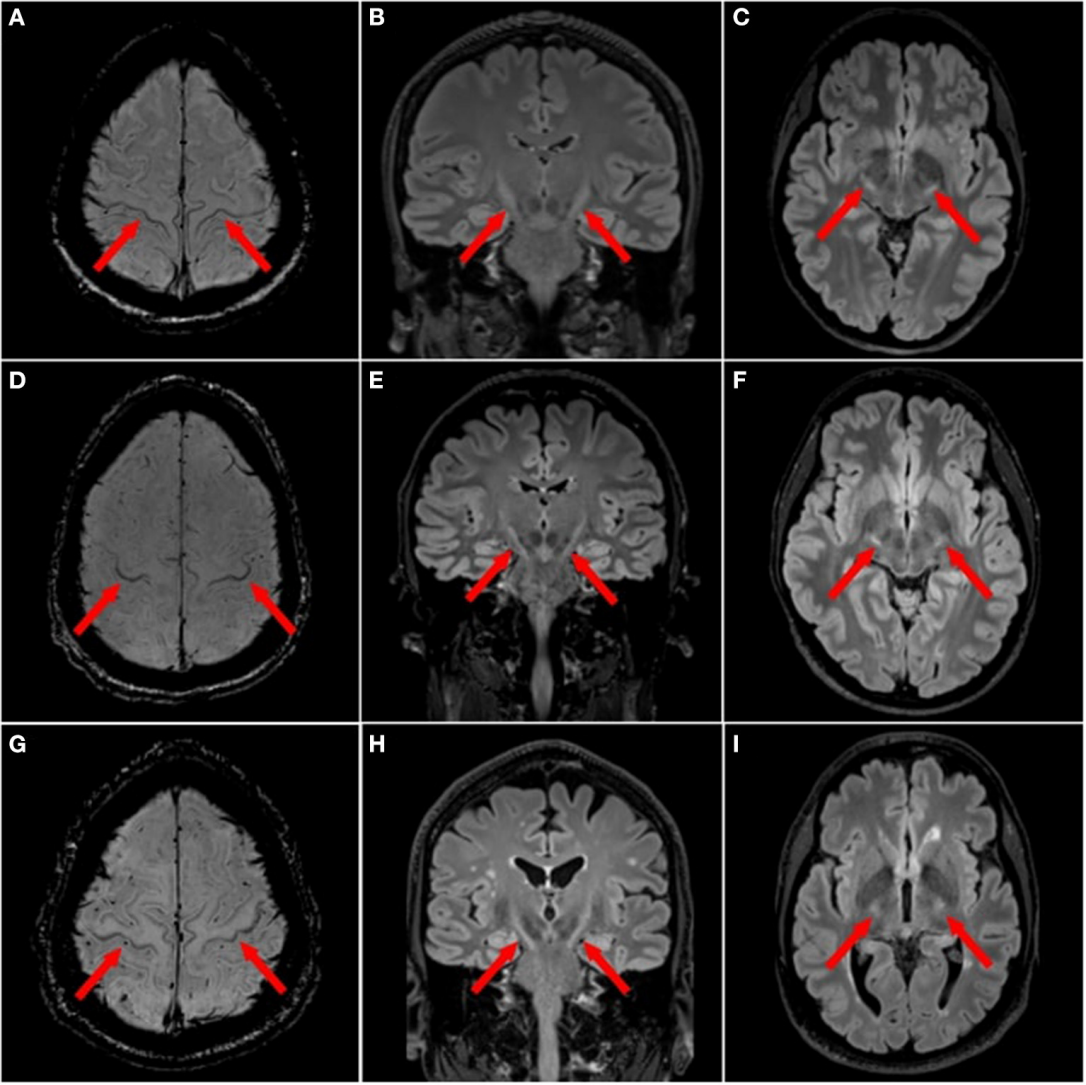
Focal magnetic resonance imaging sign in motor neuron diseases. ALS patients with motor cortex hypointensities, also termed “motor band sign”, on 3T magnetic resonance imaging (MRI) with axial susceptibility weighted imaging (SWI) [**(A,D,G)** red arrows] and T2 hyperintensities along the corticospinal tract (CST) on 3T 3D T2w-FLAIR in the axial [**(B,E,H)** red arrows] and coronal planes **(C,F,I)**.

In MND, hyperintensities have been observed along the entire CST projection (Figure 2), i.e., in the corona radiata, internal capsule, cerebral peduncles and pons (48). The diagnostic role of these CST hyperintensities, as seen on T2w, PDw or T2^*^w imaging, is conflicting. Some publications found them to be present at higher proportions in ALS patients compared to healthy controls (49), while others failed to confirm this finding (50, 81). Interestingly, CST signal abnormalities were more common in advanced stage ALS (63%) and PLS (72%) compared to early stage ALS (17%) (82). Yet the proportion of these CST signal abnormalities among ALS patients varies substantially (31, 83, 84).

The variability of CST signal abnormalities across different MND phenotypes is less clear. One study, including 21 non-demented ALS patients and 3 demented ALS patients, found 29% of non-demented ALS patients with CST hyperintensities, while none of the three demented ALS patients demonstrated this imaging feature (85). One publication comprising 122 non-ALS patients confirms that these CST hyperintensities are not specific to ALS and that their frequency increases with age (86).

One publication in ALS found a diagnostic specificity of 76% and a sensitivity of 48% for this sign, albeit with higher specificity in the subcortical white matter, centrum semiovale and medullary pyramids (87). A comparative multi-sequence study found T2w-FLAIR as the most sensitive sequence to detect CST hyperintensities in comparison with T1w, T2w and PDw. T2w-FLAIR could even capture a longitudinal increase in CST signal intensity (88). However, T2w-FLAIR showed high inter-rater agreement for evaluating CST hyperintensities (89).

**Figure 3:**
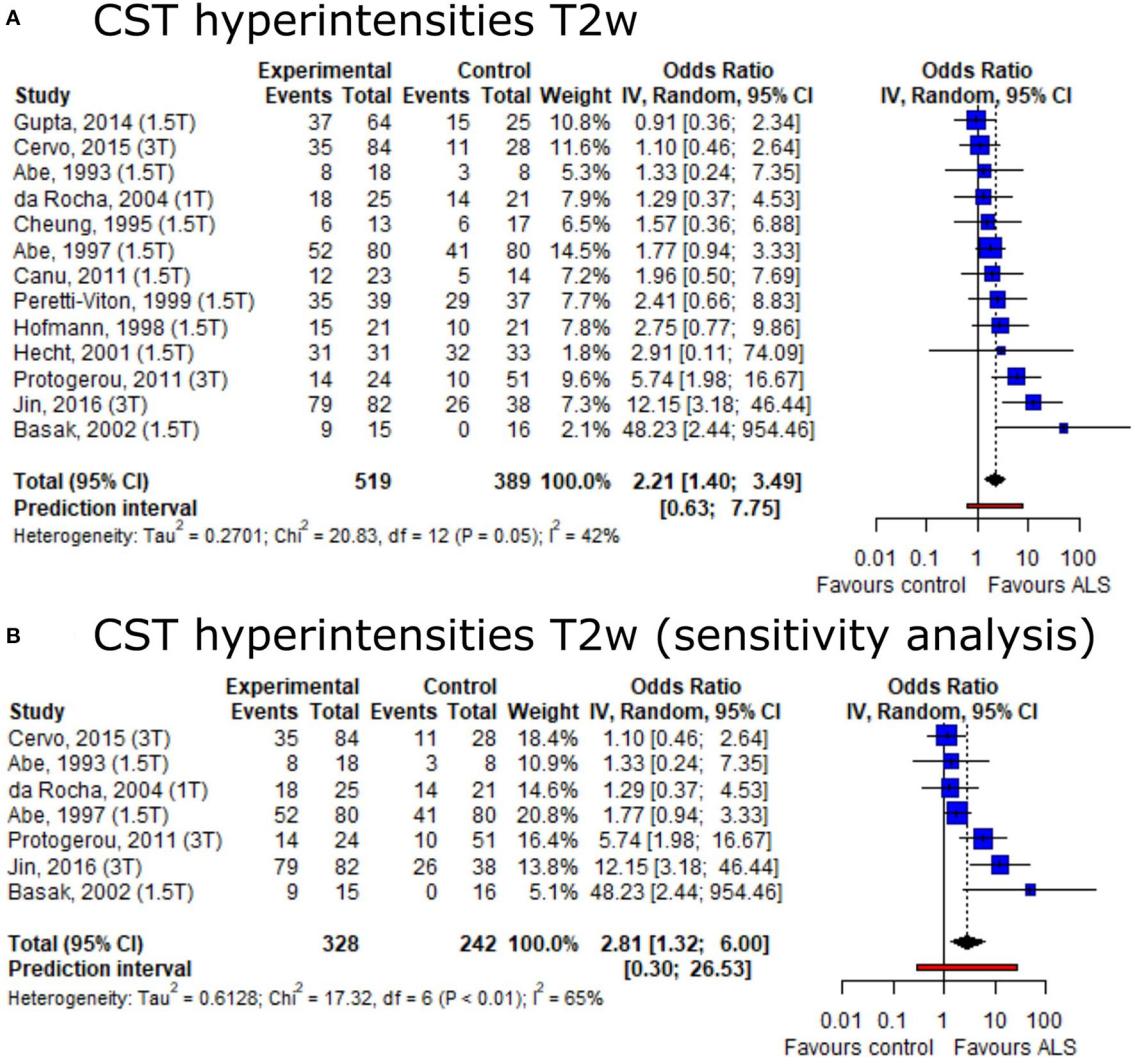
Forest plot of corticospinal tract hyperintensities on T2w MRI odds ratios among ALS patients vs. controls. Pooled analyses of publications comparing odds ratios (OR) of corticospinal tract (CST) hyperintensities on T2w MRI in ALS patients and controls **(A)** and a sensitivity analysis only including publications with low risk of bias and comprising healthy controls **(B)**. Static magnetic field strength for MRI acquisition for respective publications are listed in brackets. Dotted line represents overall mean; black diamond represents overall confidence interval. CI, confidence interval; CST, corticospinal tract.

Based on the conflicting data on the presence of CST hyperintensities among ALS patients and healthy controls, we set out to provide summary estimates for odds ratios (OR). The meta-analysis of CST hyperintensities on T2w image contrast showed an overall OR of 2.21 (95%-CI: 1.40–3.49) (including 13 publications, n_ALS_ = 519, n_Ctrl_ = 389) (Figure 3A). The direction of effect remained in a sensitivity analysis only including studies with low risk of bias and comprising a healthy control group (Figure 3B). Neither the Egger's regression test nor the rank correlation test suggested publication bias (*p* = 0.10 and 0.25, respectively).

#### Motor cortex hypointensity

In ALS, motor cortex hypointensity, also termed the “motor band sign” or “black ribbon sign”, can be observed on T2w, T2^*^w, T2w-FLAIR or SWI (Figure 2). The proportion of ALS patients displaying a motor band sign varies considerably in the literature: reported proportion ranges from over 90% (on T2^*^w) (51), to much lower numbers (52). Some publications reported higher motor band sign proportions on T2w contrast in ALS patients compared to healthy controls (50). This sign also seems to persist longitudinally (51). One study in 25 upper motor neuron ALS and 23 healthy controls found 100% specificity and 20% sensitivity for the motor band sign (53).

SWI has been reported as the most sensitive imaging approach to detect the motor band sign (vs. T2w and T2^*^w) (90). This was confirmed by another study reporting that 78% of ALS patients were deemed motor sign band positive on SWI compared to only 5% of patients on clinical routine imaging (i.e., T2w, T2^*^w, T2w-FLAIR, DWI) (91). Another study found that T2^*^w imaging was superior to other deployed sequences (not including SWI) (92). Also, ultra-high-field imaging (7T) seems to have a higher sensitivity to capture the motor band sign compared to imaging at 3T (93).

**Figure 4:**
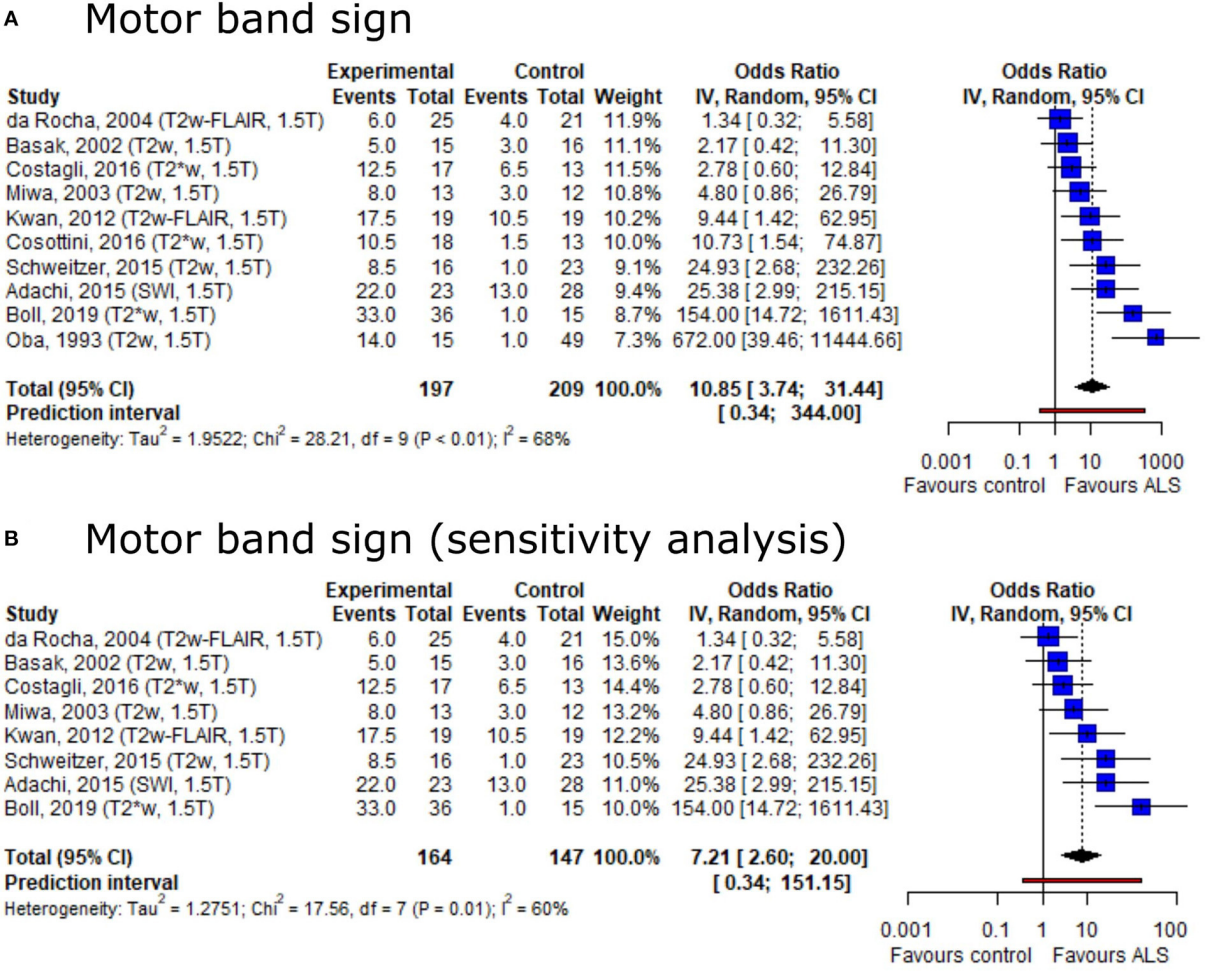
Forest plot of motor band sign odds ratios among ALS patients vs. controls. Pooled analyses of publications comparing odds ratios (OR) of the motor band sign in ALS patients and controls **(A)** and a sensitivity analysis only including publications with low risk of bias and comprising healthy controls **(B)**. Static magnetic field strength for MRI acquisition as well as employed MRI sequence for respective publications are listed in brackets. OR for motor band sign was extracted and pooled using the random effects DerSimonian-Laird method. Dotted line represents study means; black diamond represents overall confidence interval. CI, confidence interval.

Based on the conflicting result on motor band sign proportions among ALS patients and healthy controls, we set out to provide summary estimates for odds ratios (OR). The meta-analysis of motor band sign showed an overall OR of 10.85 (95%-CI: 3.74–31.44) (including 10 publications, n_ALS_ = 197, n_Ctrl_ = 209) with substantial heterogeneity across publications (*I*^2^ = 68%, *p* < 0.01) (Figure 4A). The direction of the effect remained in a sensitivity analysis only including publications with low risk of bias and comprising a healthy control group (Figure 4B). Both the Egger's regression test and the rank correlation test indicated publication bias (*p* < 0.001 for both tests).

#### Other MRI features

One study employing T2^*^w contrast at 7T did not find cerebral microbleeds in ALS (94). Yet, a post-mortem study at 7T comprising 72 cases found increased numbers of cortical cerebral microbleeds in frontal and temporal lobes of ALS compared to non-ALS controls (95). The latter study also found increased iron levels in deep subcortical gray matter structures in ALS brains, such as the caudate nucleus and subthalamic nuclei, compared to healthy controls and subjects with other neurodegenerative diseases (54). Interestingly, in the same study, cortical microinfarcts were more common in healthy controls compared to ALS.

### Magnetic resonance imaging histopathology correlation

Fourteen publications presented concomitant histopathology data, but only seven of them directly correlated MRI findings with histopathology. Three publications assessed the tissue signature of CST/white matter hyperintensities in ALS, comprising a total of 15 brains from ALS patients. These publications consistently reported myelin pallor, gliosis as well as depletion of (large) axons, albeit at different CNS locations, i.e., internal capsule (96, 97) or temporal subcortical white matter (85). This latter study investigated one brain from a demented ALS patient and observed severe neuronal loss and gliosis in the adjacent temporal cortex.

Three publications assessed the underlying histopathology of low signal in the motor cortex and included a total of 10 brains from ALS patients. All studies found iron accumulation in the deep cortical layers (98), sometimes located within astrocytes and/or microglia/macrophages (90, 99).

Finally, one study aimed at predicting motor cortex neuron density based on white matter volume estimates. The authors indeed reported a linear function modeling motor neuron density, albeit only in a subgroup of sporadic ALS patients (100).

## Discussion

### Main findings

The main objective of this study was to systematically summarize the available evidence on structural MRI features among the MND spectrum to provide radiologists and other clinicians with an up-to-date guide to facilitate workup of suspected MND cases. MND patients indeed exhibit brain/spinal cord atrophy on MRI, and partly distinguishing atrophy patterns are evident along the phenotypic spectrum of MND (Table 1). Additionally, ALS patients may present with T2w hyperintensities along the CST course and signal alterations in the motor cortex (motor band sign). Both features are more prevalent in ALS compared to controls as corroborated by our meta-analysis. Although both of these imaging features are not specific to ALS, they can be leveraged for differential diagnosis in conjunction with other imaging features such as certain brain atrophy patterns. Pathological studies found axonal loss and myelin pallor congruent with the CST hyperintensities and iron deposition as well as glial activation in the deeper layers of the motor cortex congruent with the motor band sign.

### Findings in the context of existing evidence

Volume loss of CNS tissue, as seen in MND, is a widely observed imaging feature in other neurodegenerative diseases such as Alzheimer's disease (101), Parkinson's disease (102) or multiple sclerosis (MS) (103) and it is also a normal process of the aging brain (104). However, distinct atrophy patterns could still provide clues during the diagnostic workup of suspected MND cases. One such clue could be the more distinct atrophy pattern of the motor system in ALS, such as the descending motor tracts of the spinal cord including the CST, or the more pronounced atrophy in premotor cortices in ALS-FTD. Nevertheless, prior studies have correctly pointed out that a single modality such as structural MRI will likely be insufficient to clearly discriminate MND phenotypes and more advanced multimodal neuroimaging techniques are likely to be required (9, 105).

The overall evidence has reported a large and partly inconsistent variety of brain regions being affected by volume loss in MND. One potential explanation for this partly inconsistent data is the substantial variability in methodological approaches to quantify brain volume loss, e.g., voxel-based morphometry approaches (such as FSL) vs. surface-based analysis tools (such as FreeSurfer). This has also been emphasized by a study comparing different software to assess cortical volumes in ALS-FTD patients (FSL, FreeSurfer and SPM), which yielded variable results depending on deployed software (106). Additional confounders exist in the form of technical parameters (e.g., intra-/inter-scanner variability) and physiological factors (e.g. state of hydration in scanned subjects) [reviewed by Sastre-Garriga et al. (107)]. Except for one small study, which aimed at predicting cortical motor neuron density by MRI, our systematic search did not identify any study investigating the tissue signature of cortical or subcortical parenchyma loss as it has been done for example in MS (108). Having such insight into tissue pathology from imaging could further strengthen our understanding of the intricate pathogenesis of neurodegeneration in MND (109).

In our meta-analysis, CST hyperintensities could not be established as a specific MND feature. White matter hyperintensities are a common finding in many neurological diseases, such as MS, CNS vasculitis, leukodystrophy, neuromyelitis optica spectrum disorder (NMOSD), Susac's syndrome or cerebrovascular disease (110). However, the more distinct distribution of CST hyperintensities in ALS along the CST could still be useful during diagnostic work up. Interestingly, these hyperintensities have consistently been shown to correlate to axonal degeneration and demyelination at the tissue level (97). Also the motor band sign is not a specific imaging hallmark for ALS: It has been shown in Alzheimer's and Parkinson's disease patients, as well as in healthy controls (111). At least in ALS, these signal drops seem to correspond to astro- and microglia iron deposition within deep layers of the motor cortex (98). It is unclear if patients with such imaging features without clinical signs of MND might have similar pathological findings. Together, the imaging features, as synthesized in this systematic review, can improve the usefulness of MRI in the diagnostic work up of MNDs in the clinical setting. Nevertheless, more studies are needed to corroborate the diagnostic use in clinical routine, particularly when pooling these imaging signs together for clinical reads. These findings, especially if quantified objectively and complemented with advanced multimodal neuroimaging techniques, could potentially also prove valuable as outcome measures or means of therapeutic monitoring in a setting of increasing efforts to find disease-modifying treatments for MND. For this, their clinical prognostic value remains to be studied more in-depth.

## Limitations

First, for assessing OR of focal MRI features in MND, we pooled studies with various methodological backgrounds for summary estimates. Although we mitigated the potential of bias by applying a random effects meta-analysis model, residual bias might skew the analysis. Second, there was considerable heterogeneity in the motor neuron disease patient cohorts between publications which could skew the interpretation of the narrative synthesis. Third, both T2w CST hyperintensities and the motor band sign have large prediction intervals, providing only a crude estimate of where effect sizes of future studies could be expected. However, these can be helpful for planning future studies addressing these imaging signs in MND.

## Conclusions

This study provides high level evidence on structural MRI features among the MND spectrum. Our resource is useful for radiologists and other clinicians during diagnostic workup of suspected MND cases. These structural imaging features can complement advanced multimodal neuroimaging to enhance discriminatory power among the MND spectrum. We also provide a summary of the available evidence on the corresponding pathological tissue signature of these MRI features. Future studies warrant a more precise definition of the sensitivity and specificity of these findings in different MND subtypes with relevant controls. In addition, systematic reviews/meta-analyses of brain atrophy patterns among a wider range of neurodegenerative diseases can serve to further improve the usefulness of MRI for differential diagnostic workup and/or therapeutic monitoring.

## Data availability statement

The original contributions presented in the study are included in the article/Supplementary material, further inquiries can be directed to the corresponding author/s.

## Author contributions

CZ, TG, and BI conceived the study. CZ, DN, and BI performed the literature review and data extraction. BI performed the meta-analysis. CZ, FP, TG, and BI wrote the manuscript. DN, SW, AP, SL, JF, ZK, FP, and CI provided critical input on the manuscript. All authors contributed to the article and approved the submitted version.

## Funding

This study was supported by the Swiss National Science Foundation (Grant No. P400PM_183884, to BI) as well as Region Stockholm and CIMED (to TG). None of the funders had any role in study design, data collection and analysis, decision to publish, or preparation of the manuscript.

## Conflict of interest

The authors declare that the research was conducted in the absence of any commercial or financial relationships that could be construed as a potential conflict of interest.

## Publisher's note

All claims expressed in this article are solely those of the authors and do not necessarily represent those of their affiliated organizations, or those of the publisher, the editors and the reviewers. Any product that may be evaluated in this article, or claim that may be made by its manufacturer, is not guaranteed or endorsed by the publisher.

## Abbreviations

ADC: apparent diffusion coefficient
ALS: amyotrophic lateral sclerosis
CNS: central nervous system
CST: corticospinal tract
FTD, frontotemporal dementia (bv: behavioral variant)
FTLD: frontotemporal lobe degeneration
MND: motor neuron disease
MRI: magnetic resonance imaging
PLS: primary lateral sclerosis
PMA: progressive muscular atrophy
PPA: primary progressive aphasia
QSM: quantitative susceptibility mapping
SWI: susceptibility-weighted imaging.
