# Supplementary material for: Structural magnetic resonance imaging findings and histopathological correlations in motor neuron diseases—A systematic review and meta-analysis
Source: Front Neurol. 2022 Aug 30;13:947347. doi: 10.3389/fneur.2022.947347 (PMC9468579; doi:10.3389/fneur.2022.947347)
Supplement: Supplementary file 1 [file Table_1.DOCX]

**Structural magnetic resonance imaging findings and histopathological correlations in human and animal motor neuron diseases – a systematic review and meta-analysis**

**Supplementary data**

**Supplementary Table 1**: Predefined systematic search strings in Medline via PubMed, Ovid EMBASE, and Web of Science. Last search April 8, 2021. The search retrieved 5941 unique publications.

| **Databases** | **Studies produced** | **Search string** |
| --- | --- | --- |
| **Pubmed via Medline:** | 3656 | 1 exp Motor Neuron Disease/  2 (als or bulbar pals* or bulbar paralys* or fazio londe or hereditary motor neuronopath* or kenned* syndrom* or lateral scleros* or spinal amyotroph*).ti,ab,kf.  3 ((anterior horn cell or charcot* or gehrig* or guam or kenned* or kugelberg welander or lou gehrig* or motor neuron or motor system or werdnig hoffmann) adj3 (disease* or syndrom*)).ti,ab,kf.  4 ((bulbar or bulbospinal or bulbo spinal or muscular or spinal or spinobulbar or spino bulbar) adj3 atroph*).ti,ab,kf.  5 or/1-4  6 exp Neuroimaging/  7 exp Magnetic Resonance Imaging/  8 exp Positron-Emission Tomography/  9 (brain imaging* or brain mapping or brain electrical activity mapping or cerebral angiograph* or connectome* or chemical shift imaging* or ct pet or diffusion tractograph* or diffusion tensor or dti or echo planar imaging* or echoplanar imaging* or functional cerebral localization* or functional cerebral localisation* or fmri or magnetic resonance imaging* or magnetic resonance angiograph* or magnetization transfer contrast imaging* or mpmri or mr tomograph* or mri or mris or neuroimaging* or neuroradiography or nmr imaging or nmr tomograph* or pet scan* or pet ct scan or positron emission tomograph* or proton spin tomograph* or single photon emission computed tomograph* or spect or spectroscop* or spin echo imaging* or voxel based morphometr* or zeugmatograph*).ti,ab,kf.  10 or/6-9  11 5 and 10  12 limit 11 to english language |
| **EMBASE:** | 3577 | ('motor neuron disease'/exp OR als:ti,ab,kw OR 'bulbar pals*':ti,ab,kw OR 'bulbar paralys*':ti,ab,kw OR 'fazio londe':ti,ab,kw OR 'hereditary motor neuronopath*':ti,ab,kw OR 'kenned* syndrom*':ti,ab,kw OR 'lateral scleros*':ti,ab,kw OR 'spinal amyotroph*':ti,ab,kw OR ((('anterior horn cell' OR charcot* OR gehrig* OR guam OR kenned* OR 'kugelberg welander' OR 'lou gehrig*' OR 'motor neuron' OR 'motor system' OR 'werdnig hoffmann') NEAR/3 (disease* OR syndrom*)):ti,ab,kw) OR (((bulbar OR bulbospinal OR 'bulbo spinal' OR muscular OR spinal OR spinobulbar OR 'spino bulbar') NEAR/3 atroph*):ti,ab,kw))  AND  ('neuroimaging'/exp OR 'nuclear magnetic resonance imaging'/exp OR 'positron emission tomography'/exp OR 'brain imaging*':ti,ab,kw OR 'brain mapping':ti,ab,kw OR 'brain electrical activity mapping':ti,ab,kw OR 'cerebral angiograph*':ti,ab,kw OR connectome*:ti,ab,kw OR 'chemical shift imaging*':ti,ab,kw OR 'ct pet':ti,ab,kw OR 'diffusion tractograph*':ti,ab,kw OR 'diffusion tensor':ti,ab,kw OR dti:ti,ab,kw OR 'echo planar imaging*':ti,ab,kw OR 'echoplanar imaging*':ti,ab,kw OR 'functional cerebral localization*':ti,ab,kw OR 'functional cerebral localisation*':ti,ab,kw OR fmri:ti,ab,kw OR 'magnetic resonance imaging*':ti,ab,kw OR 'magnetic resonance angiograph*':ti,ab,kw OR 'magnetization transfer contrast imaging*':ti,ab,kw OR mpmri:ti,ab,kw OR 'mr tomograph*':ti,ab,kw OR mri:ti,ab,kw OR mris:ti,ab,kw OR neuroimaging*:ti,ab,kw OR neuroradiography:ti,ab,kw OR 'nmr imaging':ti,ab,kw OR 'nmr tomograph*':ti,ab,kw OR 'pet scan*':ti,ab,kw OR 'pet ct scan':ti,ab,kw OR 'positron emission tomograph*':ti,ab,kw OR 'proton spin tomograph*':ti,ab,kw OR 'single photon emission computed tomograph*':ti,ab,kw OR spect:ti,ab,kw OR spectroscop*:ti,ab,kw OR 'spin echo imaging*':ti,ab,kw OR 'voxel based morphometr*':ti,ab,kw OR zeugmatograph*:ti,ab,kw)  AND  [english]/lim AND ('article'/it OR 'article in press'/it) |
| **Web of Science:** | 3338 | TS=(”bulbar pals*” OR ”bulbar paralys*” OR ”fazio londe” OR ”hereditary motor neuronopath*” OR ”kenned* syndrom*” OR ”lateral scleros*” OR ”spinal amyotroph*”) OR TS=((”anterior horn cell” OR charcot* OR gehrig* OR guam OR kenned* OR ”kugelberg welander” OR ”lou gehrig*” OR ”motor neuron” OR ”motor system” OR ”werdnig hoffmann”) NEAR/3 (disease* OR syndrom*)) OR TS=((bulbar OR bulbospinal OR ”bulbo spinal” OR muscular OR spinal OR spinobulbar OR ”spino bulbar”) NEAR/3 atroph*)  AND  TS=(”brain imaging*” OR ”brain mapping” OR ”brain electrical activity mapping” OR ”cerebral angiograph*” OR connectome* OR ”chemical shift imaging*” OR ”ct pet” OR ”diffusion tractograph*” OR ”diffusion tensor” OR dti OR ”echo planar imaging*” OR ”echoplanar imaging*” OR ”functional cerebral localization*” OR ”functional cerebral localisation*” OR fmri OR ”magnetic resonance imaging*” OR ”magnetic resonance angiograph*” OR ”magnetization transfer contrast imaging*” OR mpmri OR ”mr tomograph*” OR "mri" OR "mris" OR neuroimaging* OR neuroradiography OR ”nmr imaging” OR ”nmr tomograph*” OR ”pet scan*” OR ”pet ct scan” OR ”positron emission tomograph*” OR ”proton spin tomograph*” OR ”single photon emission computed tomograph*” OR spect OR spectroscop* OR ”spin echo imaging*” OR ”voxel based morphometr*” OR zeugmatograph*)  Refined by: LANGUAGES: ( ENGLISH )  Indexes=SCI-EXPANDED, SSCI, A&HCI, CPCI-S, CPCI-SSH, ESCI Timespan=All years |

**Supplementary table 2**: Risk of bias assessment of the included studies with 10 or more included human subjects according to the Newcastle-Ottawa scale for nonrandomized studies[[1](#_ENREF_1)]. S1-S4: Selection domain (only S1-S3 for cohort studies, case series and retrospective studies), C1/2: comparability domains, E1-E2: exposure domain. In alphabetical order and sorted for disease class.

| **Author, year** | **Title** | **S1:** | **S2** | **S3** | **S4** | **C1/2** | **E1** | **E2** |
| --- | --- | --- | --- | --- | --- | --- | --- | --- |
| Abdulla, 2014 | Hippocampal degeneration in patients with amyotrophic lateral sclerosis | * | * | - | * | * | - | * |
| Abe, 1997 | Degeneration of the pyramidal tracts in patients with amyotrophic lateral sclerosis. A premortem and postmortem magnetic resonance imaging study | * | - | - | * | * | * | * |
| Abe, 1993 | Single-photon emission computed tomographic investigation of patients with motor neuron disease | * | * | * | * | * | * | * |
| Abrahams, 2001 | Executive dysfunction in amyotrophic lateral sclerosis (ALS); a functional and structural MRI study | - | - | - | * | * | - | * |
| Abrahams, 2005 | Frontotemporal white matter changes in amyotrophic lateral sclerosis | * | * | * | * | * | - | * |
| Acosta-Cabronero, 2018 | Quantitative Susceptibility MRI to Detect Brain Iron in Amyotrophic Lateral Sclerosis | * | - | * | * | * | - | * |
| Adachi, 2015 | Usefulness of SWI for the Detection of Iron in the Motor Cortex in Amyotrophic Lateral Sclerosis | * | - | - | * | * | * | * |
| Agosta, 2016 | Structural brain correlates of cognitive and behavioral impairment in MND | * | * | * | * | * | * | * |
| Agosta, 2017 | Structural and functional brain signatures of C9orf72 in motor neuron disease | * | * | * | * | * | - | * |
| Agosta, 2015 | MRI signatures of the frontotemporal lobar degeneration continuum | * | * | * | - | - | * | * |
| Agosta, 2009 | Longitudinal assessment of grey matter contraction in amyotrophic lateral sclerosis: A tensor based morphometry study | * | - | - | * | * | - | * |
| Agosta, 2007 | Voxel-based morphometry study of brain volumetry and diffusivity in amyotrophic lateral sclerosis patients with mild disability | * | - | - | * | - | * | * |
| Agosta, 2019 | Survival prediction models in motor neuron disease | * | * | N/A | N/A | N/A | * | * |
| Agosta , 2012 | The cortical signature of amyotrophic lateral sclerosis | * | * | - | * | * | - | * |
| Alruwaili, 2018 | A combined tract-based spatial statistics and voxel-based morphometry study of the first MRI scan after diagnosis of amyotrophic lateral sclerosis with subgroup analysis | * | * | * | * | - | - | * |
| Ambikairajah, 2014 | A visual MRI atrophy rating scale for the amyotrophic lateral sclerosis-frontotemporal dementia continuum | * | * | * | * | * | * | * |
| Andreadou, 1998 | Subcortical frontal lesions on MRI in patients with motor neurone disease | * | - | - | - | * | - | * |
| Ash, 2014 | Narrative discourse deficits in amyotrophic lateral sclerosis | * | - | - | * | * | - | * |
| Bae, 2016 | Dissociation of Structural and Functional Integrities of the Motor System in Amyotrophic Lateral Sclerosis and Behavioral-Variant Frontotemporal Dementia | * | - | * | * | * | - | * |
| Basak, 2002 | Magnetic resonance imaging in amyotrophic lateral sclerosis | * | - | * | - | * | * | * |
| Bede, 2013 | The Neuroimaging Signature of the C9orf72 Hexanucleotide Repeat in Amyotrophic Lateral Sclerosis - A Multimodal MRI Study | * | * | - | * | * | - | * |
| Bede, 2013 | Grey Matter Correlates of Clinical Variables in Amyotrophic Lateral Sclerosis - A Neuroimaging Study of ALS Motor Phenotype Heterogeneity and Cortical Focality | * | * | * | * | * | - | * |
| Bede, 2013 | Basal ganglia involvement in amyotrophic lateral sclerosis | * | - | - | * | * | - | * |
| Bede, 2018 | Longitudinal structural changes in ALS: a three time-point imaging study of white and gray matter degeneration | * | - | - | * | * | - | * |
| Bede, 2018 | Connectivity-based characterisation of subcortical grey matter pathology in frontotemporal dementia and ALS: a multimodal neuroimaging study | * | - | - | * | * | - | * |
| Bocchetta, 2018 | Thalamic atrophy in frontotemporal dementia — Not just a C9orf72 problem | - | - | - | * | * | - | * |
| Bocchetta, 2020 | Thalamic nuclei in frontotemporal dementia: Mediodorsal nucleus involvement is universal but pulvinar atrophy is unique to C9orf72 | - | - | - | * | * | - | * |
| Boll, 2019 | Is the Hypointensity in Motor Cortex the Hallmark of Amyotrophic Lateral Sclerosis? | * | * | * | * | * | * | * |
| Branco, 2018 | Brain signature of mild stages of cognitive and behavioral impairment in amyotrophic lateral sclerosis | * | * | - | * | * | * | * |
| Buhour, 2017 | Voxel-based mapping of grey matter volume and glucose metabolism profiles in amyotrophic lateral sclerosis | * | - | * | * | * | - | * |
| Butman, 2007 | Decreased thickness of primary motor cortex in primary lateral sclerosis | * | - | - | * | * | * | * |
| Canu, 2011 | The topography of brain microstructural damage in amyotrophic lateral sclerosis assessed using diffusion tensor MR imaging | * | - | - | * | - | * | * |
| Cardenas-Blanco, 2016 | Structural and diffusion imaging versus clinical assessment to monitor amyotrophic lateral sclerosis | * | * | N/A | N/A | * | * | * |
| Carella, 1995 | Magnetic resonance signal abnormalities along the pyramidal tracts in amyotrophic lateral sclerosis | - | N/A | N/A | N/A | N/A | * | * |
| Cerami, 2014 | Emotional empathy in amyotrophic lateral sclerosis: a behavioural and voxel-based morphometry study | * | * | * | * | * | * | * |
| Cervo, 2015 | The combined use of conventional MRI and MR spectroscopic imaging increases the diagnostic accuracy in amyotrophic lateral sclerosis | * | - | - | * | * | * | * |
| Chang, 2005 | A voxel-based morphometry study of patterns of brain atrophy in ALS and ALS/FTLD | * | * | - | * | - | - | * |
| Chapman, 2012 | Corpus callosum area in amyotrophic lateral sclerosis | * | - | * | * | * | - | * |
| Charil, 2009 | Structural and metabolic changes in the brain of patients with upper motor neuron disorders: a multiparametric MRI study | * | * | - | * | * | * | * |
| Chen, 2018 | MR Imaging-based Estimation of Upper Motor Neuron Density in Patients with Amyotrophic Lateral Sclerosis: A Feasibility Study | * | * | N/A | N/A | N/A | - | - |
| Chen, 2018 | Cortical Thinning Pattern of Bulbar- and Spinal-onset Amyotrophic Lateral Sclerosis: a Surface-based Morphometry Study |  |  |  |  |  |  |  |
| Cheung, 1995 | Amyotrophic lateral sclerosis: correlation of clinical and MR imaging findings | * | * | - | * | * | * | * |
| Christidi, 2019 | Hippocampal pathology in amyotrophic lateral sclerosis: selective vulnerability of subfields and their associated projections | * | - | - | * | - | * | * |
| Christidi, 2018 | Gray matter and white matter changes in non-demented amyotrophic lateral sclerosis patients with or without cognitive impairment: A combined voxel-based morphometry and tract-based spatial statistics whole-brain analysis | * | * | - | * | * | * | * |
| Christidi, 2018 | Motor and extra-motor gray matter integrity may underlie neurophysiologic parameters of motor function in amyotrophic lateral sclerosis: a combined voxel-based morphometry and transcranial stimulation study | * | * | - | * | * | * | * |
| Clark, 2018 | Loss of functional connectivity is an early imaging marker in primary lateral sclerosis | * | * | - | * | * | * | * |
| Cohen-Adad, 2013 | Involvement of spinal sensory pathway in ALS and specificity of cord atrophy to lower motor neuron degeneration | * | * | * | * | * | - | * |
| Consonni, 2019 | Cognitive Syndromes and C9orf72 Mutation Are Not Related to Cerebellar Degeneration in Amyotrophic Lateral Sclerosis | * | - | - | * | * | - | * |
| Consonni, 2019 | Cortical correlates of behavioural change in amyotrophic lateral sclerosis | * | * | N/A | N/A | * | - | * |
| Consonni, 2018 | Cortical markers of cognitive syndromes in amyotrophic lateral sclerosis | * | * | * | * | * | - | * |
| Contarino, 2020 | Toward a marker of upper motor neuron impairment in amyotrophic lateral sclerosis: A fully automatic investigation of the magnetic susceptibility in the precentral cortex | * | - | * | * | * | * | * |
| Coon, 2011 | Predicting survival in frontotemporal dementia with motor neuron disease | * | * | N/A | N/A | N/A | - | - |
| Coon, 2012 | Right temporal variant frontotemporal dementia with motor neuron disease | * | * | N/A | N/A | N/A | * | * |
| Cosottini, 2013 | Mapping cortical degeneration in ALS with magnetization transfer ratio and voxel-based morphometry | * | * | - | * | * | - | * |
| Cosottini, 2016 | High-Resolution 7T MR Imaging of the Motor Cortex in Amyotrophic Lateral Sclerosis | * | - | - | - | * | * | * |
| Cosottini, 2012 | Structural and functional evaluation of cortical motor areas in Amyotrophic Lateral Sclerosis | * | * | - | * | * | - | * |
| Costagli, 2016 | Magnetic susceptibility in the deep layers of the primary motor cortex in Amyotrophic Lateral Sclerosis | * | - | - | * | * | * | * |
| Crespi, 2018 | Multimodal MRI quantification of the common neurostructural bases within the FTD-ALS continuum | * | - | N/A | N/A | * | * | * |
| d'Ambrosio, 2014 | Frontotemporal cortical thinning in amyotrophic lateral sclerosis | * | - | - | * | * | * | * |
| da Rocha, 1999 | Magnetic resonance findings in amyotrophic lateral sclerosis using a spin echo magnetization transfer sequence. Preliminary report | - | - | N/A | N/A | N/A | * | N/A |
| da Rocha, 2004 | Detection of corticospinal tract compromise in amyotrophic lateral sclerosis with brain MR imaging: relevance of the T1-weighted spin-echo magnetization transfer contrast sequence | * | - | - | * | * | * | * |
| de Albuguergue, 2016 | Multimodal Longitudinal MRI Study in Amyotrophic Lateral Sclerosis (ALS) |  |  |  |  |  |  |  |
| de Albuguergue, 2016 | MRI Texture Analysis Reveals Deep Gray Nuclei Damage in Amyotrophic Lateral Sclerosis | * | * | * | * | * | * | * |
| de Albuguergue, 2017 | Longitudinal evaluation of cerebral and spinal cord damage in Amyotrophic Lateral Sclerosis | * | * | - | * | * | * | * |
| De Marco, 2015 | Morphometric correlates of dysarthric deficit in amyotrophic lateral sclerosis | * | * | - | * | * | * | * |
| De Reuck, 2017 | Topographic distribution of brain iron deposition and small cerebrovascular lesions in amyotrophic lateral sclerosis and in frontotemporal lobar degeneration: a post-mortem 7.0-tesla magnetic resonance imaging study with neuropathological correlates | * | - | - | * | - | - | * |
| De Reuck, 2014 | Iron deposits in post-mortem brains of patients with neurodegenerative and cerebrovascular diseases: a semi-quantitative 7.0 T magnetic resonance imaging study | * | - | - | * | - | - | * |
| Devenney, 2014 | Frontotemporal dementia associated with the C9ORF72 mutation: a unique clinical profile | * | * | N/A | N/A | * | * | * |
| Devenney, 2017 | The neural correlates and clinical characteristics of psychosis in the frontotemporal dementia continuum and the C9orf72 expansion | * | * | * | * | * | - | * |
| Devine, 2015 | Exposing asymmetric gray matter vulnerability in amyotrophic lateral sclerosis | * | - | - | * | * | - | * |
| Ding, 2011 | Value of quantitative analysis of routine clinical MRI sequences in ALS | * | - | - | * | * | * | * |
| Donatelli, 2019 | MRI cortical feature of bulbar impairment in patients with amyotrophic lateral sclerosis | * | * | N/A | N/A | - | * | - |
| Donatelli, 2018 | Semiautomated Evaluation of the Primary Motor Cortex in Patients with Amyotrophic Lateral Sclerosis at 3T | * | - | - | * | * | - | - |
| Duning, 2011 | G-CSF prevents the progression of structural disintegration of white matter tracts in amyotrophic lateral sclerosis: a pilot trial | * | * | - | * | * | * | * |
| El Mendili, 2014 | Multi-parametric spinal cord MRI as potential progression marker in amyotrophic lateral sclerosis | - | - | N/A | N/A | N/A | - | - |
| Ellis, 2001 | Volumetric analysis reveals corticospinal tract degeneration and extramotor involvement in ALS | * | - | - | * | * | - | * |
| Endo, 2018 | Low signal intensity in motor cortex on susceptibility-weighted MR imaging is correlated with clinical signs of amyotrophic lateral sclerosis: a pilot study | * | - | - | * | * | - | * |
| Evans, 2015 | Impaired cognitive flexibility in amyotrophic lateral sclerosis | * | - | - | * | - | - | - |
| Fabes, 2017 | Quantitative FLAIR MRI in Amyotrophic Lateral Sclerosis | * | - | * | * | * | * | * |
| Feron, 2018 | Extrapyramidal deficits in ALS: a combined biomechanical and neuroimaging study | * | * | * | * | * | - | * |
| Ferraro, 2017 | Multimodal structural MRI in the diagnosis of motor neuron diseases | * | * | * | * | * | * | * |
| Ferraro, 2018 | Perfusion alterations converge with patterns of pathological spread in transactive response DNA-binding protein 43 proteinopathies | * | * | * | * | * | - | * |
| Finegan, 2019 | The clinical and radiological profile of primary lateral sclerosis: a population-based study | * | * | - | * | * | - | * |
| Finegan, 2020 | Widespread subcortical grey matter degeneration in primary lateral sclerosis: a multimodal imaging study with genetic profiling | * | * | - | * | * | - | * |
| Floeter, 2016 | Longitudinal imaging in C9orf72 mutation carriers: Relationship to phenotype | * | - | - | * | * | - | * |
| Frank, 1997 | Relation of neuropsychological and magnetic resonance findings in amyotrophic lateral sclerosis: evidence for subgroups | - | * | - | * | * | * | * |
| Geevasinga, 2017 | Brain functional connectome abnormalities in amyotrophic lateral sclerosis are associated with disability and cortical hyperexcitability | * | * | - | * | * | - | * |
| Goodin, 1988 | Magnetic resonance imaging in amyotrophic lateral sclerosis | - | - | N/A | N/A | - | * | N/A |
| Graham, 2004 | Diffusion tensor imaging for the assessment of upper motor neuron integrity in ALS | * | - | * | * | * | * | * |
| Grieve, 2015 | Potential structural and functional biomarkers of upper motor neuron dysfunction in ALS | * | * | - | * | * | - | * |
| Grossman, 2008 | Impaired action knowledge in amyotrophic lateral sclerosis | * | * | N/A | N/A | * | - | * |
| Gupta, 2014 | Accuracy of Conventional MRI in ALS | * | - | - | * | * | * | * |
| Hartung, 2014 | Voxel-based MRI intensitometry reveals extent of cerebral white matter pathology in amyotrophic lateral sclerosis | * | - | - | * | * | * | * |
| Hecht, 2005 | Cortical T2 signal shortening in amyotrophic lateral sclerosis is not due to iron deposits | * | * | N/A | N/A | N/A | - | * |
| Hecht, 2001 | MRI-FLAIR images of the head show corticospinal tract alterations in ALS patients more frequently than T2-, T1- and proton-density-weighted images | * | - | - | * | - | * | * |
| Hecht, 2002 | Hyperintense and hypointense MRI signals of the precentral gyrus and corticospinal tract in ALS: a follow-up examination including FLAIR images | * | - | N/A | N/A | N/A | * | - |
| Hofmann, 1998 | The corticospinal tract in amyotrophic lateral sclerosis: an MRI study | * | - | - | - | - | * | * |
| Ignjatovic, 2013 | Brain iron MRI: a biomarker for amyotrophic lateral sclerosis | * | - | - | * | * | * | * |
| Irwin, 2013 | Cognitive decline and reduced survival in C9orf72 expansion frontotemporal degeneration and amyotrophic lateral sclerosis | * | - | - | * | * | - | - |
| Ishikawa, 1993 | Signal loss in the motor cortex on magnetic resonance images in amyotrophic lateral sclerosis | - | - | - | * | * | * | * |
| Iwasaki, 1989 | Central nervous system magnetic resonance imaging findings in amyotrophic lateral sclerosis | - | - | N/A | N/A | N/A | - | N/A |
| Iwasaki, 1991 | MRI in patients with amyotrophic lateral sclerosis: correlation with clinical features |  |  |  |  |  |  |  |
| Jin, 2019 | Dominant Heterogeneity of Upper and Lower Motor Neuron Degeneration to Motor Manifestation of Involved Region in Amyotrophic Lateral Sclerosis | * | - | * | * | * | * | * |
| Jin, 2016 | Hyperintensity of the corticospinal tract on FLAIR: A simple and sensitive objective upper motor neuron degeneration marker in clinically verified amyotrophic lateral sclerosis | * | - | * | * | * | * | * |
| Josephs, 2013 | Corticospinal tract degeneration associated with TDP-43 type C pathology and semantic dementia | - | - | N/A | N/A | * | * | * |
| Kamminga, 2016 | Syntactic comprehension deficits across the FTD-ALS continuum | * | - | * | * | * | - | * |
| Kassubek, 2005 | Global brain atrophy and corticospinal tract alterations in ALS, as investigated by voxel-based morphometry of 3-D MRI | * | - | - | * | * | * | * |
| Kato, 1993 | Involvement of the frontotemporal lobe and limbic system in amyotrophic lateral sclerosis: as assessed by serial computed tomography and magnetic resonance imaging | - | - | N/A | N/A | N/A | - | - |
| Keller, 2011 | Quantitative brain MR imaging in amyotrophic lateral sclerosis | * | - | - | * | * | * | * |
| Kiernan, 1994 | Frontal lobe atrophy in motor neuron diseases | * | - | - | * | * | - | * |
| Kim, 2017 | Relationship between Clinical Parameters and Brain Structure in Sporadic Amyotrophic Lateral Sclerosis Patients According to Onset Type: A Voxel-Based Morphometric Study | * | - | - | * | * | - | * |
| Kim, 2017 | Structural explanation of poor prognosis of amyotrophic lateral sclerosis in the non-demented state | * | - | - | * | * | - | * |
| Koike, 2015 | Apparent diffusion coefficients distinguish amyotrophic lateral sclerosis from cervical spondylotic myelopathy | * | - | - | * | * | - | * |
| Konno, 2013 | Japanese amyotrophic lateral sclerosis patients with GGGGCC hexanucleotide repeat expansion in C9ORF72 |  |  |  |  |  |  |  |
| Kono, 2014 | Clinical characteristics associated with corticospinal tract hyperintensity on magnetic resonance imaging in patients with amyotrophic lateral sclerosis | * | * | N/A | N/A | N/A | - | N/A |
| Kuipers-Upmeijer, 2001 | Primary lateral sclerosis: clinical, neurophysiological, and magnetic resonance findings | * | - | N/A | N/A | N/A | * | - |
| Kwan, 2012 | Iron accumulation in deep cortical layers accounts for MRI signal abnormalities in ALS: correlating 7 tesla MRI and pathology | * | * | * | * | * | * | * |
| Kwan, 2012 | Structural imaging differences and longitudinal changes in primary lateral sclerosis and amyotrophic lateral sclerosis | * | - | - | * | * | - | * |
| Le Ber, 2008 | Phenotype variability in progranulin mutation carriers: a clinical, neuropsychological, imaging and genetic study | * | * | N/A | N/A | * | * | * |
| Lee, 2017 | Quantitative susceptibility mapping of the motor cortex: a comparison of susceptibility among patients with amyotrophic lateral sclerosis, cerebrovascular disease, and healthy controls | * | * | - | * | * | * | * |
| Lee, 2014 | Altered network connectivity in frontotemporal dementia with C9orf72 hexanucleotide repeat expansion | * | * | - | * | * | - | * |
| Lee, 2017 | Network degeneration and dysfunction in presymptomatic C9ORF72 expansion carriers | - | - | - | * | * | - | * |
| Leslie, 2015 | Semantic deficits in amyotrophic lateral sclerosis | * | - | * | * | * | - | * |
| Libon, 2012 | Deficits in concept formation in amyotrophic lateral sclerosis | * | - | N/A | N/A | * | - | * |
| Lillo, 2012 | Grey and white matter changes across the amyotrophic lateral sclerosis-frontotemporal dementia continuum | * | - | - | * | * | - | * |
| Luis, 1990 | Magnetic resonance imaging in motor neuron disease | - | - | - | * | * | - | - |
| Machts, 2018 | Prefrontal cortical thickness in motor neuron disease | * | - | - | * | * | - | * |
| Machts, 2015 | Basal ganglia pathology in ALS is associated with neuropsychological deficits | * | - | - | * | * | - | * |
| Machts, 2018 | Global Hippocampal Volume Reductions and Local CA1 Shape Deformations in Amyotrophic Lateral Sclerosis | * | - | - | * | * | - | * |
| Mahoney, 2012 | Frontotemporal dementia with the C9ORF72 hexanucleotide repeat expansion: clinical, neuroanatomical and neuropathological features | - | - | N/A | N/A | * | - | * |
| Mahoney, 2012 | Longitudinal neuroimaging and neuropsychological profiles of frontotemporal dementia with C9ORF72 expansions | - | - | N/A | N/A | * | - | * |
| McCluskey, 2014 | ALS-Plus syndrome: non-pyramidal features in a large ALS cohort | * | * | - | * | * | - | * |
| McMillan, 2015 | C9orf72 promoter hypermethylation is neuroprotective: Neuroimaging and neuropathologic evidence | * | - | N/A | N/A | * | - | * |
| Menke, 2014 | Widespread grey matter pathology dominates the longitudinal cerebral MRI and clinical landscape of amyotrophic lateral sclerosis | * | - | - | * | * | - | * |
| Menke, 2018 | The two-year progression of structural and functional cerebral MRI in amyotrophic lateral sclerosis | * | - | - | * | * | - | * |
| Meoded, 2013 | Imaging findings associated with cognitive performance in primary lateral sclerosis and amyotrophic lateral sclerosis | * | - | - | * | * | - | * |
| Mezzapesa, 2007 | Whole-brain and regional brain atrophy in amyotrophic lateral sclerosis | * | - | - | * | * | * | * |
| Mezzapesa, 2013 | Cortical thinning and clinical heterogeneity in amyotrophic lateral sclerosis | * | - | - | * | * | * | * |
| Minnerop, 2009 | In vivo voxel-based relaxometry in amyotrophic lateral sclerosis | * | - | * | * | * | * | * |
| Mioshi, 2013 | Cortical atrophy in ALS is critically associated with neuropsychiatric and cognitive changes | * | - | - | * | * | - | * |
| Miwa, 2003 | T2-low signal intensity in the cortex in multiple system atrophy | * | - | - | * | * | * | * |
| Mori, 2007 | Symmetric temporal abnormalities on MR imaging in amyotrophic lateral sclerosis with dementia | * | * | N/A | N/A | N/A | - | * |
| Muller, 2020 | Focal alterations of the callosal area III in primary lateral sclerosis: An MRI planimetry and texture analysis | * | - | - | * | * | - | * |
| Muller, 2011 | Complementary image analysis of diffusion tensor imaging and 3-dimensional t1-weighted imaging: white matter analysis in amyotrophic lateral sclerosis | - | - | - | * | * | - | * |
| Murphy, 2007 | Continuum of frontal lobe impairment in amyotrophic lateral sclerosis | * | * | N/A | N/A | * | - | * |
| Nasseroleslami, 2019 | Characteristic Increases in EEG Connectivity Correlate With Changes of Structural MRI in Amyotrophic Lateral Sclerosis |  |  |  |  |  |  |  |
| Ngai, 2007 | Hyperintensity of the precentral gyral subcortical white matter and hypointensity of the precentral gyrus on fluid-attenuated inversion recovery: variation with age and implications for the diagnosis of amyotrophic lateral sclerosis | * | - | * | * | * |  |  |
| Oba, 1993 | Amyotrophic lateral sclerosis: T2 shortening in motor cortex at MR imaging | * | - | - | - | * | * | * |
| Obusez, 2018 | 7T MR of intracranial pathology: Preliminary observations and comparisons to 3T and 1.5T | - | - | N/A | N/A | N/A | * | * |
| Omer, 2017 | Neuroimaging patterns along the ALS-FTD spectrum: a multiparametric imaging study | * | - | - | * | * | - | * |
| Papma, 2017 | Cognition and gray and white matter characteristics of presymptomatic C9orf72 repeat expansion | - | - | * | * | * | - | * |
| Paquin, 2018 | Spinal Cord Gray Matter Atrophy in Amyotrophic Lateral Sclerosis | * | - | - | * | * | - | * |
| Peretti-Viton, 1999 | MRI of the intracranial corticospinal tracts in amyotrophic and primary lateral sclerosis | * | - | - | - | * | - | * |
| Piaggio, 2018 | Cord cross-sectional area at foramen magnum as a correlate of disability in amyotrophic lateral sclerosis | * | - | * | * | * | * | * |
| Pinkhardt, 2006 | Amygdala size in amyotrophic lateral sclerosis without dementia: an in vivo study using MRI volumetry | * | - | - | * | * | - | * |
| Pinto, 2019 | O'Sullivan-McLeod syndrome: Unmasking a rare atypical motor neuron disease | - | - | N/A | N/A | N/A | - | * |
| Placek, 2019 | UNC13A polymorphism contributes to frontotemporal disease in sporadic amyotrophic lateral sclerosis | * | - | * | * | * | - | * |
| Prell, 2015 | Susceptibility-weighted imaging provides insight into white matter damage in amyotrophic lateral sclerosis | * | - | - | * | * | - | * |
| Prell, 2014 | Transcranial brainstem sonography as a diagnostic tool for amyotrophic lateral sclerosis | * | - | - | * | * | - | - |
| Protogerou, 2011 | T2 FLAIR Increased Signal Intensity at the Posterior Limb of the Internal Capsule: Clinical Significance in ALS Patients | * | * | - | - | * | * | * |
| Qin, 2018 | Region-specific atrophy of precentral gyrus in patients with amyotrophic lateral sclerosis | * | - | - | * | * | * | * |
| Qiu, 2019 | Precentral degeneration and cerebellar compensation in amyotrophic lateral sclerosis: A multimodal MRI analysis | * | - | - | * | * | - | * |
| Querin, 2019 | Presymptomatic spinal cord pathology in c9orf72 mutation carriers: A longitudinal neuroimaging study | - | - | N/A | N/A | * | - | * |
| Querin, 2017 | Spinal cord multi-parametric magnetic resonance imaging for survival prediction in amyotrophic lateral sclerosis | * | - | N/A | N/A | N/A | * | * |
| Raaphorst, 2015 | Prose memory impairment in amyotrophic lateral sclerosis patients is related to hippocampus volume | * | - | * | - | * | * | * |
| Radakovic, 2018 | Frontostriatal grey matter atrophy in amyotrophic lateral sclerosis A visual rating study | * | - | * | - | * | * | * |
| Rajagopalan, 2014 | Distinct patterns of cortical atrophy in ALS patients with or without dementia: an MRI VBM study | * | * | - | - | - | - | * |
| Rajagopalan, 2015 | Brain Parenchymal Fraction: A Relatively Simple MRI Measure to Clinically Distinguish ALS Phenotypes | * | - | * | - | - | * | * |
| Rajagopalan, 2015 | Comparing brain structural MRI and metabolic FDG-PET changes in patients with ALS-FTD: 'the chicken or the egg?' question | - | - | * | * | - | - | * |
| Rajagopalan, 2015 | Disparate voxel based morphometry (VBM) results between SPM and FSL softwares in ALS patients with frontotemporal dementia: which VBM results to consider? | - | - | - | * | - | - | * |
| Rajagopalan, 2014 | Do preprocessing algorithms and statistical models influence voxel-based morphometry (VBM) results in amyotrophic lateral sclerosis patients? A systematic comparison of popular VBM analytical methods | - | - | N/A | N/A | N/A | - | * |
| Ramanathan, 2018 | Demographics and clinical characteristics of primary lateral sclerosis: case series and a review of literature | - | * | N/A | N/A | N/A | - | * |
| Roeben, 2019 | The motor band sign in ALS: presentations and frequencies in a consecutive series of ALS patients | * | * | N/A | N/A | N/A | - | * |
| Sarchielli, 2001 | Magnetic resonance imaging and 1H-magnetic resonance spectroscopy in amyotrophic lateral sclerosis | * | - | N/A | N/A | N/A | - | * |
| Sasaki, 1999 | Atypical form of amyotrophic lateral sclerosis | * | - | N/A | N/A | N/A | - | * |
| Schonecker, 2018 | Atrophy in the Thalamus But Not Cerebellum Is Specific for C9orf72 FTD and ALS Patients - An Atlas-Based Volumetric MRI Study | - | - | * | * | * | - | * |
| Schuster, 2014 | Cortical thinning and its relation to cognition in amyotrophic lateral sclerosis | * | - | * | * | * | - | * |
| Schuster, 2013 | Focal thinning of the motor cortex mirrors clinical features of amyotrophic lateral sclerosis and their phenotypes: a neuroimaging study | * | * | * | * | * | * | * |
| Schuster, 2014 | Longitudinal course of cortical thickness decline in amyotrophic lateral sclerosis | * | - | * | * | * | - | * |
| Schweitzer, 2015 | Quantitative susceptibility mapping of the motor cortex in amyotrophic lateral sclerosis and primary lateral sclerosis | * | * | * | * | * | * | * |
| Senda, 2017 | Structural MRI correlates of amyotrophic lateral sclerosis progression | * | - | * | * | * | - | * |
| Senda, 2011 | Progressive and widespread brain damage in ALS: MRI voxel-based morphometry and diffusion tensor imaging study | * | * | * | * | * | - | * |
| Sha, 2012 | Frontotemporal dementia due to C9ORF72 mutations: clinical and imaging features | - | * | * | * | * | * | * |
| Shellikeri, 2019 | Speech network regional involvement in bulbar ALS: a multimodal structural MRI study | * | - | - | * | * | - | * |
| Shen, 2018 | Brain Structural and Perfusion Signature of Amyotrophic Lateral Sclerosis With Varying Levels of Cognitive Deficit | * | - | - | * | * | - | * |
| Shen, 2018 | Monitoring Value of Multimodal Magnetic Resonance Imaging in Disease Progression of Amyotrophic Lateral Sclerosis: A Prospective Observational Study | - | - | N/A | N/A | N/A | - | * |
| Shindo, 2014 | Neuropsychological study of amyotrophic lateral sclerosis and parkinsonism-dementia complex in Kii peninsula, Japan | * | - | - | * | - | * | * |
| Steinbach, 2020 | Applying the D50 disease progression model to gray and white matter pathology in amyotrophic lateral sclerosis | * | * | - | * | * | - | * |
| Takeda, 2007 | Memory deficits in amyotrophic lateral sclerosis patients with dementia and degeneration of the perforant pathway A clinicopathological study | * | - | N/A | N/A | N/A | - | * |
| Tavazzi, 2015 | Grey matter damage in progressive multiple sclerosis versus amyotrophic lateral sclerosis: a voxel-based morphometry MRI study | * | * | - | * | - | - | - |
| Terada, 2016 | Correlation of frontal atrophy with behavioral changes in amyotrophic lateral sclerosis | * | - | - | * | * | - | * |
| Thivard, 2007 | Diffusion tensor imaging and voxel based morphometry study in amyotrophic lateral sclerosis: relationships with motor disability | * | - | - | * | * | - | * |
| Thorns, 2013 | Extent of cortical involvement in amyotrophic lateral sclerosis--an analysis based on cortical thickness | * | - | - | * | * | * | * |
| Thorpe, 1996 | Brain and spinal cord MRI in motor neuron disease | - | - | - | * | - | * | * |
| Trojsi, 2020 | Frontotemporal degeneration in amyotrophic lateral sclerosis (ALS): a longitudinal MRI one-year study | * | - | * | * | * | - | * |
| Turner, 2007 | Volumetric cortical loss in sporadic and familial amyotrophic lateral sclerosis | * | - | - | * | * | - | * |
| Udaka, 1992 | MRI and SPECT findings in amyotrophic lateral sclerosis. Demonstration of upper motor neurone involvement by clinical neuroimaging | * | - | N/A | N/A | N/A | - | * |
| Van Mossevelde, 2016 | Clinical features of TBK1 carriers compared with C9orf72, GRN and non-mutation carriers in a Belgian cohort | * | - | N/A | N/A | N/A | - | * |
| Vazquez-Costa, 2019 | The width of the third ventricle associates with cognition and behaviour in motor neuron disease | * | - | - | * | - | - | * |
| Vazquez-Costa, 2018 | Brain signal intensity changes as biomarkers in amyotrophic lateral sclerosis | * | * | * | - | * | * | * |
| Verstraete, 2010 | No evidence of microbleeds in ALS patients at 7 Tesla MRI | * | * | * | * | * | * | * |
| Verstraete, 2012 | Structural MRI reveals cortical thinning in amyotrophic lateral sclerosis | * | * | * | * | * | - | * |
| Vibha, 2015 | Clinical profile of Monomelic Amyotrophy (MMA) and role of persistent viral infection | - | * | * | * | * | - | * |
| Vincenti, 2019 | Primary progressive aphasia and the FTD-MND spectrum disorders: clinical, pathological, and neuroimaging correlates | - | * | N/A | N/A | N/A | - | * |
| Walhout, 2015 | Cortical thickness in ALS: towards a marker for upper motor neuron involvement | * | - | - | * | * | - | * |
| Waragai, 1997 | MRI and clinical features in amyotrophic lateral sclerosis | - | - | - | * | - | * | * |
| Waragai, 1997 | High signal intensity on T1 weighted MRI of the anterolateral column of the spinal cord in amyotrophic lateral sclerosis | - | - | - | - | * | * | * |
| Welton, 2019 | Diffusion kurtosis and quantitative susceptibility mapping MRI are sensitive to structural abnormalities in amyotrophic lateral sclerosis | * | - | - | * | * | - | * |
| Westeneng, 2015 | Subcortical structures in amyotrophic lateral sclerosis | * | - | N/A | N/A | N/A | - | * |
| Westeneng, 2016 | Widespread structural brain involvement in ALS is not limited to the C9orf72 repeat expansion | * | - | - | * | * | - | * |
| Whitwell, 2006 | Patterns of atrophy in pathologically confirmed FTLD with and without motor neuron degeneration | - | - | - | * | * | - | * |
| Wirth, 2019 | Value of fluid-attenuated inversion recovery MRI data analyzed by the lesion segmentation toolbox in amyotrophic lateral sclerosis | - | - | - | * | * | - | * |
| Wirth, 2018 | Combinatory Biomarker Use of Cortical Thickness, MUNIX, and ALSFRS-R at Baseline and in Longitudinal Courses of Individual Patients With Amyotrophic Lateral Sclerosis | - | - | - | * | * | - | * |
| Wu, 2006 | Comparison of diffusion-weighted MR imaging and T2-weighted MR imaging in patients with amyotrophic lateral sclerosis | * | - | - | * | - | * | * |
| Yagishita, 1994 | Location of the corticospinal tract in the internal capsule at MR imaging | - | - | N/A | N/A | N/A | - | - |
| York, 2014 | Action verb comprehension in amyotrophic lateral sclerosis and Parkinson's disease | * | - | - | * | * | - | * |
| Yu, 2014 | Increased iron level in motor cortex of amyotrophic lateral sclerosis patients: an in vivo MR study | * | * | * | * | * | - | * |
| Yunusova, 2019 | Frontal Anatomical Correlates of Cognitive and Speech Motor Deficits in Amyotrophic Lateral Sclerosis | * | - | N/A | N/A | N/A | - | * |
| Zhang, 2014 | Regional alterations in cortical thickness and white matter integrity in amyotrophic lateral sclerosis | * | - | * | * | - | - | * |
| Zhang, 2003 | The diagnostic utility of FLAIR imaging in clinically verified amyotrophic lateral sclerosis | * | - | - | * | - | * | - |
| Zhang, 2014 | Side of limb-onset predicts laterality of gray matter loss in amyotrophic lateral sclerosis | * | * | - | * | * | - | * |
| Zhang, 2017 | Occipital cortical gyrification reductions associate with decreased functional connectivity in amyotrophic lateral sclerosis | * | * | * | * | - | - | * |
| Zhang, 2019 | Abnormal topological organization of structural covariance networks in amyotrophic lateral sclerosis | * | * | * | * | * | - | * |
| Zhu, 2015 | ALFF Value in Right Parahippocampal Gyrus Acts as a Potential Marker Monitoring Amyotrophic Lateral Sclerosis Progression: a Neuropsychological, Voxel-Based Morphometry, and Resting-State Functional MRI Study | * | * | - | * | * | - | * |

**Supplementary Table 3**: Summary of studies using magnetic resonance imaging (MRI) in human studies of motor neuron disease. HC, healthy controls, i.e., does the study comprise a healthy control group (0, no; 1, yes). L, longitudinal study (0, no; 1, yes).

*Glossary: ADC, apparent diffusion coefficient; ALS, amyotrophic lateral sclerosis; DTI, diffusion tensor imaging; DWI, diffusion-weighted imaging; FTD, frontotemporal dementia (bv, behavioral variant); FTLD, frontotemporal lobar degeneration; LMN, lower motor neuron; MND, motor neuron disease; MTR, magnetization transfer ratio; PLS; primary lateral sclerosis; PPA, primary progressive aphasia; T1w, T1-weighted; T2w, T2-weighted; T2*w, T2*-weighted; UMN, upper motor neuron.*

| **Author, year** | **Title** | **Disease** | **Main findings** | **HC** | **L** | **Method** | **Imaging feature** |
| --- | --- | --- | --- | --- | --- | --- | --- |
| Abdulla, 2014 | Hippocampal degeneration in patients with amyotrophic lateral sclerosis | ALS | Hippocampus volume smaller in ALS | 1 | 0 | Manual segmentation (MultiTracer) | Hippocampus volume |
| Abe, 1997 | Degeneration of the pyramidal tracts in patients with amyotrophic lateral sclerosis. A premortem and postmortem magnetic resonance imaging study | ALS | Internal capsule CST hyperintensities | 1 | 0 | Visual rating | T2 hyperintensity CST (not on PD!) |
| Abe, 1993 | Single-photon emission computed tomographic investigation of patients with motor neuron disease | MND | CST hyperintensity in ALS, but also in some controls. | 0 | 0 | Visual rating | T2 hyperintensity CST |
| Abrahams, 2001 | Executive dysfunction in amyotrophic lateral sclerosis (ALS); a functional and structural MRI study | ALS | Lower white matter volumes (frontal and temporal lobes) in ALS compared to controls. But no diff in grey matter volumes ALS vs controls | 1 | 0 | Visual rating | White/gray matter volume |
| Abrahams, 2005 | Frontotemporal white matter changes in amyotrophic lateral sclerosis | ALS (with or without cognitive impairment) | Lower white matter volumes in ALS compared to controls in motor and non-motor regions. No difference in gray matter volumes | 1 | 0 | Automated VBM | White/gray matter volume |
| Acosta-Cabronero, 2018 | Quantitative Susceptibility MRI to Detect Brain Iron in Amyotrophic Lateral Sclerosis | ALS | No difference in subcortical and cortical volumes in ALS versus controls | 1 | 0 | Freesurfer | White/gray matter volume, QSM |
| Adachi, 2015 | Usefulness of SWI for the Detection of Iron in the Motor Cortex in Amyotrophic Lateral Sclerosis | ALS | Motor band sign: SWI was the most sensitive sequence to detect low signal of motor cortex. T2*-weighted image contrast was less sensitive than conventional spin-echo T2-weighted images | 1 | 0 | Visual rating | Motor band sign |
| Agosta, 2016 | Structural brain correlates of cognitive and behavioral impairment in MND | Classical ALS, upper motor neuron ALS, lower motor neuron ALS | Thinning of bilateral precentral gyrus, insular and cingulate cortices, and frontotemporal regions in ALS versus controls | 1 | 0 | Freesurfer | White/gray matter volume |
| Agosta, 2017 | Structural and functional brain signatures of C9orf72 in motor neuron disease | ALS (C9orf72+) | All ALS patients (sporadic or C9orf72) showed motor, frontal, and temporoparietal cortical thinning as well as motor and extramotor white matter volume loss versus controls, independent of genotype and presence of cognitive impairment. C9orf72 patients showed cerebellar and thalamic atrophy compared to sporadic ALS cases. | 1 | 0 | Freesurfer | White/gray matter volume |
| Agosta, 2015 | MRI signatures of the frontotemporal lobar degeneration continuum | MND, behavioral variant of frontotemporal dementia, nonfluent primary progressive aphasia | All clinical FTLD continuum phenotypes (except ALS with pure motor phenotype) showed more pronounced white than gray matter volume loss. | 1 | 0 | Diffeomorphic Anatomical Registration Exponentiated Lie Algebra (DARTEL), SPM | White/gray matter volume |
| Agosta, 2009 | Longitudinal assessment of grey matter contraction in amyotrophic lateral sclerosis: A tensor based morphometry study | ALS | ALS patients mainly showed progression of gray matter atrophy in left premotor cortex and right basal ganglia | 1 | 1 | SPM2 | White/gray matter volume |
| Agosta, 2007 | Voxel-based morphometry study of brain volumetry and diffusivity in amyotrophic lateral sclerosis patients with mild disability | ALS | ALS patients had clusters of locally reduced gray matter density in the right premotor cortex, left inferior frontal gyrus and superior temporal gyrus bilaterally. | 1 | 0 | SPM2 | White/gray matter volume |
| Agosta, 2019 | Survival prediction models in motor neuron disease | ALS, upper motor neuron ALS, lower motor neuron ALS | No difference in over 90 cortical or subcortical regional brain volumes in ALS compared to control. | 1 | 0 | VBM | White/gray matter volume |
| Agosta , 2012 | The cortical signature of amyotrophic lateral sclerosis | ALS | ALS patients show widespread cortical thinning compared to control subjects (primary motor, prefrontal and ventral frontal cortices, cingulate gyrus, insula, superior and inferior temporal and parietal regions, and medial and lateral occipital areas) | 1 | 0 | Freesurfer | White/gray matter volume |
| Alruwaili, 2018 | A combined tract-based spatial statistics and voxel-based morphometry study of the first MRI scan after diagnosis of amyotrophic lateral sclerosis with subgroup analysis | ALS | Lower left middle frontal gyrus volumes in ALS versus controls. | 1 | 0 | Visual rating | White/gray matter volume |
| Ambikairajah, 2014 | A visual MRI atrophy rating scale for the amyotrophic lateral sclerosis-frontotemporal dementia continuum | ALS, ALS-FTD, bvFTD | Volumes of 4 regions was visually assessed (orbitofrontal cortex, anterior temporal pole, anterior cingulate, and motor cortex) in different ALS phenotypes. bvFTD phenotypes showed the highest levels of atrophy across all regions, while ALS patients had the lowest atrophy scores. ALS only differed from healthy controls on orbitofrontal atrophy ratings but none of the other ratings. | 1 | 0 | FSL VBM | White/gray matter volume |
| Andreadou, 1998 | Subcortical frontal lesions on MRI in patients with motor neurone disease | MND | 10/21 patients with white matter T2w and PDw hyperintensities. | 1 | 0 | Visual rating | T2 hyperintensity CST (and PD!) |
| Ash, 2014 | Narrative discourse deficits in amyotrophic lateral sclerosis | ALS (nonaphasic) | ALS patients showed gray matter atrophy in ventral and dorsal prefrontal regions compared to healthy controls. | 1 | 0 | FSL VBM | White/gray matter volume |
| Bae, 2016 | Dissociation of Structural and Functional Integrities of the Motor System in Amyotrophic Lateral Sclerosis and Behavioral-Variant Frontotemporal Dementia | ALS, bvFTD | ALS patients showed lower gray matter volumes in the motor cortex, cerebellum and thalamus compared to controls. bvFTD had lower gray matter volumes in the cerebellum. | 1 | 0 | Manual segmentation | White/gray matter volume |
| Basak, 2002 | Magnetic resonance imaging in amyotrophic lateral sclerosis | ALS | ALS patients had a higher prevalence of T2w motor cortex hypointensity compared to non-healthy controls (unclear diseases), yet there was no difference between ALS and controls in CST hyperintensity on T2/PDw image contrast. | 1 | 0 | Visual rating | T2/PD hyperintensity CST, motor band sign |
| Bede, 2013 | The Neuroimaging Signature of the C9orf72 Hexanucleotide Repeat in Amyotrophic Lateral Sclerosis - A Multimodal MRI Study | ALS (C9orf72+ or -) | C9orf72+ patients had lower cortical volumes in several cortical regions: the left fusiform, left supramarginal, leftsuperior temporal gyrus, left orbitofrontal cortex, left lateral occipital cortex, and left posterior cingulate. In contrast to C9orf72+ ALS patients, there was no difference in cortical thickness between C9orf72- patients and healthy controls. | 1 | 0 | Not reported | White/gray matter volume |
| Bede, 2013 | Grey Matter Correlates of Clinical Variables in Amyotrophic Lateral Sclerosis - A Neuroimaging Study of ALS Motor Phenotype Heterogeneity and Cortical Focality | ALS | Bulbar onset ALS patients showed lower volumes in the bulbar segment of the motor homunculus. Limb onset ALS patients showed cortical volume loss in the limb segment of the motor cortex. | 1 | 0 | FSL VBM | White/gray matter volume |
| Bede, 2013 | Basal ganglia involvement in amyotrophic lateral sclerosis | ALS (C9orf72+ or -) | C9orf72- patients showed volume reductions in the left caudate nucleus, left hippocampus, and right accumbens nucleus compared with healthy controls. C9orf72+ patients showed volume reductions in the bilateral thalami, left caudate, left putamen and bilateral hippocampi. | 1 | 0 | FSL VBM | White/gray matter volume |
| Bede, 2018 | Longitudinal structural changes in ALS: a three time-point imaging study of white and gray matter degeneration | ALS (C9orf72-) | During the 8 month follow-up, gray matter degeneration involved the bilateral frontal, temporal, and parietal lobes as well as the cerebellum. The bilateral precentral gyrus showed particular volume loss. | 0 | 1 | Freesurfer | White/gray matter volume |
| Bede, 2018 | Connectivity-based characterisation of subcortical grey matter pathology in frontotemporal dementia and ALS: a multimodal neuroimaging study | bvFTD, PPA, ALS-FTD C9+, ALS-FD C9-, ALS | Non-fluent-variant PPA patients had volume reductions in the thalamus and putamen. Behavioral-variant FTD patients had nucleus accumbens and hippocampal volume loss. Semantic-variant PPA patients only showed volumetric loss in the left hippocampus. | 1 | 0 | FSL VBM | White/gray matter volume |
| Bocchetta, 2018 | Thalamic atrophy in frontotemporal dementia — Not just a C9orf72 problem | FTD-MND | FTD-MND patients with lower thalamic volumes compared to healthy controls | 1 | 0 | SPM12 | White/gray matter volume |
| Bocchetta, 2020 | Thalamic nuclei in frontotemporal dementia: Mediodorsal nucleus involvement is universal but pulvinar atrophy is unique to C9orf72 | FTD-MND | Smaller thalamic nuclei in all FTD subgroups (mediodorsal and laterodorsal) | 1 | 0 | SPM12 | White/gray matter volume |
| Boll, 2019 | Is the Hypointensity in Motor Cortex the Hallmark of Amyotrophic Lateral Sclerosis? | ALS | 33/36 ALS patients with black ribbon sign (92%) on T2*w contrast. In 7 patients MRI follow-up at 18 months: no change in black ribbon sign. | 1 | 1 | Visual rating | Motor band sign |
| Branco, 2018 | Brain signature of mild stages of cognitive and behavioral impairment in amyotrophic lateral sclerosis | C9orf72-negative non-demented ALS patients | ALS patients with cognitive impairment had lower left thalamic and amygdala volumes compared to healthy controls | 1 | 0 | Freesurfer | White/gray matter volume |
| Buhour, 2017 | Voxel-based mapping of grey matter volume and glucose metabolism profiles in amyotrophic lateral sclerosis | ALS | ALS patients showed volume loss predominantely in the temporal pole, left hippocampus and right thalamus compared to healthy controls | 1 | 0 | SPM5 VBM | White/gray matter volume |
| Butman, 2007 | Decreased thickness of primary motor cortex in primary lateral sclerosis | PLS | Thinner motor cortex in PLS compared to healthy controls | 1 | 0 | Visual rating | White/gray matter volume |
| Canu, 2011 | The topography of brain microstructural damage in amyotrophic lateral sclerosis assessed using diffusion tensor MR imaging | ALS | 12/23 ALS patients (52%) and 5/12 healthy controls (35%) showed T2w-FLAIR hyperintensities in the CST. ALS patients showed lower volumes in the precentral gyrus bilaterally, right rolandic operculum and angular gyrus, and left middle frontal gyrus. White matter volume changes were not different between ALS and healthy controls after adjusting the analysis for microstructural details. | 1 | 0 | Diffeomorphic Anatomical Registration Exponentiated Lie Algebra (DARTEL), SPM | T2 hyperintensity CST and white/gray matter volume |
| Cardenas-Blanco, 2016 | Structural and diffusion imaging versus clinical assessment to monitor amyotrophic lateral sclerosis | ALS | Structural MRI did not show changes in cortical thickness or deep gray matter volume in ALS versus healthy controls over 3 scans within approximately 9 months. | 1 | 1 | Freesurfer | White/gray matter volume |
| Carella, 1995 | Magnetic resonance signal abnormalities along the pyramidal tracts in amyotrophic lateral sclerosis | ALS | 11/16 ALS patients (69%) showed signal abnormalities along the CST. | 0 | 0 | Visual rating | T2 hyperintensity CST |
| Cerami, 2014 | Emotional empathy in amyotrophic lateral sclerosis: a behavioural and voxel-based morphometry study | ALS | Reduced gray matter density in the anterior cingulate cortex and right inferior frontal gyrus in ALS compared to healthy controls, associated with defective emotional empathy attribution. | 1 | 0 | Diffeomorphic Anatomical Registration Exponentiated Lie Algebra (DARTEL), SPM | White/gray matter volume |
| Cervo, 2015 | The combined use of conventional MRI and MR spectroscopic imaging increases the diagnostic accuracy in amyotrophic lateral sclerosis | ALS | Precentral gyrus T2 signal intensity was lower in ALS patients compared to healthy controls. No difference in terms of T2-hyperintensity of the CST. | 1 | 0 | Manual ROI placement | T2 hyperintensity CST and precentral gyrus T2w hypointensity |
| Chang, 2005 | A voxel-based morphometry study of patterns of brain atrophy in ALS and ALS/FTLD | ALS, ALS-FTLD | Both ALS and ALS-FTLD had lower volumes in bilateral motor/premotor cortices, the left middle and inferior frontal gyri, the anterior portion of the superior frontal gyri, the superior temporal gyri, the temporal poles and left posterior thalamus. Most of the frontal brain regions showed lower volumes in the ALS/FTLD group compared to the ALS group. | 1 | 0 | FSL VBM | White/gray matter volume |
| Chapman, 2012 | Corpus callosum area in amyotrophic lateral sclerosis | ALS | No difference in corpus callosum volume between ALS and healthy controls. | 1 | 0 | Manual segmentation | Corpus callosum volume |
| Charil, 2009 | Structural and metabolic changes in the brain of patients with upper motor neuron disorders: a multiparametric MRI study | ALS, PLS | T2w and/or PDw image contrast was highly sensitive in detecting CST hyperintensities in advanced stage ALS (63%) and PLS (72%), but it was less sensitive in early stage ALS (17%). | 1 | 0 | Visual rating | T2/PD hyperintensity CST |
| Chen, 2018 | MR Imaging-based Estimation of Upper Motor Neuron Density in Patients with Amyotrophic Lateral Sclerosis: A Feasibility Study | ALS | 11 ALS patients with postmortem in situ imaging and correlative histopathology. In sporadic ALS only (n=6), a linear function of normalized right premotor cortex and white matter volumes was able to estimate motor neuron density. | 1 | 0 | Visual rating | White/gray matter volume |
| Chen, 2018 | Cortical Thinning Pattern of Bulbar- and Spinal-onset Amyotrophic Lateral Sclerosis: a Surface-based Morphometry Study | ALS (bulbar or spinal onset) | In ALS patients, cortical thinning was in the left precentral gyrus, left postcentral gyrus, right gyrus rectus and right medial precentral gyrus. The bulbar-onset ALS showed motor cortex thinning of left precentral gyrus and right supplementary motor cortex, and the spinal-onset ALS showed extra-motor cortex thining of left posterior insula and right gyrus rectus. | 1 | 0 | Diffeomorphic Anatomical Registration Exponentiated Lie Algebra (DARTEL), SPM | White/gray matter volume |
| Cheung, 1995 | Amyotrophic lateral sclerosis: correlation of clinical and MR imaging findings | ALS | ALS patients with T2w/PDw CST hyperintensities, mainly in the internal capsule. Low signal intensity in the motor cortex on T1w image contrast. | 0 | 0 | Visual rating | T2/PD hyperintensity CST and motorcortex hypointensity |
| Christidi, 2019 | Hippocampal pathology in amyotrophic lateral sclerosis: selective vulnerability of subfields and their associated projections | ALS | The cornu ammonis 2/3 subfield and the hippocampus-amygdala transition area were the most affected regions in ALS compared to Alzheimer's disease. | 1 | 0 | Freesurfer, FSL VBM | Hippocampus volume |
| Christidi, 2018 | Gray matter and white matter changes in non-demented amyotrophic lateral sclerosis patients with or without cognitive impairment: A combined voxel-based morphometry and tract-based spatial statistics whole-brain analysis | ALS motor, ALS plus | Compared to healthy controls, ALS motor patients showed decreased gray matter volume in frontal and cerebellar regions and increased gray matter volumes in right supplementary motor area. ALS-plus patients showed diffuse gray matter volume reduction in primary motor cortices bilaterally, frontotemporal areas, cerebellum and basal ganglia. | 1 | 0 | SPM8 VBM | White/gray matter volume |
| Christidi, 2018 | Motor and extra-motor gray matter integrity may underlie neurophysiologic parameters of motor function in amyotrophic lateral sclerosis: a combined voxel-based morphometry and transcranial stimulation study | ALS | Compared to healthy controls, ALS patients showed decreased gray matter density frontal, temporal, parietal/occipital and cerebellar regions. | 1 | 0 | SPM8 VBM | White/gray matter volume |
| Clark, 2018 | Loss of functional connectivity is an early imaging marker in primary lateral sclerosis | pre-PLS, PLS | PLS patients with longstanding disease had cortical thinning of the precentral gyrus. Pre-PLS patients had a subtle thinning of the right precentral gyrus. Follow-up scans in 8 pre-PLS patients up to 2 years later showed increased thinning of the precentral gyrus. | 1 | 1 | Freesurfer | White/gray matter volume |
| Cohen-Adad, 2013 | Involvement of spinal sensory pathway in ALS and specificity of cord atrophy to lower motor neuron degeneration | ALS (spinal onset) | Local spinal cord atrophy was associated with weakness in the corresponding muscle territory. | 1 | 0 | Semiautomated segmentation | Spinal cord atrophy |
| Consonni, 2019 | Cognitive Syndromes and C9orf72 Mutation Are Not Related to Cerebellar Degeneration in Amyotrophic Lateral Sclerosis | ALS (non-demented) | In ALS patients, cortical thinning was associated with decreased physical and cognitive performance. No cortical thinning or volume reduction in the cerebellum of ALS patients. | 1 | 0 | CERES automated cerebellum parcellation algorithm | White/gray matter volume |
| Consonni, 2019 | Cortical correlates of behavioural change in amyotrophic lateral sclerosis | ALS (non-demented) | In ALS, the thinning of the bilateral orbitofrontal cortex was associated with apathy, the right frontotemporal and cingular cortex with the disinhibited/hostile profile and the left precuneus cortex with dysexecutive behaviour. | 0 | 0 | Freesurfer | White/gray matter volume |
| Consonni, 2018 | Cortical markers of cognitive syndromes in amyotrophic lateral sclerosis | ALS (non-demented) | All ALS patients showed cortical thinning in a fronto-parietal network | 1 | 0 | Freesurfer | White/gray matter volume |
| Contarino, 2020 | Toward a marker of upper motor neuron impairment in amyotrophic lateral sclerosis: A fully automatic investigation of the magnetic susceptibility in the precentral cortex | ALS | Decreased cortical thickness in ALS compared to healthy controls | 1 | 0 | Freesurfer | White/gray matter volume |
| Coon, 2011 | Predicting survival in frontotemporal dementia with motor neuron disease | FTD-MND (dominant behavioral symptoms vs. dominant language symptoms) | In both clinical phenotypes, there was af frontal and temporal lobe volume reduction. There was some evidence for the behavioral type to show more frontal atrophy and the language type to have more left temporal atrophy. | 0 | 1 | SPM5 VBM | White/gray matter volume |
| Coon, 2012 | Right temporal variant frontotemporal dementia with motor neuron disease | FTD-MND | 3 FTD-MND patients with dominant right temporal lobe atrophy and right CST degeneration. | 0 | 0 | SPM5 VBM | White/gray matter volume |
| Cosottini, 2013 | Mapping cortical degeneration in ALS with magnetization transfer ratio and voxel-based morphometry | ALS | ALS showed gray matter volume loss in motor-related areas and extra-motor frontotemporal cortices. | 1 | 0 | FSL VBM | White/gray matter volume |
| Cosottini, 2016 | High-Resolution 7T MR Imaging of the Motor Cortex in Amyotrophic Lateral Sclerosis | ALS | MRI at 7T showed volume loss and signal hypointensity in the deep layers of the primary motor cortex with a diagnostic accuracy of 71%. Signal hypointensity of the deep layers of the primary motor cortex correlated with upper motor neuron disability and with disease progression. | 1 | 0 | Manual ROI placement | Signal hypointensity on motor cortex |
| Cosottini, 2012 | Structural and functional evaluation of cortical motor areas in Amyotrophic Lateral Sclerosis | ALS | A voxel-based morphometry approach showed that ALS patients have several clusters of reduced cortical gray matter compared to healthy controls, including reduced cortical gray matter, including the pre and postcentral gyri, the superior, middle and inferior frontal gyri, the supplementary motor area, the superior and inferior parietal cortices and the temporal lobe, bilaterally but more extensive on the right side. | 1 | 0 | FSL VBM | White/gray matter volume |
| Costagli, 2016 | Magnetic susceptibility in the deep layers of the primary motor cortex in Amyotrophic Lateral Sclerosis | ALS | In ALS patients, increases in magnetic susceptibility at 7T MRI co-localized with the T2* hypointensity observed in the middle and deep layers of the motor cortex. | 1 | 0 | Manual ROI placement | Motor band sign, QSM |
| Crespi, 2018 | Multimodal MRI quantification of the common neurostructural bases within the FTD-ALS continuum | bvFTD, ALS (non-demented) | Both clinical phenotypes showed similar macrostructural and microstructural damage compared with healthy controls, involving the right orbital and the bilateral anterior cingulate cortices, the corticospinal tract and corpus callosum. Gray and white matter volume loss was higher in orbitofrontal and frontomedial regions in patients with more severe executive and/or behavioral symptoms, and a higher degree of degeneration in the motor pathway in patients with more severe motor neuron disorders. | 1 | 0 | SPM8 VBM | White/gray matter volume |
| d'Ambrosio, 2014 | Frontotemporal cortical thinning in amyotrophic lateral sclerosis | ALS | ALS patients showed a significant cortical thinning in multiple motor and extramotor cortical areas when compared with healthy controls. | 1 | 0 | Freesurfer | White/gray matter volume |
| da Rocha, 1999 | Magnetic resonance findings in amyotrophic lateral sclerosis using a spin echo magnetization transfer sequence. Preliminary report | ALS | T2w CST hyperintensities in 5/5 patients. | 0 | 0 | Not reported | T2 hyperintensity CST |
| da Rocha, 2004 | Detection of corticospinal tract compromise in amyotrophic lateral sclerosis with brain MR imaging: relevance of the T1-weighted spin-echo magnetization transfer contrast sequence | ALS | T1w CST hypointensities in 20/25 ALS patients (80%). These corresponded to T2w-FLAIR hyperintensities. | 1 | 0 | Visual rating | T1 Hypointensity CST, T2 hyperintensity CST |
| de Albuguergue, 2016 | Multimodal Longitudinal MRI Study in Amyotrophic Lateral Sclerosis (ALS) | ALS | ALS patients did not show any region with progressive cortical thinning but there was a brainstem volumetric reduction for 6 months follow-up | 1 | 1 | SpineSeg | White/gray matter volume (incl. brain stem) |
| de Albuguergue, 2016 | MRI Texture Analysis Reveals Deep Gray Nuclei Damage in Amyotrophic Lateral Sclerosis | ALS | Higher parameter correlation in ALS patients in both thalami and in the right caudate nucleus compared to healthy controls. | 1 | 0 | Manual (Mazda) | White/gray matter volume |
| de Albuguergue, 2017 | Longitudinal evaluation of cerebral and spinal cord damage in Amyotrophic Lateral Sclerosis | ALS | There was no brain region with progressive cortical thinning during the 8 months follow-up period, but there was significant brainstem volumetric reduction. | 1 | 1 | Freesurfer | White/gray matter volume |
| De Marco, 2015 | Morphometric correlates of dysarthric deficit in amyotrophic lateral sclerosis | ALS | ALS patients showed a white matter volume loss located in peri-cortical motor/premotor fibres bilaterally, and in a large volume extending from the pons/midbrain to the cerebellum. | 1 | 0 | SPM VBM | White/gray matter volume |
| De Reuck, 2017 | Topographic distribution of brain iron deposition and small cerebrovascular lesions in amyotrophic lateral sclerosis and in frontotemporal lobar degeneration: a post-mortem 7.0-tesla magnetic resonance imaging study with neuropathological correlates | ALS, FTLD | The amount of Fe deposition in the deep brain structures and the number of small cerebrovascular lesions was determined in ALS and the subtypes of FTLD compared to control brains, with neuropathological correlates. A significant increase of Fe deposition was observed in the claustrum, caudate nucleus, globus pallidus, thalamus, and subthalamic nucleus of the FTLD-FUS and FTLD-TDP groups, while in the ALS one, the Fe increase was only observed in the caudate and the subthalamic nuclei. White matter changes were only significantly more severe in the FTLD compared to those in ALS and in controls brains. Cortical micro-bleeds were increased in the frontal and temporal lobes of FTLD as well as of ALS brains compared to controls. Cortical micro-infarcts were more common in the control compared to the ALS and FTLD groups. | 1 | 0 | N/A | Iron deposition, CMB, microinfarcts |
| De Reuck, 2014 | Iron deposits in post-mortem brains of patients with neurodegenerative and cerebrovascular diseases: a semi-quantitative 7.0 T magnetic resonance imaging study | ALS, FTLD | FTLD brains showed increased iron in the claustrum, caudate nucleus, and putamen and to a lesser degree in the globus pallidus, thalamus and subthalamic nucleus. In the other neurodegenerative diseases (i.e., Alzheimer's disease, Lewy body disease, progressive supranuclear palys), no iron accumulation was observed, except for a small increase in the caudate nucleus of AD brains. | 1 | 0 | N/A | Iron deposition |
| Devenney, 2014 | Frontotemporal dementia associated with the C9ORF72 mutation: a unique clinical profile | bvFTD (C9orf72+ or -), FTD-ALS | During the 5 year follow-up, C9orf72+ patients had more atrophy in the precuneus compared to C9orf72- patients. By contrast, the C9orf72- patients had more atrophy than controls in each of the 7 brain regions. Of note, the C9orf72+ patients showed an absence of significant atrophy of the orbitofrontal cortex, anterior cingulate, insula, and temporal pole. | 1 | 1 | Visual rating (Likert scale) | White/gray matter volume |
| Devenney, 2017 | The neural correlates and clinical characteristics of psychosis in the frontotemporal dementia continuum and the C9orf72 expansion | bvFTD | Psychotic symptoms in C9orf72+ patients correlated with atrophy in a cortical and subcortical networks that included discrete regions of the frontal, temporal, and occipital cortices, as well as the thalamus, striatum and cerebellum. | 1 | 0 | FSL VBM | White/gray matter volume |
| Devine, 2015 | Exposing asymmetric gray matter vulnerability in amyotrophic lateral sclerosis | ALS | ALS patients showed atrophy of the dominant (left) motor cortex hand area, irrespective of the side of first limb weakness. Asymmetric atrophy of the left somatosensory cortex and temporal gyri was only observed in ALS patients with right-sided onset of limb weakness. | 1 | 0 | FSL VBM | White/gray matter volume |
| Ding, 2011 | Value of quantitative analysis of routine clinical MRI sequences in ALS | ALS | In ALS patients, increased proton densitiy was observed in the CST, corpus callosum and white and grey matter. T2 elongation was found at the genu of corpus callosum and at the posterior limb of the internal capsule. Thus, proton densitiy appeared to be the most sensitive parameter for the detection of degenerative changes in the motor system and in extramotor brain regions. | 1 | 0 | Visual assessment and placement of ROIs | T2/PD hyperintensity CST, ADC |
| Donatelli, 2019 | MRI cortical feature of bulbar impairment in patients with amyotrophic lateral sclerosis | ALS | The marked hypointensity of the primary motor cortex was detected in 14/55 ALS patients (25%), including all patients with bulbar onset. These hypointensities were usually visible in both hemispheres. The magnetic susceptibility was significantly higher in patients with marked primary motor cortex hypointensity than in the other ALS patients. | 1 | 0 | Visual assessment of hypointensity in motor cortex | Motor band sign |
| Donatelli, 2018 | Semiautomated Evaluation of the Primary Motor Cortex in Patients with Amyotrophic Lateral Sclerosis at 3T | ALS | Study investigated the signal hypointensity-to-thickness ratio of the primary motor cortex as a radiologic marker of upper motor neuron involvement in ALS. The signal hypointensity-to-thickness ratio of the primary motor cortex was greater in ALS compared to healthy controls. The diagnostic accuracy of the signal hypointensity- to-thickness ratio was high at 3T AUC = 0.89) and even higher at 7T (AUC = 0.94). | 1 | 0 | Visual assessment compared to in-house-made software | Motor band sign, white/gray matter atrophy |
| Duning, 2011 | G-CSF prevents the progression of structural disintegration of white matter tracts in amyotrophic lateral sclerosis: a pilot trial | ALS | No differences in local gray matter volumes between ALS patients and healthy controls. There were no differences in brain volume measures between ALS groups and healthy controls. | 0 | 1 | SPM5, FSL VBM | White/gray matter volume |
| El Mendili, 2014 | Multi-parametric spinal cord MRI as potential progression marker in amyotrophic lateral sclerosis | ALS | Within 12 months of follow-up, ALS patients showed lower cross-sectional spinal cord area. | 0 | 1 | Manual placement of ROIs | White/gray matter volume |
| Ellis, 2001 | Volumetric analysis reveals corticospinal tract degeneration and extramotor involvement in ALS | ALS (limb or bulbar onset) | No differences in the total brain volumes of gray or white matter between three subject groups (ALS bulbar/limb onset, healthy controls). Comparing all ALS patients with the healthy controls showed localized volume loss in gray matter centered on Brodmann areas 8, 9, and 10 bilaterally. Comparing ALS patients with limb- and bulbar-onset showed loss in the white matter volume in the bulbar-onset group, extending bilaterally from the precentral gyrus into the internal capsule and brainstem along the CST. There was no volume loss in gray matter in the precentral gyri. | 1 | 0 | AFNI software, in-house-made software | White/gray matter volume |
| Endo, 2018 | Low signal intensity in motor cortex on susceptibility-weighted MR imaging is correlated with clinical signs of amyotrophic lateral sclerosis: a pilot study | ALS | ALS patients exhibited lower signal intensity in the precentral gyrus on susceptibility-weighted MR imaging compared with healthy controls. Interestingly, the extent of the susceptibility changes in the bilateral precentral gyri was correlated with upper motor neuron scores. | 1 | 0 | SPM12/MATLAB, MRIcro | Motor band sign |
| Evans, 2015 | Impaired cognitive flexibility in amyotrophic lateral sclerosis | ALS | Regression analyses showed impaired cognitive flexibility to gray matter atrophy in inferior frontal and insula regions. | 1 | 0 | Not reported | White/gray matter volume |
| Fabes, 2017 | Quantitative FLAIR MRI in Amyotrophic Lateral Sclerosis | ALS (flail arm or "classical" ALS), PLS | ALS patients had higher FLAIR intensity in the CST and corpus callosum compared to healthy controls. The cerebral peduncle intensity had the strongest subgroup classification. During a follow-up of maximally 9.5 years, FLAIR intensity further increased. The rate of change of FLAIR intensity within the CST correlated with the rate of decline in executive function and ALS functional rating score. | 1 | 1 | Manual placement of ROIs | T2 hyperintensity CST |
| Feron, 2018 | Extrapyramidal deficits in ALS: a combined biomechanical and neuroimaging study | ALS | Gait “unsteady” ALS patients had reduced caudate and brain stem volumes compared to “steady” ALS patients. | 0 | 0 | FSL VBM | White/gray matter volume |
| Ferraro, 2017 | Multimodal structural MRI in the diagnosis of motor neuron diseases | ALS ("classical" and upper motor neuron disease) | In ALS compared to healthy controls, the combination of cortical thickness and diffusion tensor MRI metrics improved the classification pattern (0.91 accuracy). The combined model distinguished ALS and PUMN patients from mimic syndromes with 0.87 and 0.94 accuracy. | 1 | 0 | Freesurfer | White/gray matter volume |
| Ferraro, 2018 | Perfusion alterations converge with patterns of pathological spread in transactive response DNA-binding protein 43 proteinopathies | ALS, bvALS-TDP | BvFTD patients showed reduced frontotemporal cortical thickness in the orbitofrontal and temporal cortices. ALS patients did not exhibit reduced cortical thickness. | 1 | 0 | AntsCorticalThickness | White/gray matter volume |
| Finegan, 2019 | The clinical and radiological profile of primary lateral sclerosis: a population-based study | ALS, PLS | PLS patients, volume loss in the motor cortex was more medial than in ALS consistent with its lower limb symptom predominance. PLS was associated with considerable cerebellar white and grey matter volume loss as well as marked insular, inferior frontal and left pars opercularis volume loss. In contrast to ALS, PLS spared the postcentral gyrus. The body and splenium of the corpus callosum were mostly affected in PLS, in contrast to the genu involvement observed in ALS. | 1 | 0 | FSL VBM | White/gray matter volume |
| Finegan, 2020 | Widespread subcortical grey matter degeneration in primary lateral sclerosis: a multimodal imaging study with genetic profiling | ALS, PLS | PLS patients exhibited thalamic, caudate, accumbens nuclei and hippocampal volume loss. Hippocampal volume loss in PLS was mainly located in the dentate gyrus, hippocampal tail and CA4 subfield. When comparind ALS and PLS, PLS patients showed bi-thalamic pathology compared to the predominant putaminal degeneration detected in ALS. Another feature more specific for ALS compared to PLS was the preferential volume loss of the amygdala in ALS. | 1 | 0 | FSL VBM, Freesurfer | White/gray matter volume |
| Floeter, 2016 | Longitudinal imaging in C9orf72 mutation carriers: Relationship to phenotype | C9orf72+ (ALS, bvFTD), sporadic ALS | Compared to healthy controls and sporadic ALS patients, symptomatic C9orf72+ carriers exhibited greater ventricular volume loss and thalamic atrophy and patchy cortical thinning. Asymptomatic carriers did not differ from controls. C9orf72+ ALS and ALS-FTD patients had less thinning of the motor cortex than sporadic ALS patients, but more thinning in extramotor regions, particularly in frontal and temporal lobes. C9orf72+ ALS patients differed from sporadic ALS patients in the thickness of the superior frontal gyrus and lateral orbitofrontal cortex. For 6 months follow-up, ventricular volume increased in C9orf72+ patients with FTD and ALS-FTD phenotypes and remained stable in asymptomatic C9orf72+ carriers. | 1 | 1 | SPM12, Freesurfer | White/gray matter volume |
| Frank, 1997 | Relation of neuropsychological and magnetic resonance findings in amyotrophic lateral sclerosis: evidence for subgroups | Sporadic ALS | ALS patients exhibited ventricular enlargement and parenchymal volume loss compared with age-matched controls. | 1 | 0 | Manual planimetric evaluation | White/gray matter volume |
| Geevasinga, 2017 | Brain functional connectome abnormalities in amyotrophic lateral sclerosis are associated with disability and cortical hyperexcitability | ALS | Resting-date connectivity analysis indicated synchronous activity between frontal, parietal, and subcortical regions. These alterations were accompanied by reduction of gray matter volumes in the frontotemporal regions as well as the cerebellar hemispheres and thalamus. | 1 | 0 | VBM8/SPM8 | White/gray matter volume |
| Goodin, 1988 | Magnetic resonance imaging in amyotrophic lateral sclerosis | ALS | 2/5 patients exhibited symmetrical areas of increased signal intensity seen on MRI extending from the cortex, through the corona radiata, posterior limb of the internal capsule, and cerebral peduncles into the pons along the CST. | 0 | 0 | Visual rating | T2 hyperintensity CST |
| Graham, 2004 | Diffusion tensor imaging for the assessment of upper motor neuron integrity in ALS | Upper motor neuron ALS | Motor cortex hypointensity on T2w image contrasts as well as corona radiata hyperintensity on PDw image contrasts distinguished ALS patients with UMN involvement from healthy controls with 100% specificity, but only 20% sensitivity. | 1 | 0 | Visual rating | T1 hypointensity motor cortex, PD hyperintensity CST |
| Grieve, 2015 | Potential structural and functional biomarkers of upper motor neuron dysfunction in ALS | Upper motor neuron ALS | Cortical thinning was evident in the bitemporal regions, while precentral gyrus cortical thinning was evident in about half of ALS patients. | 1 | 0 | Freesurfer | White/gray matter volume |
| Grossman, 2008 | Impaired action knowledge in amyotrophic lateral sclerosis | ALS | Difficulty on measures requiring action knowledge correlated with cortical atrophy in motor cortex. | 0 | 0 | SPM2 | White/gray matter volume |
| Gupta, 2014 | Accuracy of Conventional MRI in ALS | ALS | The overall sensitivity and specificity of conventional MRI for T2w-FLAIR hyperintensities for the diagnosis of ALS were 48% and 76% respectively. Highest specificities for CST hyperintensity were noted for the subcortical white matter (92%), centrum semiovale (88%) and medullary pyramids (92%). The lowest specificities were found for the cerebral peduncle (36%) and internal capsule (32%). | 0 | 0 | Visual rating | T2 hyperintensity CST |
| Hartung, 2014 | Voxel-based MRI intensitometry reveals extent of cerebral white matter pathology in amyotrophic lateral sclerosis | ALS | ALS patients had widespread white matter intensity increases in the CST, corpus callosum, sub-central, frontal and occipital white matter tracts and cerebellum. | 1 | 0 | SPM8/MATLAB, MRIcron | White/gray matter volume |
| Hecht, 2005 | Cortical T2 signal shortening in amyotrophic lateral sclerosis is not due to iron deposits | ALS | Signal shortening in T2w MRI was found in 31/69 patients (45%). In T2*-weighted GRE images, only 3 ALS patients exhibited signal shortening. | 0 | 0 | Visual rating | Motor band sign/signal shortening of the CST |
| Hecht, 2001 | MRI-FLAIR images of the head show corticospinal tract alterations in ALS patients more frequently than T2-, T1- and proton-density-weighted images | ALS | Study assessed signal abnormalities in T2w-FLAIR compared to T2-, T1- and proton-density-weighted images. Hyperintense CST signals were significantly more frequent in T2w-FLAIR compared to other tested sequences. More frequently, but not exclusively in ALS patients, T2w-FLAIR images showed mild hyperintense signals at the subcortical precentral gyrus. In T1w contrasts, the CST at the capsula interna was hypointense in more healthy controls than ALS patients. | 1 | 0 | Visual rating | T2/FLAIR/PD hyperintensity CST (comparing sequences), T1 hypointensity CST |
| Hecht, 2002 | Hyperintense and hypointense MRI signals of the precentral gyrus and corticospinal tract in ALS: a follow-up examination including FLAIR images | ALS | The visual scores of T1w, T2w and PDw hyperintense signals along the CST did not change during the follow-up of around 15 months. However, the quantitative evaluation of T2w-FLAIR revealed an increase of the signal intensity at the subcortical precentral gyrus. | 0 | 1 | Visual rating | T2/FLAIR/PD hyperintensity CST (comparing sequences) |
| Hofmann, 1998 | The corticospinal tract in amyotrophic lateral sclerosis: an MRI study | ALS | The study found no difference in the T2 time in the CST between ALS patients and healthy controls. images the patients' ratings did not differ from that of controls. But ALS patients had higher PDw signal intensity along the CST compared to healthy controls | 1 | 0 | Visual rating, manual T2 measurement | T2 hyperintensity CST |
| Ignjatovic, 2013 | Brain iron MRI: a biomarker for amyotrophic lateral sclerosis | ALS | T2*w contrasts were superior to any other conventional MRI sequence in detecting hypointensities (i.e., iron deposits) in the brain of ALS patients. Iron deposits were found only in the precentral gyruses gray matter and were detected in 42/47 patients (89%). These hypointensities further increased during a 6 month follow-up period. | 1 | 1 | Visual rating | Motor band sign |
| Irwin, 2013 | Cognitive decline and reduced survival in C9orf72 expansion frontotemporal degeneration and amyotrophic lateral sclerosis | ALS and FTLD (both C9orf72+ vs -) | C9orf72+ carriers showed greater atrophy in the right frontoinsular, thalamus, cerebellum and bilateral parietal regions compared to C9orf72- subjects. | 0 | 1 | Pipedream, ANTS | White/gray matter volume |
| Ishikawa, 1993 | Signal loss in the motor cortex on magnetic resonance images in amyotrophic lateral sclerosis | ALS | 3/7 ALS patients (43%) showed bilateral signal loss confined to the motor cortex on T2-weighted magnetic resonance images. None of the control subjects had this findings. | 1 | 0 | Visual rating | T2 hypointensity motor cortex |
| Iwasaki, 1989 | Central nervous system magnetic resonance imaging findings in amyotrophic lateral sclerosis | ALS | 2/5 ALS patients (40%) had asymmetrical areas of increased signal intensity in the white matter on T2w image contrast. | 0 | 0 | Visual rating | T2 hyperintensity CST |
| Iwasaki, 1991 | MRI in patients with amyotrophic lateral sclerosis: correlation with clinical features | ALS | 4/10 ALS patients (40%) had asymmetrical areas of increased signal intensity in the white matter on T2w contrasts. | 0 | 0 | Visual rating | T2 hyperintensity CST |
| Jin, 2019 | Dominant Heterogeneity of Upper and Lower Motor Neuron Degeneration to Motor Manifestation of Involved Region in Amyotrophic Lateral Sclerosis | ALS | ALS patients had thinner cortices compared to healthy controls on bilateral head-face and upper-limb areas. In head-face area, the cortical thickness of bulbar-onset group was lower compared to that of the control groups. In upper-limb areas, the cortical thickness of cervical-onset group was thinner compared to that of the control group. | 1 | 0 | Freesurfer | White/gray matter volume |
| Jin, 2016 | Hyperintensity of the corticospinal tract on FLAIR: A simple and sensitive objective upper motor neuron degeneration marker in clinically verified amyotrophic lateral sclerosis | ALS | CST hyperintensities were more common in ALS compared to healthy controls in subcortical precentral gyrus, centrum semiovale, posterior limbs of internal capsule and cerebral peduncles levels. The three observers' rataings were identical regarding CST hyperintensity on T2w-FLAIR images in subcortical precentral gyrus, centrum semiovale, internal capsule, and cerebral peduncles levels. | 1 | 0 | Visual rating | T2 hyperintensity CST |
| Josephs, 2013 | Corticospinal tract degeneration associated with TDP-43 type C pathology and semantic dementia | ALS | Of the 12 cases with volumetric MRI, 5 were classified as CSTD(−), i.e., without CST degeneration, five were classified as CSTD(±) and two were classified as CSTD(+). Grey matter loss in all three groups was mostly restricted to the temporal lobes. Both the CSTD(−) and CSTD(±) groups had greater involvement of the left hemisphere, with loss observed in anterior regions of the left temporal lobe, involving temporal pole, fusiform gyrus, inferior and middle temporal gyrus, amygdala and hippocampus. In contrast, the CSTD(+) patients had more bilateral patterns of temporal lobe loss, with greater involvement of the right hemisphere. The temporal pole, fusiform gyrus, inferior and middle temporal gyrus, amygdala, and hippocampus were involved in both hemispheres, although to a greater degree on the right. The CSTD(+) cases showed greater volume loss in the right amygdala, fusiform gyrus, parahippocampal gyrus, superior temporal gyrus and bilateral superior motor cortices, than the group of subjects that consisted of both the CSTD(−) and CSTD(±) cases. | 0 | 0 | SPM5, FSL VBM | White/gray matter volume |
| Kamminga, 2016 | Syntactic comprehension deficits across the FTD-ALS continuum | ALS, FTD-ALS | the FTD-ALS demonstrated widespread volume loss of the frontal and temporal regions compared to the healthy controls. No significant volume loss was detected in the ALS group compared to healthy controls. | 1 | 0 | FSL VBM | White/gray matter volume |
| Kassubek, 2005 | Global brain atrophy and corticospinal tract alterations in ALS, as investigated by voxel-based morphometry of 3-D MRI | ALS | In ALS patients, brain parenchymal fraction was reduced compared to healthy controls. ALS patients showed regional volume loss in the right‐hemispheric primary motor cortex and in the left medial frontal gyrus. Also, ALS patients had regional white matter alterations along the CST bilaterally and in multiple smaller areas including corpus callosum, cerebellum, frontal and occipital subcortical regions. | 1 | 0 | SPM99 | White/gray matter volume and T2 hyperintensity CST |
| Kato, 1993 | Involvement of the frontotemporal lobe and limbic system in amyotrophic lateral sclerosis: as assessed by serial computed tomography and magnetic resonance imaging | Sporadic ALS | ALS patients showed progressive atrophy during follow-up (unclear follow-up time), first in the frontal and anterior temporal lobes then in the precentral gyri, and later in the postcentral gyrus, anterior part of the cingulate gyms, corpus callosum and brain stem tegmentum. Additionally, T2w contrasts showed high intensity signals on T2w images in the precentral and adjacent gyri, frontotemporal white matter and pyramidal tract as well as rarely in the globus pallidus and thalamus. | 0 | 1 | Visual rating | White/gray matter volume and T2 hyperintensity CST |
| Keller, 2011 | Quantitative brain MR imaging in amyotrophic lateral sclerosis | ALS | ALS patients showed correlation between decreased white matter intensity in the corona radiata and the limb component of ALS-FRS. | 1 | 0 | Visual rating | White/gray matter volume |
| Kiernan, 1994 | Frontal lobe atrophy in motor neuron diseases | Sporadic ALS, PLS | In PLS, the surface area of the precentral gyri and underlying white matter were around 75% of healthy controls. Also, anterior to the precentral sulci, the cortical surface area in PLS was around 85% of that of the controls. By contrast, in ALS patients, no difference compared to controls. Also, the cortical surface areas of the anterior frontal lobes were not different compared to controls. | 1 | 0 | Manual tracing | White/gray matter volume |
| Kim, 2017 | Relationship between Clinical Parameters and Brain Structure in Sporadic Amyotrophic Lateral Sclerosis Patients According to Onset Type: A Voxel-Based Morphometric Study | Sporadic ALS (limb onset and bulbar onset) | In limb-onset ALS patients, volume loss was largely confined to the motor cortex and adjacent pre- and postcentral regions. In contrast, in the bulbar-onset group, affected brain regions were more widespread and extended to the bilateral fronto-temporal and left superior temporal and supramarginal gyri. | 1 | 0 | SPM12/VBM8 | White/gray matter volume |
| Kim, 2017 | Structural explanation of poor prognosis of amyotrophic lateral sclerosis in the non-demented state | Sporadic ALS (pure, cognitive impairment and behavioral impairment) | The ALS cognitively impaired group exhibited decreased volume in the left cerebellum, fusiform gyrus, optic radiations, and CST compared to healthy controls. These ALS patients also showed decreased brain volume in the bilateral cerebellum, right putamen gray matter and bilateral superior longitudinal fasciculi white matter compared to pure ALS patients. Compared to healthy controls, pure ALS and behavioraly impaired ALS patients did not show any volume changes in gray and white matter. | 1 | 0 | SPM12 | White/gray matter volume |
| Koike, 2015 | Apparent diffusion coefficients distinguish amyotrophic lateral sclerosis from cervical spondylotic myelopathy | Sporadic ALS | ALS patients exhibited higher ADCs in the precentral gyrus, the posterior limb of the internal capsule, and the cerebral peduncle compared to healthy controls, yet this difference was not confirmed visually on the ADC maps. | 1 | 0 | Manual placement of ROIs | ADC |
| Konno, 2013 | Japanese amyotrophic lateral sclerosis patients with GGGGCC hexanucleotide repeat expansion in C9ORF72 | Familial ALS (c9FTD), sporadic ALS | MRI showed mild diffuse brain atrophy in all ALS patients. | 1 | 0 | Visual rating | White/gray matter volume |
| Kono, 2014 | Clinical characteristics associated with corticospinal tract hyperintensity on magnetic resonance imaging in patients with amyotrophic lateral sclerosis | ALS (definite and indefinite phase) | ALS patients were divided into two groups: a positive CST group showing CST hyperintensity; and a negative CST group. 8/17 patients (47%) showed CST positivity. The rate of CST positivity was higher in patients with definite phase ALS (75%) than in patients with indefinite phase ALS (22%). | 0 | 0 | Visual rating | T2 hyperintensity CST |
| Kuipers-Upmeijer, 2001 | Primary lateral sclerosis: clinical, neurophysiological, and magnetic resonance findings | PLS | PLS patients had cortical atrophy, most pronounced in the precentral gyrus and expanding into the parietal-occipital region. Cervical MRI or myelography was normal for all PLS patients. | 0 | 0 | Visual rating | White/gray matter volume |
| Kwan, 2012 | Iron accumulation in deep cortical layers accounts for MRI signal abnormalities in ALS: correlating 7 tesla MRI and pathology | ALS | The motor cortex hypointensity on 3T T2w-FLAIR images were more common in ALS patients. On 7T T2*w GRE, the signal alterations were localized to the deeper layers of the motor cortex in both ALS patients. Histopathology showed increased iron accumulation in microglial cells in areas corresponding to the location of the signal changes on the MRI in the motor cortex. | 1 | 0 | Visual rating | T2/FLAIR hypointensity motor cortex |
| Kwan, 2012 | Structural imaging differences and longitudinal changes in primary lateral sclerosis and amyotrophic lateral sclerosis | ALS, PLS | In the cross-sectional analysis, both ALS and PLS patients had areas of cortical thinning, which was more extensive in motor regions in PLS patients. At follow-up after a mean time of 1.25 years, cortical thinning and grey matter volume loss of the precentral gyri progressed. The rate of cortical thinning was greater in ALS patients with shorter disease durations, suggesting that thickness decreases in a non-linear fashion. | 1 | 1 | Freesurfer | White/gray matter volume |
| Le Ber, 2008 | Phenotype variability in progranulin mutation carriers: a clinical, neuropsychological, imaging and genetic study | frontal variant FTD (fvFTD), FTD with motoneuron disease (FTD-MND) and primary progressive aphasia (PPA) (with GRN mutations) | MRI, acquired after a mean disease duration of 2.8 years showed frontal and/or temporal atrophy in almost all patients. Cortical atrophy was asymmetric in 16/21 mutation carriers (76%), with right predominance in 10/21 (48%) and left predominance in 6/21 (29%). Parietal/occipital atrophy was present in 10 patients. Small patchy or more extensive white matter lesions were present on T2w image contrasts in 4/21 patients (19%). | 0 | 1 | SPM2 | White/gray matter volume and T2 hyperintensity (CST?) |
| Lee, 2017 | Quantitative susceptibility mapping of the motor cortex: a comparison of susceptibility among patients with amyotrophic lateral sclerosis, cerebrovascular disease, and healthy controls | ALS | There were significant differences in the mean cortex, max cortex, mean relative susceptibility (from QSM), and max relative susceptibility among ALS and healthy controls, with higher values in patients with ALS. Subcortical white matter mean was lower in patients with ALS compared with healthy controls. | 1 | 0 | Manual tracing | QSM |
| Lee, 2014 | Altered network connectivity in frontotemporal dementia with C9orf72 hexanucleotide repeat expansion | behavioural variant frontotemporal dementia (C9orf72 positive or negative carriers) with comorbid motor neuron disease | C9orf72+ bvFTD showed volume loss in bilateral anterior cingulate, dorsolateral prefrontal, orbitofrontal, anterior, and posterior insular, and lateral parietal cortices, and precuneus, striatum, and bilateral thalamus compared to healthy controls. C9orf72- bvFTD patients showed similar but more extensive bilateral atrophy in frontotemporal, insular, cingulate, and striatal regions, with less extensive thalamic volume loss compared with healthy controls. C9orf72+ bvFTD exhibited greater volume loss in bilateral medial pulvinar thalamic nuclei, postcentral gyrus, precuneus, and lateral parietal cortex, whereas C9orf72- bvFTD patients showed greater volume loss in bilateral anterior cingulate cortex, medial superior frontal gyri, and anterior insulae and left striatum | 1 | 0 | SPM8/VBM8 | White/gray matter volume |
| Lee, 2017 | Network degeneration and dysfunction in presymptomatic C9ORF72 expansion carriers | C9orf72 presymptomatic carriers | Gray matter volume loss was topographically similar though less severe than those in patients with behavioral variant frontotemporaldementiadue to C9ORF72, with foci in cingulate, insula, thalamus, and striatum. Reduced white matter integrity was found in the corpus callosum, cingulum bundles, CST, uncinate fasciculi and inferior longitudinal fasciculi. | 1 | 0 | SPM12 | White/gray matter volume |
| Leslie, 2015 | Semantic deficits in amyotrophic lateral sclerosis | ALS, ALS-FTD | The ALS-FTD groups had more brain volume loss compared to healthy controls, but the ALS group did not differ from controls. ALS-FTD patients demonstrated volume loss of the anterior temporal lobes bilaterally. | 1 | 0 | Not reported | White/gray matter volume |
| Libon, 2012 | Deficits in concept formation in amyotrophic lateral sclerosis | ALS | ALS patients exhibited widespread cortical thinning involving bilateral frontal, temporal and parietal regions. | 0 | 0 | Not reported | White/gray matter volume |
| Lillo, 2012 | Grey and white matter changes across the amyotrophic lateral sclerosis-frontotemporal dementia continuum | ALS, ALS-FTD, bvFTD | ALS patients mainly showed volume changes in the motor cortex and anterior cingulate as well as their underlying white matter tracts. ALS-FTD and bvFTD showed widespread grey and white matter changes involving frontal and temporal lobes. Extensive prefrontal cortex changes were observed in bvFTD compared to other subtypes, while ALS-FTD could be discriminated from ALS by additional temporal lobe grey and white matter alterations. Finally, ALS could be mainly distinguished from the other two groups by CST degeneration. | 1 | 0 | FSL VBM | White/gray matter volume |
| Luis, 1990 | Magnetic resonance imaging in motor neuron disease | ALS | 8/20 (40%) of ALS patient showed T2w hyperintensities in the centrum semiovale, corona radiata, internal capsule, pedunculi of midbrain, pons, medulla and even in the frontal lobe, topographically related with the CST. | 1 | 0 | Visual rating | T2 hyperintensity CST |
| Machts, 2018 | Prefrontal cortical thickness in motor neuron disease | classical ALS and bulbar phenotype, PLS, upper motor neuron dominant ALS, flail limb, progressive muscular atrophy, ALS-FTD | MND patients showed cortical thinning in the right motor cortex: 29.1% in the right motor cortex. Left prefrontal cortical thickness was reduced in patients with additional cognitive and/or behavioural deficits compared to MND patients without cognitive decline. | 1 | 0 | Freesurfer | White/gray matter volume |
| Machts, 2015 | Basal ganglia pathology in ALS is associated with neuropsychological deficits | ALS-FTD, ALS plus, ALS without cognitive impairment (all C9orf72-) | Significant basal ganglia volume differences were identified between all study groups. Non-cognitively impaired ALS patiens exhibited distinct atrophy patterns in the amygdala, ALS plus patients in the hippocampus in patients with ALS-Plus in comparison with healthy controls. Patients with ALS-FTD exhibited pathologic alterations in the bilateral thalami, putamina, pallida, hippocampi, caudate and accumbens nuclei in comparison with all other study groups. | 1 | 0 | FSL VBM | White/gray matter volume |
| Machts, 2018 | Global Hippocampal Volume Reductions and Local CA1 Shape Deformations in Amyotrophic Lateral Sclerosis | ALS | Left and right hippocampal volumes were reduced in ALS patients compared to healthy controls. Local shape alterations were identified in the hippocampal head region of patients with ALS corresponding to the cornu ammonis field 1 (CA1). | 1 | 0 | FSL VBM, Freesurfer | Hippocampus |
| Mahoney, 2012 | Frontotemporal dementia with the C9ORF72 hexanucleotide repeat expansion: clinical, neuroanatomical and neuropathological features | ALS (C9orf72+) | Compared to healthy controls, the ALS group showed extensive thinning of frontal, temporal and parietal cortices, subcortical grey matter atrophy including thalamus and cerebellum and involvement of long intrahemispheric, commissural and CST. | 0 | 0 | MIDAS | White/gray matter volume |
| Mahoney, 2012 | Longitudinal neuroimaging and neuropsychological profiles of frontotemporal dementia with C9ORF72 expansions | ALS-FTLD (C9orf72+ or -) | Mean rates of whole brain atrophy (1.4%/year) and ventricular expansion (3.2 ml/year) were greater in patients with the C9orf72 mutation than in healthy controls. Brain atrophy was symmetrical in the cerebral hemispheres within the C9ORF72 mutation group. The thalamus and cerebellum showed atrophy whereas no cortical areas were preferentially affected. Longitudinal fluid imaging in individual patients demonstrated heterogeneous patterns of progressive atrophy. | 1 | 1 | MIDAS | White/gray matter volume |
| McCluskey, 2014 | ALS-Plus syndrome: non-pyramidal features in a large ALS cohort | ALS plus | MRI analysis showed greater cerebellar and cerebral volume loss in ALS plus compared to those without ALS plus. | 0 | 0 | SPM8 | White/gray matter volume |
| McMillan, 2015 | C9orf72 promoter hypermethylation is neuroprotective: Neuroimaging and neuropathologic evidence | ALS (C9orf72+) | In c9orf72+ ALS patients, longitudinal MRI (mean follow-up 13 months) showed that hypermethylation of c9orf72 is associated with reduced longitudinal decline in gray matter regions. | 1 | 1 | FSL VBM | White/gray matter volume |
| Menke, 2014 | Widespread grey matter pathology dominates the longitudinal cerebral MRI and clinical landscape of amyotrophic lateral sclerosis | Sporadic ALS | In the cross-sectional study, the core signature of white matter pathology was confirmed within the CST and corpus callosum. Localized gray matter abnormalities were detected in the left motor cortex and in Broca's area. Longitudinal analysis (max follow-up 30 months) showed progressive and widespread changes in the grey matter, including the basal ganglia. In contrast, there was only limited white matter pathology progression. Although a consistent core white matter pathology was found cross-sectionally, grey matter pathology was dominant longitudinally and included progression in regions such as the basal ganglia. | 1 | 1 | FSL VBM | White/gray matter volume |
| Menke, 2018 | The two-year progression of structural and functional cerebral MRI in amyotrophic lateral sclerosis | ALS, PLS | Widespread and progressive reductions in grey matter were observed in the precentral gyri and posterior cingulate cortex during the 2 year follow-up period, as well as progressive local atrophy of the thalamus, caudate as well as pallidum bilaterally, and right putamen, hippocampus and amygdala. | 1 | 1 | FSL VBM | White/gray matter volume |
| Meoded, 2013 | Imaging findings associated with cognitive performance in primary lateral sclerosis and amyotrophic lateral sclerosis | ALS, PLS | PLS patients showed reduced gray matter volumes in motor and perirolandic areas. | 1 | 0 | SPM8 | White/gray matter volume |
| Mezzapesa, 2007 | Whole-brain and regional brain atrophy in amyotrophic lateral sclerosis | ALS | In ALS patients, brain parenchymal fraction was lower compared with healthy controls. Also, ALS patients showed a gray matter volume loss in several frontal and temporal areas bilaterally compared with healthy subjects, with a prevalence in the right hemisphere. No volume reduction in primary motor cortices of patients was observed. | 1 | 0 | FSL VBM | White/gray matter volume |
| Mezzapesa, 2013 | Cortical thinning and clinical heterogeneity in amyotrophic lateral sclerosis | ALS | ALS patients showed cortical thinning in bilateral precentral gyrus, bilateral middle frontal gyrus and right occipital cortex. Compared to healthy controls, ALS patients with increased upper motor neuron burden showed a cortical thinning in the right precentral gyrus and in other frontal extra-motor areas. Compared to healthy controls, ALS patients with spinal onset showed a significant cortical thinning in the right precentral gyrus and paracentral lobule. the ROI analysis demonstrated that the mean cortical thickness values were significantly reduced in ALS patients with higher upper motor neuron burden, spinal onset and faster disease progression related to healthy controls | 1 | 0 | Freesurfer | White/gray matter volume |
| Minnerop, 2009 | In vivo voxel-based relaxometry in amyotrophic lateral sclerosis | ALS | ALS patients exhibited a reduction of white matter in the paracentral lobules and in the right middle cerebellar peduncle compared to healthy controls. | 1 | 0 | SPM2 | White/gray matter volume |
| Mioshi, 2013 | Cortical atrophy in ALS is critically associated with neuropsychiatric and cognitive changes | ALS, ALS-FTD, ALS plus | ALS plus patients showed volume loss across motor and somatosensory as well as adjacent frontal and parietal areas. In contrast, patients with ALS had no cortical volume loss but only brainstem atrophy. Volume loss in ALS plus was not as widespread as in ALS-FTD, with ALS plus volume loss mostly confined to motor and somatosensory areas, while volume loss in ALS-FTD also included substantial frontal and temporal volume loss. | 1 | 0 | FSL VBM | White/gray matter volume |
| Miwa, 2003 | T2-low signal intensity in the cortex in multiple system atrophy | ALS | ALS patient showed increase in the frequency of T2-low signal intensity in the cortex. | 1 | 0 | Visual rating | T2 hypointensity motor cortex |
| Mori, 2007 | Symmetric temporal abnormalities on MR imaging in amyotrophic lateral sclerosis with dementia | ALS with or without dementia | ALS patients with dementia showed bilateral frontotemporal volume loss mostly with temporal lobe dominance. In this group, T2w contrasts also demonstrated hyperintensity in the subcortical white matter on the medial side of the anterior temporal lobes. This abnormality was not seen in ALS patients without dementia. MRI demonstrated no abnormal signal-intensity in CST in the internal capsule or the brain stem in the ALS with dementia group. In the group without dementia, 6 patients (29%) showed CST hyperintensities. | 1 | 0 | Visual rating | White/gray matter volume and T2 hyperintensity CST |
| Muller, 2020 | Focal alterations of the callosal area III in primary lateral sclerosis: An MRI planimetry and texture analysis | ALS, PLS | PLS patients showed volume loss in the area III of the CC (according to Hofer and Frahm), while the alterations in the ALS patients were more variable and were not significant at the group level. | 1 | 0 | Automated segmentation of corpus callosum | Corpus callosum volume |
| Muller, 2011 | Complementary image analysis of diffusion tensor imaging and 3-dimensional t1-weighted imaging: white matter analysis in amyotrophic lateral sclerosis | ALS | ALS patients showed T1w signal intensity changes bilaterally in the CST in the projection to the posterior limb of the internal capsule. | 1 | 0 | Visual rating | Altered T1 signal intensity |
| Murphy, 2007 | Continuum of frontal lobe impairment in amyotrophic lateral sclerosis | ALS (familial and sporadic) | ALS patients with cognitive and behavioral deficits had reduced volumes as compared with the normal controls in all regions of interest. The cognitively and behaviorally intact ALS patients had larger right frontal, right parietal, and right limbic volumes as compared with the patients with ALS with cognitive and behavioral abnormalities. Cognitively and behaviorally intact ALS patients had decreased right temporal volumes as compared with normal controls. | 1 | 0 | FSL VBM | White/gray matter volume |
| Nasseroleslami, 2019 | Characteristic Increases in EEG Connectivity Correlate With Changes of Structural MRI in Amyotrophic Lateral Sclerosis | ALS, ALS-FTD | in all ALS groups, disease-specific structural degeneration in motor areas and CSt was associated with a decrease in neural activity over scalpmotor areas. | 1 | 0 | Not reported | White/gray matter volume |
| Ngai, 2007 | Hyperintensity of the precentral gyral subcortical white matter and hypointensity of the precentral gyrus on fluid-attenuated inversion recovery: variation with age and implications for the diagnosis of amyotrophic lateral sclerosis | Non-ALS patients | Study shows a relationship between increasing age and the frequency of precentral gyrus subcortical white matter hyperintensity and precentral gyrus gray matter hypointensity on T2w-FLAIR in non-ALS patients. | 1 | 0 | Visual rating | T2 hyperintensity CST, hypointensity motor cortex |
| Oba, 1993 | Amyotrophic lateral sclerosis: T2 shortening in motor cortex at MR imaging | ALS | 14/15 ALS patients (93%) showed T2 shortening in precentral cortices, while the images of all but one of the control patients showed no such finding. In 3/8 brains at autopsy, sections from the precentral cortex showed sparsely distributed, intensely stained astrocytes and macrophages. | 1 | 0 | Visual rating | T2 hypointensity motor cortex |
| Obusez, 2018 | 7T MR of intracranial pathology: Preliminary observations and comparisons to 3T and 1.5T | ALS | All 9 ALS patients demonstrated decreased signal intensity of the primary motor cortex on the posterior bank of the precentral gyrus on both 7T SWI and T2 FLAIR, and decreased signal on corresponding T2*w images. On retrospective review of 3T SWI sequences, the decreased signal intensity along the primary motor cortex was observed in only 6 patients and was seen poorly in 2 patients. One patient, imaged on a 1.5T scanner, did not have observable signal abnormalities. | 0 | 0 | Visual rating | Motor band sign |
| Omer, 2017 | Neuroimaging patterns along the ALS-FTD spectrum: a multiparametric imaging study | behavioural variant FTD (bvFTD), non-fluent-variant primary progressive aphasia (nfvPPA), sematic-variant primary progressive aphasia (svPPA), ALS-FTD (C9orf72+ or -), ALS without behavioural or cognitive deficits (ALSnci) | Phenotype-specific spatial patterns of brain pathology were identified along the ALS-FTD spectrum, emphasizing an apparently focal distribution of disease burden as opposed to global atrophy. Significant motor cortex and CST degeneration was identified in both bvFTD and nfvPPA patients. C9+ ALS-FTD patients showed widespread extramotor pathology and significant precentral gyrus atrophy compared to ALSnci patients. ROI analyses showed focal grey matter alterations in Brocas and Wernickes area in language variant FTD cohorts. | 1 | 0 | FSL VBM | White/gray matter volume |
| Papma, 2017 | Cognition and gray and white matter characteristics of presymptomatic C9orf72 repeat expansion | Presymptomatic C9orf72+ carriers | In a subgroup of presymptomatic C9orf72+ carriers above a certain age (>40 years), study found gray matter volume loss in the thalamus, cerebellum, and parietal and temporal cortex. | 1 | 0 | FSL VBM | White/gray matter volume |
| Paquin, 2018 | Spinal Cord Gray Matter Atrophy in Amyotrophic Lateral Sclerosis | ALS | Gray matter atrophy was more sensitive to discriminate patients with amyotrophic lateral sclerosis from healthy controls compared with spinal cord atrophy. Prediction at 1 year with clinical scores was enhanced when including a combination of gray and white matter cross-sectional areas. | 1 | 1 | PropSeg | White/gray matter volume |
| Peretti-Viton, 1999 | MRI of the intracranial corticospinal tracts in amyotrophic and primary lateral sclerosis | ALS, PLS | No abnormalities were observed in the CST on T1w images and were only rarely seen on PDw images. Variable high signal in the CST was found on T2w images in 35/39 patients (90%), and in 29/37 control subjects (78%). | 1 | 0 | Visual rating | T2 hyperintensity CST |
| Piaggio, 2018 | Cord cross-sectional area at foramen magnum as a correlate of disability in amyotrophic lateral sclerosis | ALS | Spinal cord area at the foramen magnum was lower in ALS patients compared to control subjects. | 1 | 0 | JIM | Spinal cord atrophy |
| Pinkhardt, 2006 | Amygdala size in amyotrophic lateral sclerosis without dementia: an in vivo study using MRI volumetry | ALS without cognitive or behavioural deficits | A trend for reduced amygdala size was seen in the ALS group. | 1 | 0 | Semiautomated thresholding | Amygdala size |
| Pinto, 2019 | O'Sullivan-McLeod syndrome: Unmasking a rare atypical motor neuron disease | O’Sullivan–McLeod syndrome | Neuroimaging studies were unremarkable. | 0 | 0 | Visual rating | N/A |
| Placek, 2019 | UNC13A polymorphism contributes to frontotemporal disease in sporadic amyotrophic lateral sclerosis | Sporadic ALS | Study identified reduced cortical thickness in sporadic ALS with T1w imaging compared to healthy controls. Minor allele carriers showed greater reduction of cortical thickness in dorsal prefrontal, ventromedial prefrontal, anterior temporal, and middle temporal cortices. | 1 | 0 | ANT, FSL-VBM | White/gray matter volume |
| Prell, 2015 | Susceptibility-weighted imaging provides insight into white matter damage in amyotrophic lateral sclerosis | ALS | In ALS patients, signal alterations were seen on SWI in the corpus callosum; along the CST (subcortical motor cortex, posterior limb of the internal capsule and brainstem levels) and in the subgyral regions of frontal, parietal, temporal, occipital and limbic lobes. | 1 | 0 | Visual rating | SWI signal alterations (CC, CST, cortex) |
| Prell, 2014 | Transcranial brainstem sonography as a diagnostic tool for amyotrophic lateral sclerosis | Sporadic ALS | 11 of 67 patients with ALS exhibited hyperintensities along the CST (16%) | 0 | 0 | Visual rating | T2 hyperintensity CST |
| Protogerou, 2011 | T2 FLAIR Increased Signal Intensity at the Posterior Limb of the Internal Capsule: Clinical Significance in ALS Patients | ALS | On T2w-FLAIR, signal changes were observed in the posterior limb of the internal capsule in 7/24 ALS patients (29%) and in 10/51 healthy subjects (20%). Distinct T2w-FLAIR signal changes were seen in the posterior limb of the internal capsule in seven ALS patients. No distinct signal change was visualized in any of the healthy controls. These signal changes became more accentuated with disease progress. | 1 | 1 | Visual rating | T2 hyperintensity CST |
| Qin, 2018 | Region-specific atrophy of precentral gyrus in patients with amyotrophic lateral sclerosis | ALS (early and late stage) | Both early and leat stage ALS showed volume loss of the dorsal-lateral part of precentral gyrus. Volume loss was more widespread in late compared to early stage ALS: while volume loss in early stage ALS was mostly confined to the dorsal-lateral region, volume loss in late stage ALS occurred at the dorsal-medial and ventral region as well. | 1 | 0 | diffeomorphic multi-atlas likelihood-fusion (MALF), BrainWorks | White/gray matter volume |
| Qiu, 2019 | Precentral degeneration and cerebellar compensation in amyotrophic lateral sclerosis: A multimodal MRI analysis | ALS | Compared with healthy controls, ALS patients had decreased gray matter volume in the left precentral gyrus and increased gray matter volume bilaterally in the cerebellum. | 1 | 0 | SPM8 | White/gray matter volume |
| Querin, 2019 | Presymptomatic spinal cord pathology in c9orf72 mutation carriers: A longitudinal neuroimaging study | ALS, FTD (C9orf72+ or -) | At baseline, C9orf72+ carriers over 40 years had higher volume loss at the cervical vertebral levels without associated changes in gray matter or total cross-sectional spinal cord area. | 0 | 1 | Spinal cord toolbox | Spinal cord atrophy |
| Querin, 2017 | Spinal cord multi-parametric magnetic resonance imaging for survival prediction in amyotrophic lateral sclerosis | ALS | Study aimed to determine the predictive added value of multimodal spinal cord MRI on ALS survival. In a multivariate Cox regression model with clinical and MRI parameters, fractional anisotropy, magnetization transfer ratio and spinal cord cross-sectional area at C2–C3, C4–C5, C5–C6 and C6–C7 vertebral levels were significant. Moreover, the hazard ratio calculated for cross-sectional area at the C3–C4 and C5–C6 levels suggested an increased risk for patients with spinal cord atrophy. | 0 | 1 | Manual segmentation | Spinal cord atrophy |
| Raaphorst, 2015 | Prose memory impairment in amyotrophic lateral sclerosis patients is related to hippocampus volume | ALS (without dementia) | in ALS, story recall scores correlated to bilateral hippocampus grey matter volume. | 1 | 0 | Diffeomorphic Anatomical Registration Through Exponentiated Lie (DARTEL)/SPM5 | Hippocampus volume |
| Radakovic, 2018 | Frontostriatal grey matter atrophy in amyotrophic lateral sclerosis A visual rating study | ALS, ALS-FTD | ALS patients displayed higher atrophy ratings in the bilateral medial orbitofrontal cortex compared to healthy controls. Patients with greater medial orbitofrontal cortex atrophy had higher atrophy of the caudate nucleus and lateral orbitofrontal cortex. | 1 | 0 | Visual rating | Spinal cord atrophy |
| Rajagopalan, 2014 | Distinct patterns of cortical atrophy in ALS patients with or without dementia: an MRI VBM study | predominant upper motor neuron dysfunction with or without CST hyperintensity (ALS-CST+/–), combined UMN and prominent lower motor neuron dysfunction (classic ALS), frontotemporal dementia (ALS-FTD) | ALS-FTD patients had reduced gray matter volume, but not predominant UMN dysfunction or classic ALS, compared to neurologic disease controls. | 1 | 0 | FSL VBM | T2 hyperintensity CST and white/gray matter volume |
| Rajagopalan, 2015 | Brain Parenchymal Fraction: A Relatively Simple MRI Measure to Clinically Distinguish ALS Phenotypes | ALS with or without CST hyperintensities on T2w-FLAIR, ALS with cognitive impairment, ALS-FTDT− ALS-Cl ALS-FTD | Only ALS-FTD patients had significant reduction in brain parenchymal fraction in comparison with controls and nondemented ALS patients. | 1 | 0 | SPM8 | T2/PD hyperintensity CST and white/gray matter volume |
| Rajagopalan, 2015 | Comparing brain structural MRI and metabolic FDG-PET changes in patients with ALS-FTD: 'the chicken or the egg?' question | ALS-FTD | In ALS-FTD, significant reductions in gray matter volume and cortical thickness were observed in motor and extramotor regions compared to healthy controls. No difference in cortical surface area was observed in any of the assessed brain regions | 1 | 0 | FSL VBM | White/gray matter volume |
| Rajagopalan, 2015 | Disparate voxel based morphometry (VBM) results between SPM and FSL softwares in ALS patients with frontotemporal dementia: which VBM results to consider? | ALS-FTD | Gray matter volume was reduced in both motor and extra motor regions in ALS- FTD when compared to healthy controls. Gray matter volume changes using FSL showed a similar pattern like Freesurfer cortical volume and thickness changes in contrast to SPM results. | 1 | 0 | SPM8, Freesurfer, FSL VBM | White/gray matter volume |
| Rajagopalan, 2014 | Do preprocessing algorithms and statistical models influence voxel-based morphometry (VBM) results in amyotrophic lateral sclerosis patients? A systematic comparison of popular VBM analytical methods | ALS-FTD | Percentage of atrophied gray matter voxels in the entire brain that reached statistical significance using FSL was 22.52% compared to 0.81% in SPM. Similarly, 0.81% reached statistical significance using nonparametric statistics when compared to parametric statistics. | 1 | 0 | SPM8, FSL VBM | White/gray matter volume |
| Ramanathan, 2018 | Demographics and clinical characteristics of primary lateral sclerosis: case series and a review of literature | PLS | None of the PLS patients showed abnormal neuroimaging findings. | 0 | 0 | Visual rating | N/A |
| Roeben, 2019 | The motor band sign in ALS: presentations and frequencies in a consecutive series of ALS patients | ALS | in ALS patients, motor band sign was present in 8/157 ALS patients (5%) on clinical routine MRI (T2w, T2*w, T2w-FLAIR, DWI), but in 78% of patients where susceptibility-weighted imaging was available. | 0 | 0 | Visual rating | Motor band sign |
| Sarchielli, 2001 | Magnetic resonance imaging and 1H-magnetic resonance spectroscopy in amyotrophic lateral sclerosis | ALS | On T2w contrast, study found high signal in the CST in 6/12 (50%) and low signal in the primary motor cortex in 7/12 patients (58%). Volume loss of the precentral gyrus was apparent in all the patients apart from one with probable ALS. | 0 | 0 | Visual rating | T2 hyperintensity CST and hypointensity motor cortex |
| Sasaki, 1999 | Atypical form of amyotrophic lateral sclerosis | Upper limb ALS | Cervical MRI without pathological findings. | 0 | 0 | Visual rating | N/A |
| Schonecker, 2018 | Atrophy in the Thalamus But Not Cerebellum Is Specific for C9orf72 FTD and ALS Patients - An Atlas-Based Volumetric MRI Study | ALS, FTD/ALS (C9orf72+ or -) | Compared to sporadic patients, C9orf72+ carriers exhibited a volume reduction of the thalamus, most strikingly in the occipital, temporal and prefrontal subregion of the thalamus. No significant atrophy of cerebellar regions was observed. | 1 | 0 | Diffeomorphic anatomical registration through exponentiated Lie algebra (DARTEL), SPM | White/gray matter volume |
| Schuster, 2014 | Cortical thinning and its relation to cognition in amyotrophic lateral sclerosis | ALS, ALS-FTD, ALS with or without cognitive impairment | Compared with healthy controls, the ALS patients exhibited cortical thinning in the bilateral precentral gyrus, right precuneus, and right frontal and temporal lobes. ALS-FTD patients showed cortical thinning in regions including the frontal and temporal gyri and the posterior cingulate cortex. Cognitively impaired ALS patients showed cortical thinning in regions largely overlapping with those found in ALS-FTD, but changes were less widespread. In conclusion, the cognitive status of ALS subjects is associated with different patterns of cortical atrophy. | 1 | 0 | Freesurfer | White/gray matter volume |
| Schuster, 2013 | Focal thinning of the motor cortex mirrors clinical features of amyotrophic lateral sclerosis and their phenotypes: a neuroimaging study | Classical ALS, PLS, lower motor neuron variant | The upper motor neuron signs in the bulbar regions were associated with bilateral thinning within the bulbar segment on the motor cortex, and upper motor neuron signs in spinal regions were associated with thinning in the limb segment of the primary motor cortex. The site of disease onset (bulbar/lower limb) showed the most pronounced thinning in the corresponding part of the primary motor cortex. According to our analysis, dominant upper motor neuron patients exhibited the most distinct thinning followed by classical ALS patients. Pure lower motor neuron variants did not differ from healthy controls regarding cortical thickness. | 1 | 0 | Freesurfer | White/gray matter volume |
| Schuster, 2014 | Longitudinal course of cortical thickness decline in amyotrophic lateral sclerosis | Classical ALS, upper/lower motor neuron ALS variants | During MRI follow-up up to 15 months, study found a decline of cortical thickness in frontal, temporal, and parietal regions in ALS patients. The lower motor neuron ALS variants demonstrated the highest rates of cortical thinning in the precentral gyrus, the UMN-dominant subjects exhibited intermediate rates of atrophy, and the classical ALS patients exhibited no thinning of the primary motor cortex. | 1 | 1 | Freesurfer | White/gray matter volume |
| Schweitzer, 2015 | Quantitative susceptibility mapping of the motor cortex in amyotrophic lateral sclerosis and primary lateral sclerosis | ALS, PLS | Quantitatively, relative motor cortex susceptibility on QSM was found to be greater in patients with motor neuron disease than in healthy control patients. | 1 | 0 | Visual rating | T2 hyperintensity CST, QSM |
| Senda, 2017 | Structural MRI correlates of amyotrophic lateral sclerosis progression | Sporadic ALS | In comparison with healthy controls, ALS patients exhibited grey matter atrophy beyond the motor cortex and CST, especially in the frontotemporal lobes and basal ganglia. | 1 | 0 | SPM12 | White/gray matter volume |
| Senda, 2011 | Progressive and widespread brain damage in ALS: MRI voxel-based morphometry and diffusion tensor imaging study | ALS | ALS patients showed widespread volume decreases in gray matter, but volume changes in the white matter remained minimal and more distinct. | 1 | 1 | SPM5 | White/gray matter volume |
| Sha, 2012 | Frontotemporal dementia due to C9ORF72 mutations: clinical and imaging features | C9orf72+: behavioral variant FTD (bvFTD), FTD-MND, ALS. C9orf72-: bvFTD, FTD-MND, ALS | Increased thalamic atrophy in FTD and FTD-MND carriers than in noncarriers. | 1 | 0 | Diffeomorphic anatomical registration through exponentiated Lie algebra (DARTEL), SPM | White/gray matter volume |
| Shellikeri, 2019 | Speech network regional involvement in bulbar ALS: a multimodal structural MRI study | ALS | Structural changes were observed in the right oral and limb primary motor cortex and left pars triangularis, transverse temporal and posterior superior temporal gyurs in ALS. Bulbar motor dysfunction was associated with white matter abnormalities in the right oral primary motor cortex and left posterior superior temporal gyurs, and gray matter changes in bilateral transverse temporal. In contrast, symptom progression rate predicted gray and white matter alterations in bilateral pars opercularis (part of Broca's area). | 1 | 0 | Freesurfer | White/gray matter volume |
| Shen, 2018 | Brain Structural and Perfusion Signature of Amyotrophic Lateral Sclerosis With Varying Levels of Cognitive Deficit | ALS (with or without cognitive impairment), ALS-FTD | the ALS-FTD patients exhibited a similar pattern of gray matter loss with more significant changes in the left frontal and temporal lobe compared with the healthy controls or ALS with or without cognitive impairment. | 1 | 0 | Diffeomorphic anatomical registration through exponentiated Lie algebra (DARTEL), SPM | White/gray matter volume |
| Shen, 2018 | Monitoring Value of Multimodal Magnetic Resonance Imaging in Disease Progression of Amyotrophic Lateral Sclerosis: A Prospective Observational Study | ALS | Gray matter volume of the right precentral gyri, left postcentral gyri, and right thalami in both motor and extramotor areas at follow‐up was reduced compared to baseline. | 0 | 1 | SPM12 | White/gray matter volume |
| Shindo, 2014 | Neuropsychological study of amyotrophic lateral sclerosis and parkinsonism-dementia complex in Kii peninsula, Japan | Kii peninsula ALS | Brain MRI showed volume loss of the frontal and/or temporal lobes in ALS patients. | 0 | 0 | Visual rating | White/gray matter volume |
| Steinbach, 2020 | Applying the D50 disease progression model to gray and white matter pathology in amyotrophic lateral sclerosis | ALS | ALS patients showed widespread gray and white matter density reduction in the bilateral frontal and temporal lobes. | 1 | 0 | CAT12 VBM | White/gray matter volume |
| Takeda, 2007 | Memory deficits in amyotrophic lateral sclerosis patients with dementia and degeneration of the perforant pathway A clinicopathological study | ALS | One patient with high signal intensity in the dentate gyrus. | 0 | 0 | Visual rating | White/gray matter volume |
| Tavazzi, 2015 | Grey matter damage in progressive multiple sclerosis versus amyotrophic lateral sclerosis: a voxel-based morphometry MRI study | ALS | Compared to healthy controls, ALS patients showed gray matter volume reduction in selected frontal and temporal areas. Compared to ALS, progressive MS patients showed brain volume reductions in both deep and cortical gray matter areas. | 1 | 0 | FSL VBM | White/gray matter volume |
| Terada, 2016 | Correlation of frontal atrophy with behavioral changes in amyotrophic lateral sclerosis | ALS | In ALS patients, the volume of the right frontal cluster, but not the left medial frontal cluster, was smaller than that of healthy controls. | 1 | 0 | SPM8 | White/gray matter volume |
| Thivard, 2007 | Diffusion tensor imaging and voxel based morphometry study in amyotrophic lateral sclerosis: relationships with motor disability | ALS | ALS patients showed no changes in white matter but widespread volume loss in grey matter in several regions exhibiting mean diffusivity abnormalities. | 1 | 0 | SPM2 | White/gray matter volume |
| Thorns, 2013 | Extent of cortical involvement in amyotrophic lateral sclerosis--an analysis based on cortical thickness | ALS | ALS patients showed cortical thinning in the pre- and postcentral gyri bilaterally as well as in the superior and inferior parietal lobule, angular and supramarginal gyrus, insula, superior frontal, temporal, and occipital regions. | 1 | 0 | Brainvoyager QX 1.8-1.10 | White/gray matter volume |
| Thorpe, 1996 | Brain and spinal cord MRI in motor neuron disease | ALS | Symmetric areas of high signal within the CST were found in 9/11 patients on T2w contrast and in 8/11 on T2 or T2*w images of the spinal cord. High signal within the posterior limbs of the internal capsules was also found in 4 healthy controls. No controls had abnormalities within the spinal cord. Low signal within the motor cortex was found in 10/11 patients but was also observed in 6 healthy controls. | 1 | 0 | Visual rating | White/gray matter volume |
| Trojsi, 2020 | Frontotemporal degeneration in amyotrophic lateral sclerosis (ALS): a longitudinal MRI one-year study | ALS | During longitudinal follow-up (1 year), no changes in of gray matter volumes. | 1 | 1 | Diffeomorphic anatomical registration through exponentiated Lie algebra (DARTEL), SPM | White/gray matter volume |
| Turner, 2007 | Volumetric cortical loss in sporadic and familial amyotrophic lateral sclerosis | Sporadic ALS | ALS patients showed bilateral areas of atrophy, mostly confined to motor and pre-motor cortices. Cortical changes in the SOD gene homD90A group were more pronounced within the frontal lobes when both were compared with healthy controls | 1 | 0 | SPM2 | White/gray matter volume |
| Udaka, 1992 | MRI and SPECT findings in amyotrophic lateral sclerosis. Demonstration of upper motor neurone involvement by clinical neuroimaging | Sporadic ALS | T2w MRI showed high signal along the course of the CST in the internal capsule and cerebral peduncle in 4/21 patients (19%). | 1 | 0 | Visual rating | T2 hyperintensity CST |
| Van Mossevelde, 2016 | Clinical features of TBK1 carriers compared with C9orf72, GRN and non-mutation carriers in a Belgian cohort | ALS, FTD-ALS | MRI displayed widespread atrophy, both symmetric and asymmetric. | 0 | 0 | Visual rating | White/gray matter volume |
| Vazquez-Costa, 2019 | The width of the third ventricle associates with cognition and behaviour in motor neuron disease | Classical ALS, progressive muscular atrophy, PLS | MND patients showed larger width of the third ventricle than healthy controls. | 1 | 0 | Freesurfer | White/gray matter volume |
| Vazquez-Costa, 2018 | Brain signal intensity changes as biomarkers in amyotrophic lateral sclerosis | Sporadic and familial ALS | In healthy controls, Iron-related hypointensities in the motor cortex associated with age, but hyperintensities of the CST did not. In ALS patients, both hypointensities in the motor cortex and hyperintensities of the CST associated with clinical UMN impairment and bulbar onset. | 1 | 0 | Visual rating | T2 hyperintensities CST |
| Verstraete, 2010 | No evidence of microbleeds in ALS patients at 7 Tesla MRI | ALS | No microbleeds on T2*w contrasts were found in ALS patients. | 1 | 0 | Visual rating | White/gray matter volume |
| Verstraete, 2012 | Structural MRI reveals cortical thinning in amyotrophic lateral sclerosis | ALS | Study showed specific thinning in the primary motor cortex in patients with ALS compared with healthy controls. Surface area was reduced in the right inferior parietal region and volume was reduced in the right precentral gyrus (p=0.031). | 1 | 0 | Freesurfer | White/gray matter volume |
| Vibha, 2015 | Clinical profile of Monomelic Amyotrophy (MMA) and role of persistent viral infection | Monomelic amyotrophy | In the cervical spine MRI, 14% of the patients had cord hyperintensities, while 18% had cord volume loss. However, a most patients had a normal study (68%). | 1 | 0 | Visual rating | Spinal cord atrophy |
| Vincenti, 2019 | Primary progressive aphasia and the FTD-MND spectrum disorders: clinical, pathological, and neuroimaging correlates | FTLD-MND | MRI of non-fluent variant of PPA showed atrophy confined to the frontal and anterior temporal language cortices. Another patient group of seven cases that resembled patients with the semantic variant PPA exhibited selective atrophy of the temporal lobe and orbitofrontal cortex. | 1 | 0 | SPM12 | White/gray matter volume |
| Walhout, 2015 | Cortical thickness in ALS: towards a marker for upper motor neuron involvement | ALS, UMN ALS, LMN ALS | Cortical thickness of the precentral gyrus was reduced in ALS but not in patients with an LMN phenotype, as compared to healthy controls. Compared to patients with ALS, patients with a UMN phenotype showed an even lower precentral gyrus cortical thickness. Longitudinal analysis showed a decrease of cortical thickness in the left temporal lobe of ALS patients. | 1 | 1 | Freesurfer | White/gray matter volume |
| Waragai, 1997 | MRI and clinical features in amyotrophic lateral sclerosis | ALS | High signal was seen in the intracranial CST in 16/21 ALS patients (76%) on T2w and in 10/21 (48%) on PDw images. Low signal intensity was seen in the motor cortex in 12/21 ALS patients (57%). High signal in the anterolateral column of the spinal cord on T1w images was seen in 14, and high signal in the lateral CST on T2w images was seen in 7/21 ALS patients. High signal intensity was seen in the intracranial CST on T1w images in 5 ALS patients who showed pronounced upper motor neurone signs including spastic paraparesis, but not in healthy controls. | 1 | 1 | Visual rating | T2 hyperintensities CST |
| Waragai, 1997 | High signal intensity on T1 weighted MRI of the anterolateral column of the spinal cord in amyotrophic lateral sclerosis | ALS | In all the control patients, the signal intensity of the posterior column was equal/slightly hypointense compared with the anterolateral column of the cervical spinal cord on T1w contrast. 8/14 patients with ALS (57%) showed high signal intensity in the anterolateral column of the spinal cord on T1w MRI. 2/14 patients (14%) also disclosed high signal intensity of the intracranial CSTients. 2/14 patients showed no abnormal findings on MRI. | 1 | 0 | Visual rating | T2 hyperintensities CST |
| Welton, 2019 | Diffusion kurtosis and quantitative susceptibility mapping MRI are sensitive to structural abnormalities in amyotrophic lateral sclerosis | ALS | Within the ALS group, study found associations between motor cortex volume, apparent diffusion and disease duration. | 1 | 0 | Freesurfer | White/gray matter volume |
| Westeneng, 2015 | Subcortical structures in amyotrophic lateral sclerosis | ALS | At baseline, ALS patients exhibited reduced hippocampal volumes and larger inferior lateral ventricles compared to healthy controls. Longitudinal analyses demonstrated a significant decrease in volume of the right cornu ammonis 2/3 and 4/dentate gyrus and left presubiculum and an increase in the ventricular volume in the lateral, 3rd as well as 4th ventricles. | 1 | 1 | Freesurfer | White/gray matter volume |
| Westeneng, 2016 | Widespread structural brain involvement in ALS is not limited to the C9orf72 repeat expansion | ALS (C9orf72+ or -) | C9orf72+ ALS patients showed cortical thinning outside the precentral gyrus, extending to the bilateral pars opercularis, fusiform, lingual, isthmus-cingulate, and superior parietal cortex, and smaller volumes of the right hippocampus and bilateral thalamus, and reduced white matter volumes of the inferior and superior longitudinal fasciculus compared with C9orf72- patients. Among 128 C9orf72- patients, we detected a subgroup of 27 (21%) with a neuroimaging phenotype congruent to C9orf72+ patients, while 101 C9orf72- patients (79%) showed cortical thinning restricted to the primary motor cortex. | 0 | 0 | Freesurfer | White/gray matter volume |
| Whitwell, 2006 | Patterns of atrophy in pathologically confirmed FTLD with and without motor neuron degeneration | FTLD-MND | Study found atrophy patterns distinct and different from each other: A localized pattern of frontal lobe atrophy was found in FTLD-MND with a more widespread pattern of volume loss affecting the frontal and temporal lobes in FTLD-U. | 0 | 0 | SPM2 | White/gray matter volume |
| Wirth, 2019 | Value of fluid-attenuated inversion recovery MRI data analyzed by the lesion segmentation toolbox in amyotrophic lateral sclerosis | ALS | Both ALS patients as well as healthy controls exhibited T2w-FLAIR alterations. ALS patients showed higher total lesion number compared to healthy controls. These signal alterations were mostly detected in the superior and posterior corona radiata, anterior capsula interna, and posterior thalamic radiation. | 1 | 0 | lesion segmentation toolbox | T2 hyperintensities |
| Wirth, 2018 | Combinatory Biomarker Use of Cortical Thickness, MUNIX, and ALSFRS-R at Baseline and in Longitudinal Courses of Individual Patients With Amyotrophic Lateral Sclerosis | Limb onset classical ALS | MRI analysis showed individually variable states of cortical thinning, which was most pronounced in the ventral section of the precentral cortex. | 1 | 0 | Freesurfer | White/gray matter volume |
| Wu, 2006 | Comparison of diffusion-weighted MR imaging and T2-weighted MR imaging in patients with amyotrophic lateral sclerosis | ALS | T2w images showed high signal intensity in the CST in 11/12 patients with ALS (92%) and 8/12 healthy controls (67%). The PDw images exhibited the high signal CST in 5/12 patients with ALS (42%), but not in any of the healthy controls. | 1 | 0 | Visual rating | T2 hyperintensities CST |
| Yagishita, 1994 | Location of the corticospinal tract in the internal capsule at MR imaging | ALS | In the posterior internal capsule, the brains from healthy controls demonstrated a pale area with large axons and thick myelin sheaths. In 5 ALS patients, the brain histopathology showed CST degeneration in the same region. MRI demonstrated T2w hyperintensity that represented degeneration of the CST in 5/35 ALS patients. In the same region, hyperintense foci were found in the control subjects. | 1 | 0 | Visual rating | T2 hyperintensities CST |
| York, 2014 | Action verb comprehension in amyotrophic lateral sclerosis and Parkinson's disease | ALS | ALS patients exhibited gray matter volume loss compared to controls with Parkinson's disease in several regions of the frontal lobe bilaterally. | 0 | 0 | ANTS, SPM8 | White/gray matter volume |
| Yu, 2014 | Increased iron level in motor cortex of amyotrophic lateral sclerosis patients: an in vivo MR study | ALS | Study found that SWI phase shift values were higher in the motor cortex of ALS patients, indicating increased local iron levels. There was no difference in the other nuclei including the red nucleus, substantia nigra, globus pallidus, putamen, and the head of the caudate nucleus. | 1 | 0 | Visual rating | Susceptibility changes in motor cortex |
| Yunusova, 2019 | Frontal Anatomical Correlates of Cognitive and Speech Motor Deficits in Amyotrophic Lateral Sclerosis | ALS | Primary motor and somatosensory cortex as well as the superior frontal regions were associated with supplementary and presupplementary motor areas were prominent, suggesting that the worsening performance on each task was associated with the volumetric changes in the regions supporting various motor aspects of the task execution. | 1 | 0 | SABRE | White/gray matter volume |
| Zhang, 2014 | Regional alterations in cortical thickness and white matter integrity in amyotrophic lateral sclerosis | ALS | in ALS patients, cortical thinning was observed in motor-related cortices (i.e., bilateral precentral gyri, dorsal premotor cortices, and left supplementary motor area) as well as prefrontal and occipito-parietal regions. | 1 | 0 | CIVET MRI analysis pipeline | White/gray matter volume |
| Zhang, 2003 | The diagnostic utility of FLAIR imaging in clinically verified amyotrophic lateral sclerosis | ALS | Subcortical white matter hyperintensities on T2w-FLAIR and the dark line along the posterior rim of the precentral gyri were found more commonly in ALS patients compared to healthy controls. T2w-FLAIR hyperintensities in the CST was found in both ALS and normal controls. | 1 | 0 | Visual rating | T2 hyperintensities CST |
| Zhang, 2014 | Side of limb-onset predicts laterality of gray matter loss in amyotrophic lateral sclerosis | ALS (right or left limb involvement) | Study found global brain atrophy and gray matter losses in frontal and parietal areas in each patient group as well as left gray matter losses in the total cohort. | 1 | 0 | FSL VBM | White/gray matter volume |
| Zhang, 2017 | Occipital cortical gyrification reductions associate with decreased functional connectivity in amyotrophic lateral sclerosis | ALS | ALS patients had reduced local gyrification index in right occipital cortex. | 1 | 0 | Freesurfer | White/gray matter volume |
| Zhang, 2019 | Abnormal topological organization of structural covariance networks in amyotrophic lateral sclerosis | ALS | ALS patients showed decreased nodal degree and betweenness in the gyrus rectus and/or Heschl gyrus as well as increased betweenness in the supplementary motor area, triangular part of the inferior frontal gyrus, supramarginal gyrus and posterior cingulate cortex. | 1 | 0 | Automated Anatomical Labeling Atlas | White/gray matter volume |
| Zhu, 2015 | ALFF Value in Right Parahippocampal Gyrus Acts as a Potential Marker Monitoring Amyotrophic Lateral Sclerosis Progression: a Neuropsychological, Voxel-Based Morphometry, and Resting-State Functional MRI Study | ALS | In ALS patients showed decreased gray matter volume in the bilateral precentral gyri. | 1 | 0 | SPM8 | White/gray matter volume |

**Supplementary reference lists**

**Supplementary reference list from human studies**

In alphabetical order

1 Abdulla, S. et al. Hippocampal degeneration in patients with amyotrophic lateral sclerosis. Neurobiology of Aging 35, 2639-2645 (2014).

2 Abe, K., Fujimura, H., Kobayashi, Y., Fujita, N. & Yanagihara, T. Degeneration of the pyramidal tracts in patients with amyotrophic lateral sclerosis. A premortem and postmortem magnetic resonance imaging study. Journal of Neuroimaging 7, 208-212 (1997).

3 Abe, K. et al. Single-photon emission computed tomographic investigation of patients with motor neuron disease. Neurology 43, 1569-1573 (1993).

4 Abrahams, S. et al. Executive dysfunction in amyotrophic lateral sclerosis (ALS); a functional and structural MRI study. Neuroimage 13, S765-S765 (2001).

5 Abrahams, S. et al. Frontotemporal white matter changes in amyotrophic lateral sclerosis. Journal of Neurology 252, 321-331 (2005).

6 Acosta-Cabronero, J. et al. Quantitative Susceptibility MRI to Detect Brain Iron in Amyotrophic Lateral Sclerosis. Radiology 289, 195-203 (2018).

7 Adachi, Y. et al. Usefulness of SWI for the Detection of Iron in the Motor Cortex in Amyotrophic Lateral Sclerosis. Journal of Neuroimaging 25, 443-451 (2015).

8 Agosta, F. et al. Structural brain correlates of cognitive and behavioral impairment in MND. Human Brain Mapping 37, 1614-1626 (2016).

9 Agosta, F. et al. Structural and functional brain signatures of C9orf72 in motor neuron disease. Neurobiology of Aging 57, 206-219 (2017).

10 Agosta, F. et al. MRI signatures of the frontotemporal lobar degeneration continuum. Human Brain Mapping 36, 2602-2614 (2015).

11 Agosta, F. et al. Longitudinal assessment of grey matter contraction in amyotrophic lateral sclerosis: A tensor based morphometry study. Amyotrophic Lateral Sclerosis 10, 168-174 (2009).

12 Agosta, F. et al. Voxel-based morphometry study of brain volumetry and diffusivity in amyotrophic lateral sclerosis patients with mild disability. Human Brain Mapping 28, 1430-1438 (2007).

13 Agosta, F. et al. Survival prediction models in motor neuron disease. European Journal of Neurology 26, 1143-1152 (2019).

14 Agosta, F. et al. The cortical signature of amyotrophic lateral sclerosis. PLoS ONE [Electronic Resource] 7, e42816 (2012).

15 Alruwaili, A. R. et al. A combined tract-based spatial statistics and voxel-based morphometry study of the first MRI scan after diagnosis of amyotrophic lateral sclerosis with subgroup analysis. Journal of Neuroradiology. Journal de Neuroradiologie 45, 41-48 (2018).

16 Ambikairajah, A. et al. A visual MRI atrophy rating scale for the amyotrophic lateral sclerosis-frontotemporal dementia continuum. Amyotrophic Lateral sclerosis & Frontotemporal Degeneration 15, 226-234 (2014).

17 Andreadou, E., Sgouropoulos, P., Varelas, P., Gouliamos, A. & Papageorgiou, C. Subcortical frontal lesions on MRI in patients with motor neurone disease. Neuroradiology 40, 298-302 (1998).

18 Ash, S. et al. Narrative discourse deficits in amyotrophic lateral sclerosis. Neurology 83, 520-528 (2014).

19 Bae, J. S. et al. Dissociation of Structural and Functional Integrities of the Motor System in Amyotrophic Lateral Sclerosis and Behavioral-Variant Frontotemporal Dementia. Journal of Clinical Neurology 12, 209-217 (2016).

20 Basak, M. et al. Magnetic resonance imaging in amyotrophic lateral sclerosis. Acta Neurologica Scandinavica 105, 395-399 (2002).

21 Bede, P. et al. The Neuroimaging Signature of the C9orf72 Hexanucleotide Repeat in Amyotrophic Lateral Sclerosis - A Multimodal MRI Study. Neurology 80, 2 (2013).

22 Bede, P. et al. Grey Matter Correlates of Clinical Variables in Amyotrophic Lateral Sclerosis - A Neuroimaging Study of ALS Motor Phenotype Heterogeneity and Cortical Focality. Neurology 80, 1 (2013).

23 Bede, P. et al. Basal ganglia involvement in amyotrophic lateral sclerosis. Neurology 81, 2107-2115 (2013).

24 Bede, P. & Hardiman, O. Longitudinal structural changes in ALS: a three time-point imaging study of white and gray matter degeneration. Amyotrophic Lateral sclerosis & Frontotemporal Degeneration 19, 232-241 (2018).

25 Bede, P. et al. Connectivity-based characterisation of subcortical grey matter pathology in frontotemporal dementia and ALS: a multimodal neuroimaging study. Brain Imaging & Behavior 12, 1696-1707 (2018).

26 Bocchetta, M. et al. Thalamic atrophy in frontotemporal dementia — Not just a C9orf72 problem. NeuroImage: Clinical 18, 675-681 (2018).

27 Bocchetta, M. et al. Thalamic nuclei in frontotemporal dementia: Mediodorsal nucleus involvement is universal but pulvinar atrophy is unique to C9orf72. Human Brain Mapping 41, 1006-1016 (2020).

28 Boll, M. C., Marrufo Melendez, O. R., Rios, C., Maciel Zenil, J. & de Alba, Y. Is the Hypointensity in Motor Cortex the Hallmark of Amyotrophic Lateral Sclerosis? Canadian Journal of Neurological Sciences 46, 166-173 (2019).

29 Branco, L. M. T. et al. Brain signature of mild stages of cognitive and behavioral impairment in amyotrophic lateral sclerosis. Psychiatry Research: Neuroimaging 272, 58-64 (2018).

30 Buhour, M. S. et al. Voxel-based mapping of grey matter volume and glucose metabolism profiles in amyotrophic lateral sclerosis. EJNMMI Research 7, 21 (2017).

31 Butman, J. A. & Floeter, M. K. Decreased thickness of primary motor cortex in primary lateral sclerosis. Ajnr: American Journal of Neuroradiology 28, 87-91 (2007).

32 Canu, E. et al. The topography of brain microstructural damage in amyotrophic lateral sclerosis assessed using diffusion tensor MR imaging. Ajnr: American Journal of Neuroradiology 32, 1307-1314 (2011).

33 Cardenas-Blanco, A. et al. Structural and diffusion imaging versus clinical assessment to monitor amyotrophic lateral sclerosis. NeuroImage Clinical 11, 408-414 (2016).

34 Carella, F., Grisoli, M., Savoiardo, M. & Testa, D. Magnetic resonance signal abnormalities along the pyramidal tracts in amyotrophic lateral sclerosis. Italian Journal of Neurological Sciences 16, 511-515 (1995).

35 Cerami, C. et al. Emotional empathy in amyotrophic lateral sclerosis: a behavioural and voxel-based morphometry study. Amyotrophic Lateral sclerosis & Frontotemporal Degeneration 15, 21-29 (2014).

36 Cervo, A. et al. The combined use of conventional MRI and MR spectroscopic imaging increases the diagnostic accuracy in amyotrophic lateral sclerosis. European Journal of Radiology 84, 151-157 (2015).

37 Chang, J. L. et al. A voxel-based morphometry study of patterns of brain atrophy in ALS and ALS/FTLD. Neurology 65, 75-80 (2005).

38 Chapman, M. C. et al. Corpus callosum area in amyotrophic lateral sclerosis. Amyotrophic Lateral Sclerosis 13, 589-591 (2012).

39 Charil, A. et al. Structural and metabolic changes in the brain of patients with upper motor neuron disorders: a multiparametric MRI study. Amyotrophic Lateral Sclerosis 10, 269-279 (2009).

40 Chen, J., Kostenko, V., Pioro, E. P. & Trapp, B. D. MR Imaging-based Estimation of Upper Motor Neuron Density in Patients with Amyotrophic Lateral Sclerosis: A Feasibility Study. Radiology 287, 955-964 (2018).

41 Chen, Z. Y., Liu, M. Q. & Ma, L. Cortical Thinning Pattern of Bulbar- and Spinal-onset Amyotrophic Lateral Sclerosis: a Surface-based Morphometry Study. Chinese Medical Sciences Journal 33, 100-106 (2018).

42 Cheung, G. et al. Amyotrophic lateral sclerosis: correlation of clinical and MR imaging findings. Radiology 194, 263-270 (1995).

43 Christidi, F. et al. Hippocampal pathology in amyotrophic lateral sclerosis: selective vulnerability of subfields and their associated projections. Neurobiology of Aging 84, 178-188 (2019).

44 Christidi, F. et al. Gray matter and white matter changes in non-demented amyotrophic lateral sclerosis patients with or without cognitive impairment: A combined voxel-based morphometry and tract-based spatial statistics whole-brain analysis. Brain Imaging & Behavior 12, 547-563 (2018).

45 Christidi, F. et al. Motor and extra-motor gray matter integrity may underlie neurophysiologic parameters of motor function in amyotrophic lateral sclerosis: a combined voxel-based morphometry and transcranial stimulation study. Brain Imaging & Behavior 12, 1730-1741 (2018).

46 Clark, M. G. et al. Loss of functional connectivity is an early imaging marker in primary lateral sclerosis. Amyotrophic Lateral sclerosis & Frontotemporal Degeneration 19, 562-569 (2018).

47 Cohen-Adad, J. et al. Involvement of spinal sensory pathway in ALS and specificity of cord atrophy to lower motor neuron degeneration. Amyotrophic Lateral sclerosis & Frontotemporal Degeneration 14, 30-38 (2013).

48 Consonni, M. et al. Cognitive Syndromes and C9orf72 Mutation Are Not Related to Cerebellar Degeneration in Amyotrophic Lateral Sclerosis. Frontiers in Neuroscience 13 (2019).

49 Consonni, M., Cappa, S. F., Dalla Bella, E., Contarino, V. E. & Lauria, G. Cortical correlates of behavioural change in amyotrophic lateral sclerosis. Journal of Neurology, Neurosurgery & Psychiatry 90, 380-386 (2019).

50 Consonni, M. et al. Cortical markers of cognitive syndromes in amyotrophic lateral sclerosis. NeuroImage Clinical 19, 675-682 (2018).

51 Contarino, V. E. et al. Toward a marker of upper motor neuron impairment in amyotrophic lateral sclerosis: A fully automatic investigation of the magnetic susceptibility in the precentral cortex. European Journal of Radiology 124, 108815 (2020).

52 Coon, E. A., Sorenson, E. J., Whitwell, J. L., Knopman, D. S. & Josephs, K. A. Predicting survival in frontotemporal dementia with motor neuron disease. Neurology 76, 1886-1893 (2011).

53 Coon, E. A., Whitwell, J. L., Parisi, J. E., Dickson, D. W. & Josephs, K. A. Right temporal variant frontotemporal dementia with motor neuron disease. Journal of Clinical Neuroscience 19, 85-91 (2012).

54 Cosottini, M. et al. Mapping cortical degeneration in ALS with magnetization transfer ratio and voxel-based morphometry. PLoS ONE [Electronic Resource] 8, e68279 (2013).

55 Cosottini, M. et al. High-Resolution 7T MR Imaging of the Motor Cortex in Amyotrophic Lateral Sclerosis. Ajnr: American Journal of Neuroradiology 37, 455-461 (2016).

56 Cosottini, M. et al. Structural and functional evaluation of cortical motor areas in Amyotrophic Lateral Sclerosis. Experimental Neurology 234, 169-180 (2012).

57 Costagli, M. et al. Magnetic susceptibility in the deep layers of the primary motor cortex in Amyotrophic Lateral Sclerosis. NeuroImage Clinical 12, 965-969 (2016).

58 Crespi, C. et al. Multimodal MRI quantification of the common neurostructural bases within the FTD-ALS continuum. Neurobiology of Aging 62, 95-104 (2018).

59 da Rocha, A. J., Maia, A. C., Jr., Nogueira, R. G. & Lederman, H. M. Magnetic resonance findings in amyotrophic lateral sclerosis using a spin echo magnetization transfer sequence. Preliminary report. Arquivos de Neuro-Psiquiatria 57, 912-915 (1999).

60 da Rocha, A. J. et al. Detection of corticospinal tract compromise in amyotrophic lateral sclerosis with brain MR imaging: relevance of the T1-weighted spin-echo magnetization transfer contrast sequence. Ajnr: American Journal of Neuroradiology 25, 1509-1515 (2004).

61 d'Ambrosio, A. et al. Frontotemporal cortical thinning in amyotrophic lateral sclerosis. Ajnr: American Journal of Neuroradiology 35, 304-310 (2014).

62 de Albuguergues, M. et al. Multimodal Longitudinal MRI Study in Amyotrophic Lateral Sclerosis (ALS). Neurology 86, 2 (2016).

63 de Albuquerque, M. et al. MRI Texture Analysis Reveals Deep Gray Nuclei Damage in Amyotrophic Lateral Sclerosis. Journal of Neuroimaging 26, 201-206 (2016).

64 de Albuquerque, M. et al. Longitudinal evaluation of cerebral and spinal cord damage in Amyotrophic Lateral Sclerosis. NeuroImage Clinical 14, 269-276 (2017).

65 De Marco, M. et al. Morphometric correlates of dysarthric deficit in amyotrophic lateral sclerosis. Amyotrophic Lateral sclerosis & Frontotemporal Degeneration 16, 464-472 (2015).

66 De Reuck, J. et al. Topographic distribution of brain iron deposition and small cerebrovascular lesions in amyotrophic lateral sclerosis and in frontotemporal lobar degeneration: a post-mortem 7.0-tesla magnetic resonance imaging study with neuropathological correlates. Acta Neurologica Belgica 117, 873-878 (2017).

67 De Reuck, J. L. et al. Iron deposits in post-mortem brains of patients with neurodegenerative and cerebrovascular diseases: a semi-quantitative 7.0 T magnetic resonance imaging study. European Journal of Neurology 21, 1026-1031 (2014).

68 Devenney, E. et al. Frontotemporal dementia associated with the C9ORF72 mutation: a unique clinical profile. JAMA Neurology 71, 331-339 (2014).

69 Devenney, E. M. et al. The neural correlates and clinical characteristics of psychosis in the frontotemporal dementia continuum and the C9orf72 expansion. NeuroImage Clinical 13, 439-445 (2017).

70 Devine, M. S. et al. Exposing asymmetric gray matter vulnerability in amyotrophic lateral sclerosis. NeuroImage Clinical 7, 782-787 (2015).

71 Ding, X. Q. et al. Value of quantitative analysis of routine clinical MRI sequences in ALS. Amyotrophic Lateral Sclerosis 12, 406-413 (2011).

72 Donatelli, G. et al. MRI cortical feature of bulbar impairment in patients with amyotrophic lateral sclerosis. NeuroImage Clinical 24, 101934 (2019).

73 Donatelli, G. et al. Semiautomated Evaluation of the Primary Motor Cortex in Patients with Amyotrophic Lateral Sclerosis at 3T. Ajnr: American Journal of Neuroradiology 39, 63-69 (2018).

74 Duning, T. et al. G-CSF prevents the progression of structural disintegration of white matter tracts in amyotrophic lateral sclerosis: a pilot trial. PLoS ONE [Electronic Resource] 6, e17770 (2011).

75 El Mendili, M. M. et al. Multi-parametric spinal cord MRI as potential progression marker in amyotrophic lateral sclerosis. PLoS ONE [Electronic Resource] 9, e95516 (2014).

76 Ellis, C. M. et al. Volumetric analysis reveals corticospinal tract degeneration and extramotor involvement in ALS. Neurology 57, 1571-1578 (2001).

77 Endo, H. et al. Low signal intensity in motor cortex on susceptibility-weighted MR imaging is correlated with clinical signs of amyotrophic lateral sclerosis: a pilot study. Journal of Neurology 265, 552-561 (2018).

78 Evans, J. et al. Impaired cognitive flexibility in amyotrophic lateral sclerosis. Cognitive & Behavioral Neurology 28, 17-26 (2015).

79 Fabes, J. et al. Quantitative FLAIR MRI in Amyotrophic Lateral Sclerosis. Academic Radiology 24, 1187-1194 (2017).

80 Feron, M. et al. Extrapyramidal deficits in ALS: a combined biomechanical and neuroimaging study. Journal of Neurology 265, 2125-2136 (2018).

81 Ferraro, P. M. et al. Multimodal structural MRI in the diagnosis of motor neuron diseases. NeuroImage Clinical 16, 240-247 (2017).

82 Ferraro, P. M. et al. Perfusion alterations converge with patterns of pathological spread in transactive response DNA-binding protein 43 proteinopathies. Neurobiology of Aging 68, 85-92 (2018).

83 Finegan, E. et al. The clinical and radiological profile of primary lateral sclerosis: a population-based study. Journal of Neurology 266, 2718-2733 (2019).

84 Finegan, E. et al. Widespread subcortical grey matter degeneration in primary lateral sclerosis: a multimodal imaging study with genetic profiling. NeuroImage Clinical 24, 102089 (2019).

85 Floeter, M. K. et al. Longitudinal imaging in C9orf72 mutation carriers: Relationship to phenotype. NeuroImage Clinical 12, 1035-1043 (2016).

86 Frank, B., Haas, J., Heinze, H. J., Stark, E. & Munte, T. F. Relation of neuropsychological and magnetic resonance findings in amyotrophic lateral sclerosis: evidence for subgroups. Clinical Neurology & Neurosurgery 99, 79-86 (1997).

87 Geevasinga, N. et al. Brain functional connectome abnormalities in amyotrophic lateral sclerosis are associated with disability and cortical hyperexcitability. European Journal of Neurology 24, 1507-1517 (2017).

88 Goodin, D. S., Rowley, H. A. & Olney, R. K. Magnetic resonance imaging in amyotrophic lateral sclerosis. Annals of Neurology 23, 418-420 (1988).

89 Graham, J. M. et al. Diffusion tensor imaging for the assessment of upper motor neuron integrity in ALS. Neurology 63, 2111-2119 (2004).

90 Grieve, S. M. et al. Potential structural and functional biomarkers of upper motor neuron dysfunction in ALS. Amyotrophic Lateral sclerosis & Frontotemporal Degeneration 17, 85-92 (2015).

91 Grossman, M. et al. Impaired action knowledge in amyotrophic lateral sclerosis. Neurology 71, 1396-1401 (2008).

92 Gupta, A., Nguyen, T. B., Chakraborty, S. & Bourque, P. R. Accuracy of Conventional MRI in ALS. Canadian Journal of Neurological Sciences 41, 53-57 (2014).

93 Hartung, V. et al. Voxel-based MRI intensitometry reveals extent of cerebral white matter pathology in amyotrophic lateral sclerosis. PLoS ONE [Electronic Resource] 9, e104894 (2014).

94 Hecht, M. J., Fellner, C., Schmid, A., Neundorfer, B. & Fellner, F. A. Cortical T2 signal shortening in amyotrophic lateral sclerosis is not due to iron deposits. Neuroradiology 47, 805-808 (2005).

95 Hecht, M. J. et al. MRI-FLAIR images of the head show corticospinal tract alterations in ALS patients more frequently than T2-, T1- and proton-density-weighted images. Journal of the Neurological Sciences 186, 37-44 (2001).

96 Hecht, M. J. et al. Hyperintense and hypointense MRI signals of the precentral gyrus and corticospinal tract in ALS: a follow-up examination including FLAIR images. Journal of the Neurological Sciences 199, 59-65 (2002).

97 Hofmann, E., Ochs, G., Pelzl, A. & Warmuth-Metz, M. The corticospinal tract in amyotrophic lateral sclerosis: an MRI study. Neuroradiology 40, 71-75 (1998).

98 Ignjatovic, A., Stevic, Z., Lavrnic, S., Dakovic, M. & Bacic, G. Brain iron MRI: a biomarker for amyotrophic lateral sclerosis. Journal of Magnetic Resonance Imaging 38, 1472-1479 (2013).

99 Irwin, D. J. et al. Cognitive decline and reduced survival in C9orf72 expansion frontotemporal degeneration and amyotrophic lateral sclerosis. Journal of Neurology, Neurosurgery & Psychiatry 84, 163-169 (2013).

100 Ishikawa, K., Nagura, H., Yokota, T. & Yamanouchi, H. Signal loss in the motor cortex on magnetic resonance images in amyotrophic lateral sclerosis. Annals of Neurology 33, 218-222 (1993).

101 Iwasaki, Y., Kinoshita, M., Ikeda, K. & Takamiya, K. Central nervous system magnetic resonance imaging findings in amyotrophic lateral sclerosis. European Archives of Psychiatry & Neurological Sciences 239, 125-126 (1989).

102 Iwasaki, Y., Kinoshita, M., Ikeda, K., Takamiya, K. & Shiojima, T. MRI in patients with amyotrophic lateral sclerosis: correlation with clinical features. International Journal of Neuroscience 59, 253-258 (1991).

103 Jin, J. et al. Dominant Heterogeneity of Upper and Lower Motor Neuron Degeneration to Motor Manifestation of Involved Region in Amyotrophic Lateral Sclerosis. Scientific Reports 9, 20059 (2019).

104 Jin, J., Hu, F., Zhang, Q., Jia, R. & Dang, J. Hyperintensity of the corticospinal tract on FLAIR: A simple and sensitive objective upper motor neuron degeneration marker in clinically verified amyotrophic lateral sclerosis. Journal of the Neurological Sciences 367, 177-183 (2016).

105 Josephs, K. A. et al. Corticospinal tract degeneration associated with TDP-43 type C pathology and semantic dementia. Brain 136, 455-470 (2013).

106 Kamminga, J. et al. Syntactic comprehension deficits across the FTD-ALS continuum. Neurobiology of Aging 41, 11-18 (2016).

107 Kassubek, J. et al. Global brain atrophy and corticospinal tract alterations in ALS, as investigated by voxel-based morphometry of 3-D MRI. Amyotrophic Lateral Sclerosis & Other Motor Neuron Disorders 6, 213-220 (2005).

108 Kato, S., Hayashi, H. & Yagishita, A. Involvement of the frontotemporal lobe and limbic system in amyotrophic lateral sclerosis: as assessed by serial computed tomography and magnetic resonance imaging. Journal of the Neurological Sciences 116, 52-58 (1993).

109 Keller, J. et al. Quantitative brain MR imaging in amyotrophic lateral sclerosis. Magma 24, 67-76 (2011).

110 Kiernan, J. A. & Hudson, A. J. Frontal lobe atrophy in motor neuron diseases. Brain 117, 747-757 (1994).

111 Kim, H. J. et al. Relationship between Clinical Parameters and Brain Structure in Sporadic Amyotrophic Lateral Sclerosis Patients According to Onset Type: A Voxel-Based Morphometric Study. PLoS ONE [Electronic Resource] 12, e0168424 (2017).

112 Kim, H. J. et al. Structural explanation of poor prognosis of amyotrophic lateral sclerosis in the non-demented state. European Journal of Neurology 24, 122-129 (2017).

113 Koike, Y. et al. Apparent diffusion coefficients distinguish amyotrophic lateral sclerosis from cervical spondylotic myelopathy. Clinical Neurology & Neurosurgery 132, 33-36 (2015).

114 Konno, T. et al. Japanese amyotrophic lateral sclerosis patients with GGGGCC hexanucleotide repeat expansion in C9ORF72. Journal of Neurology, Neurosurgery and Psychiatry 84, 398-401 (2013).

115 Kono, Y. et al. Clinical characteristics associated with corticospinal tract hyperintensity on magnetic resonance imaging in patients with amyotrophic lateral sclerosis. Clinical Neurology & Neurosurgery 127, 1-4 (2014).

116 Kuipers-Upmeijer, J., de Jager, A. E., Hew, J. M., Snoek, J. W. & van Weerden, T. W. Primary lateral sclerosis: clinical, neurophysiological, and magnetic resonance findings. Journal of Neurology, Neurosurgery & Psychiatry 71, 615-620 (2001).

117 Kwan, J. Y. et al. Iron accumulation in deep cortical layers accounts for MRI signal abnormalities in ALS: correlating 7 tesla MRI and pathology. PLoS ONE [Electronic Resource] 7, e35241 (2012).

118 Kwan, J. Y., Meoded, A., Danielian, L. E., Wu, T. & Floeter, M. K. Structural imaging differences and longitudinal changes in primary lateral sclerosis and amyotrophic lateral sclerosis. NeuroImage Clinical 2, 151-160 (2012).

119 Le Ber, I. et al. Phenotype variability in progranulin mutation carriers: a clinical, neuropsychological, imaging and genetic study. Brain 131, 732-746 (2008).

120 Lee, J. Y. et al. Quantitative susceptibility mapping of the motor cortex: a comparison of susceptibility among patients with amyotrophic lateral sclerosis, cerebrovascular disease, and healthy controls. Neuroradiology 59, 1213-1222 (2017).

121 Lee, S. E. et al. Altered network connectivity in frontotemporal dementia with C9orf72 hexanucleotide repeat expansion. Brain 137, 3047-3060 (2014).

122 Lee, S. E. et al. Network degeneration and dysfunction in presymptomatic C9ORF72 expansion carriers. NeuroImage Clinical 14, 286-297 (2017).

123 Leslie, F. V. et al. Semantic deficits in amyotrophic lateral sclerosis. Amyotrophic Lateral sclerosis & Frontotemporal Degeneration 16, 46-53 (2015).

124 Libon, D. J. et al. Deficits in concept formation in amyotrophic lateral sclerosis. Neuropsychology 26, 422-429 (2012).

125 Lillo, P. et al. Grey and white matter changes across the amyotrophic lateral sclerosis-frontotemporal dementia continuum. PLoS ONE [Electronic Resource] 7, e43993 (2012).

126 Luis, M. L., Hormigo, A., Mauricio, C., Alves, M. M. & Serrao, R. Magnetic resonance imaging in motor neuron disease. Journal of Neurology 237, 471-474 (1990).

127 Machts, J. et al. Prefrontal cortical thickness in motor neuron disease. NeuroImage Clinical 18, 648-655 (2018).

128 Machts, J. et al. Basal ganglia pathology in ALS is associated with neuropsychological deficits. Neurology 85, 1301-1309 (2015).

129 Machts, J. et al. Global Hippocampal Volume Reductions and Local CA1 Shape Deformations in Amyotrophic Lateral Sclerosis. Frontiers in neurology [electronic resource]. 9, 565 (2018).

130 Mahoney, C. J. et al. Frontotemporal dementia with the C9ORF72 hexanucleotide repeat expansion: clinical, neuroanatomical and neuropathological features. Brain 135, 736-750 (2012).

131 Mahoney, C. J. et al. Longitudinal neuroimaging and neuropsychological profiles of frontotemporal dementia with C9ORF72 expansions. Alzheimer's Research & Therapy 4, 41 (2012).

132 McCluskey, L. et al. ALS-Plus syndrome: non-pyramidal features in a large ALS cohort. Journal of the Neurological Sciences 345, 118-124 (2014).

133 McMillan, C. T. et al. C9orf72 promoter hypermethylation is neuroprotective: Neuroimaging and neuropathologic evidence. Neurology 84, 1622-1630 (2015).

134 Menke, R. A. L., Proudfoot, M., Talbot, K. & Turner, M. R. The two-year progression of structural and functional cerebral MRI in amyotrophic lateral sclerosis. NeuroImage Clinical 17, 953-961 (2018).

135 Meoded, A. et al. Imaging findings associated with cognitive performance in primary lateral sclerosis and amyotrophic lateral sclerosis. Dementia and Geriatric Cognitive Disorders Extra 3, 233-250 (2013).

136 Mezzapesa, D. M. et al. Whole-brain and regional brain atrophy in amyotrophic lateral sclerosis. Ajnr: American Journal of Neuroradiology 28, 255-259 (2007).

137 Mezzapesa, D. M. et al. Cortical thinning and clinical heterogeneity in amyotrophic lateral sclerosis. PLoS ONE [Electronic Resource] 8, e80748 (2013).

138 Minnerop, M. et al. In vivo voxel-based relaxometry in amyotrophic lateral sclerosis. Journal of Neurology 256, 28-34 (2009).

139 Mioshi, E. et al. Cortical atrophy in ALS is critically associated with neuropsychiatric and cognitive changes. Neurology 80, 1117-1123 (2013).

140 Miwa, H. et al. T2-low signal intensity in the cortex in multiple system atrophy. Journal of the Neurological Sciences 211, 85-88 (2003).

141 Mori, H., Yagishita, A., Takeda, T. & Mizutani, T. Symmetric temporal abnormalities on MR imaging in amyotrophic lateral sclerosis with dementia. Ajnr: American Journal of Neuroradiology 28, 1511-1516 (2007).

142 Muller, H. P. et al. Focal alterations of the callosal area III in primary lateral sclerosis: An MRI planimetry and texture analysis. NeuroImage Clinical 26, 102223 (2020).

143 Muller, H. P. et al. Complementary image analysis of diffusion tensor imaging and 3-dimensional t1-weighted imaging: white matter analysis in amyotrophic lateral sclerosis. Journal of Neuroimaging 21, 24-33 (2011).

144 Murphy, J. M. et al. Continuum of frontal lobe impairment in amyotrophic lateral sclerosis. Archives of Neurology 64, 530-534 (2007).

145 Nasseroleslami, B. et al. Characteristic Increases in EEG Connectivity Correlate With Changes of Structural MRI in Amyotrophic Lateral Sclerosis. Cerebral Cortex 29, 27-41 (2019).

146 Ngai, S., Tang, Y. M., Du, L. & Stuckey, S. Hyperintensity of the precentral gyral subcortical white matter and hypointensity of the precentral gyrus on fluid-attenuated inversion recovery: variation with age and implications for the diagnosis of amyotrophic lateral sclerosis. Ajnr: American Journal of Neuroradiology 28, 250-254 (2007).

147 Oba, H. et al. Amyotrophic lateral sclerosis: T2 shortening in motor cortex at MR imaging. Radiology 189, 843-846 (1993).

148 Obusez, E. C. et al. 7T MR of intracranial pathology: Preliminary observations and comparisons to 3T and 1.5T. Neuroimage 168, 459-476 (2018).

149 Omer, T. et al. Neuroimaging patterns along the ALS-FTD spectrum: a multiparametric imaging study. Amyotrophic Lateral sclerosis & Frontotemporal Degeneration 18, 611-623 (2017).

150 Papma, J. M. et al. Cognition and gray and white matter characteristics of presymptomatic C9orf72 repeat expansion. Neurology 89, 1256-1264 (2017).

151 Paquin, M. E. et al. Spinal Cord Gray Matter Atrophy in Amyotrophic Lateral Sclerosis. Ajnr: American Journal of Neuroradiology 39, 184-192 (2018).

152 Peretti-Viton, P. et al. MRI of the intracranial corticospinal tracts in amyotrophic and primary lateral sclerosis. Neuroradiology 41, 744-749 (1999).

153 Piaggio, N. et al. Cord cross-sectional area at foramen magnum as a correlate of disability in amyotrophic lateral sclerosis. European Radiology Experimental 2, 13 (2018).

154 Pinkhardt, E. H., van Elst, L. T., Ludolph, A. C. & Kassubek, J. Amygdala size in amyotrophic lateral sclerosis without dementia: an in vivo study using MRI volumetry. BMC Neurology 6, 48 (2006).

155 Pinto, W. et al. O'Sullivan-McLeod syndrome: Unmasking a rare atypical motor neuron disease. Revue Neurologique 175, 81-86 (2019).

156 Placek, K. et al. UNC13A polymorphism contributes to frontotemporal disease in sporadic amyotrophic lateral sclerosis. Neurobiology of Aging 73, 190-199 (2019).

157 Prell, T. et al. Susceptibility-weighted imaging provides insight into white matter damage in amyotrophic lateral sclerosis. PLoS ONE [Electronic Resource] 10, e0131114 (2015).

158 Prell, T., Schenk, A., Witte, O. W., Grosskreutz, J. & Gunther, A. Transcranial brainstem sonography as a diagnostic tool for amyotrophic lateral sclerosis. Amyotrophic Lateral sclerosis & Frontotemporal Degeneration 15, 244-249 (2014).

159 Protogerou, G. et al. T2 FLAIR Increased Signal Intensity at the Posterior Limb of the Internal Capsule: Clinical Significance in ALS Patients. Neuroradiology Journal 24, 226-234 (2011).

160 Qin, Y. et al. Region-specific atrophy of precentral gyrus in patients with amyotrophic lateral sclerosis. Journal of Magnetic Resonance Imaging 47, 115-122 (2018).

161 Qiu, T. et al. Precentral degeneration and cerebellar compensation in amyotrophic lateral sclerosis: A multimodal MRI analysis. Human Brain Mapping 40, 3464-3474 (2019).

162 Querin, G. et al. Presymptomatic spinal cord pathology in c9orf72 mutation carriers: A longitudinal neuroimaging study. Annals of Neurology 86, 158-167 (2019).

163 Querin, G. et al. Spinal cord multi-parametric magnetic resonance imaging for survival prediction in amyotrophic lateral sclerosis. European Journal of Neurology 24, 1040-1046 (2017).

164 Raaphorst, J. et al. Prose memory impairment in amyotrophic lateral sclerosis patients is related to hippocampus volume. European Journal of Neurology 22, 547-554 (2015).

165 Radakovic, R. et al. Frontostriatal grey matter atrophy in amyotrophic lateral sclerosis A visual rating study. Dementia & Neuropsychologia 12, 388-393 (2018).

166 Rajagopalan, V. & Pioro, E. P. Distinct patterns of cortical atrophy in ALS patients with or without dementia: an MRI VBM study. Amyotrophic Lateral sclerosis & Frontotemporal Degeneration 15, 216-225 (2014).

167 Rajagopalan, V. & Pioro, E. P. Brain Parenchymal Fraction: A Relatively Simple MRI Measure to Clinically Distinguish ALS Phenotypes. BioMed Research International 2015, 693206 (2015).

168 Rajagopalan, V. & Pioro, E. P. Comparing brain structural MRI and metabolic FDG-PET changes in patients with ALS-FTD: 'the chicken or the egg?' question. Journal of Neurology, Neurosurgery & Psychiatry 86, 952-958 (2015).

169 Rajagopalan, V. & Pioro, E. P. Disparate voxel based morphometry (VBM) results between SPM and FSL softwares in ALS patients with frontotemporal dementia: which VBM results to consider? BMC Neurology 15, 32 (2015).

170 Rajagopalan, V., Yue, G. H. & Pioro, E. P. Do preprocessing algorithms and statistical models influence voxel-based morphometry (VBM) results in amyotrophic lateral sclerosis patients? A systematic comparison of popular VBM analytical methods. Journal of Magnetic Resonance Imaging 40, 662-667 (2014).

171 Ramanathan, R. S. & Rana, S. Demographics and clinical characteristics of primary lateral sclerosis: case series and a review of literature. Neurodegenerative Disease Management 8, 17-23 (2018).

172 Roeben, B., Wilke, C., Bender, B., Ziemann, U. & Synofzik, M. The motor band sign in ALS: presentations and frequencies in a consecutive series of ALS patients. Journal of the Neurological Sciences 406, 116440 (2019).

173 Sarchielli, P. et al. Magnetic resonance imaging and 1H-magnetic resonance spectroscopy in amyotrophic lateral sclerosis. Neuroradiology 43, 189-197 (2001).

174 Sasaki, S. & Iwata, M. Atypical form of amyotrophic lateral sclerosis. Journal of Neurology, Neurosurgery & Psychiatry 66, 581-585 (1999).

175 Schonecker, S. et al. Atrophy in the Thalamus But Not Cerebellum Is Specific for C9orf72 FTD and ALS Patients - An Atlas-Based Volumetric MRI Study. Frontiers in aging neuroscience 10, 45 (2018).

176 Schuster, C. et al. Cortical thinning and its relation to cognition in amyotrophic lateral sclerosis. Neurobiology of Aging 35, 240-246 (2014).

177 Schuster, C. et al. Focal thinning of the motor cortex mirrors clinical features of amyotrophic lateral sclerosis and their phenotypes: a neuroimaging study. Journal of Neurology 260, 2856-2864 (2013).

178 Schuster, C. et al. Longitudinal course of cortical thickness decline in amyotrophic lateral sclerosis. Journal of Neurology 261, 1871-1880 (2014).

179 Schweitzer, A. D. et al. Quantitative susceptibility mapping of the motor cortex in amyotrophic lateral sclerosis and primary lateral sclerosis. AJR. American Journal of Roentgenology 204, 1086-1092 (2015).

180 Senda, J. et al. Structural MRI correlates of amyotrophic lateral sclerosis progression. Journal of Neurology, Neurosurgery & Psychiatry 88, 901-907 (2017).

181 Senda, J. et al. Progressive and widespread brain damage in ALS: MRI voxel-based morphometry and diffusion tensor imaging study. Amyotrophic Lateral Sclerosis 12, 59-69 (2011).

182 Sha, S. J. et al. Frontotemporal dementia due to C9ORF72 mutations: clinical and imaging features. Neurology 79, 1002-1011 (2012).

183 Shellikeri, S. et al. Speech network regional involvement in bulbar ALS: a multimodal structural MRI study. Amyotrophic Lateral sclerosis & Frontotemporal Degeneration 20, 385-395 (2019).

184 Shen, D. et al. Brain Structural and Perfusion Signature of Amyotrophic Lateral Sclerosis With Varying Levels of Cognitive Deficit. Frontiers in neurology [electronic resource]. 9, 364 (2018).

185 Shen, D. C. et al. Monitoring Value of Multimodal Magnetic Resonance Imaging in Disease Progression of Amyotrophic Lateral Sclerosis: A Prospective Observational Study. Chinese Medical Journal 131, 2904-2909 (2018).

186 Shindo, A., Ueda, Y., Kuzuhara, S. & Kokubo, Y. Neuropsychological study of amyotrophic lateral sclerosis and parkinsonism-dementia complex in Kii peninsula, Japan. BMC Neurology 14, 151 (2014).

187 Steinbach, R. et al. Applying the D50 disease progression model to gray and white matter pathology in amyotrophic lateral sclerosis. NeuroImage Clinical 25, 102094 (2020).

188 Takeda, T., Uchihara, T., Mochizuki, Y., Mizutani, T. & Iwata, M. Memory deficits in amyotrophic lateral sclerosis patients with dementia and degeneration of the perforant pathway A clinicopathological study. Journal of the Neurological Sciences 260, 225-230 (2007).

189 Tavazzi, E. et al. Grey matter damage in progressive multiple sclerosis versus amyotrophic lateral sclerosis: a voxel-based morphometry MRI study. Neurological Sciences 36, 371-377 (2015).

190 Terada, T. et al. Frontal assessment battery and frontal atrophy in amyotrophic lateral sclerosis. Brain and Behavior 7, e00707 (2017).

191 Thivard, L. et al. Diffusion tensor imaging and voxel based morphometry study in amyotrophic lateral sclerosis: relationships with motor disability. Journal of Neurology, Neurosurgery & Psychiatry 78, 889-892 (2007).

192 Thorns, J. et al. Extent of cortical involvement in amyotrophic lateral sclerosis--an analysis based on cortical thickness. BMC Neurology 13, 148 (2013).

193 Thorpe, J. W. et al. Brain and spinal cord MRI in motor neuron disease. Journal of Neurology, Neurosurgery & Psychiatry 61, 314-317 (1996).

194 Trojsi, F. et al. Frontotemporal degeneration in amyotrophic lateral sclerosis (ALS): a longitudinal MRI one-year study. Cns Spectrums, 1-10 (2020).

195 Turner, M. R. et al. Volumetric cortical loss in sporadic and familial amyotrophic lateral sclerosis. Amyotrophic Lateral Sclerosis 8, 343-347 (2007).

196 Udaka, F. et al. MRI and SPECT findings in amyotrophic lateral sclerosis. Demonstration of upper motor neurone involvement by clinical neuroimaging. Neuroradiology 34, 389-393 (1992).

197 Van Mossevelde, S. et al. Clinical features of TBK1 carriers compared with C9orf72, GRN and non-mutation carriers in a Belgian cohort. Brain 139, 452-467 (2016).

198 Vazquez-Costa, J. F. et al. The width of the third ventricle associates with cognition and behaviour in motor neuron disease. Acta Neurologica Scandinavica 139, 118-127 (2019).

199 Vazquez-Costa, J. F. et al. Brain signal intensity changes as biomarkers in amyotrophic lateral sclerosis. Acta Neurologica Scandinavica 137, 262-271 (2018).

200 Verstraete, E. et al. No evidence of microbleeds in ALS patients at 7 Tesla MRI. Amyotrophic Lateral Sclerosis 11, 555-557 (2010).

201 Verstraete, E. et al. Structural MRI reveals cortical thinning in amyotrophic lateral sclerosis. Journal of Neurology, Neurosurgery & Psychiatry 83, 383-388 (2012).

202 Vibha, D. et al. Clinical profile of Monomelic Amyotrophy (MMA) and role of persistent viral infection. Journal of the Neurological Sciences 359, 4-7 (2015).

203 Vinceti, G. et al. Primary progressive aphasia and the FTD-MND spectrum disorders: clinical, pathological, and neuroimaging correlates. Amyotrophic Lateral sclerosis & Frontotemporal Degeneration 20, 146-158 (2019).

204 Walhout, R. et al. Cortical thickness in ALS: towards a marker for upper motor neuron involvement. Journal of Neurology, Neurosurgery & Psychiatry 86, 288-294 (2015).

205 Waragai, M. MRI and clinical features in amyotrophic lateral sclerosis. Neuroradiology 39, 847-851 (1997).

206 Waragai, M., Shinotoh, H., Hayashi, M. & Hattori, T. High signal intensity on T1 weighted MRI of the anterolateral column of the spinal cord in amyotrophic lateral sclerosis. Journal of Neurology, Neurosurgery & Psychiatry 62, 88-91 (1997).

207 Welton, T. et al. Diffusion kurtosis and quantitative susceptibility mapping MRI are sensitive to structural abnormalities in amyotrophic lateral sclerosis. NeuroImage Clinical 24, 101953 (2019).

208 Westeneng, H. J. et al. Subcortical structures in amyotrophic lateral sclerosis. Neurobiology of Aging 36, 1075-1082 (2015).

209 Westeneng, H. J. et al. Widespread structural brain involvement in ALS is not limited to the C9orf72 repeat expansion. Journal of Neurology, Neurosurgery & Psychiatry 87, 1354-1360 (2016).

210 Whitwell, J. L., Jack, C. R., Jr., Senjem, M. L. & Josephs, K. A. Patterns of atrophy in pathologically confirmed FTLD with and without motor neuron degeneration. Neurology 66, 102-104 (2006).

211 Wirth, A. M. et al. Value of fluid-attenuated inversion recovery MRI data analyzed by the lesion segmentation toolbox in amyotrophic lateral sclerosis. Journal of Magnetic Resonance Imaging 50, 552-559 (2019).

212 Wu, R. H. & Bruening, R. Comparison of diffusion-weighted MR imaging and T2-weighted MR imaging in patients with amyotrophic lateral sclerosis. Neuroradiology Journal 19, 705-710 (2006).

213 Yagishita, A., Nakano, I., Oda, M. & Hirano, A. Location of the corticospinal tract in the internal capsule at MR imaging. Radiology 191, 455-460 (1994).

214 York, C. et al. Action verb comprehension in amyotrophic lateral sclerosis and Parkinson's disease. Journal of Neurology 261, 1073-1079 (2014).

215 Yu, J. et al. Increased iron level in motor cortex of amyotrophic lateral sclerosis patients: an in vivo MR study. Amyotrophic Lateral sclerosis & Frontotemporal Degeneration 15, 357-361 (2014).

216 Yunusova, Y. et al. Frontal Anatomical Correlates of Cognitive and Speech Motor Deficits in Amyotrophic Lateral Sclerosis. Behavioural Neurology 2019, 9518309 (2019).

217 Zhang, J. et al. Regional alterations in cortical thickness and white matter integrity in amyotrophic lateral sclerosis. Journal of Neurology 261, 412-421 (2014).

218 Zhang, L. et al. The diagnostic utility of FLAIR imaging in clinically verified amyotrophic lateral sclerosis. Journal of Magnetic Resonance Imaging 17, 521-527 (2003).

219 Zhang, Q. et al. Side of limb-onset predicts laterality of gray matter loss in amyotrophic lateral sclerosis. BioMed Research International 2014, 473250 (2014).

220 Zhang, Y. et al. Occipital cortical gyrification reductions associate with decreased functional connectivity in amyotrophic lateral sclerosis. Brain Imaging & Behavior 11, 1-7 (2017).

221 Zhang, Y. et al. Abnormal topological organization of structural covariance networks in amyotrophic lateral sclerosis. NeuroImage Clinical 21, 101619 (2019).

222 Zhu, W. et al. ALFF Value in Right Parahippocampal Gyrus Acts as a Potential Marker Monitoring Amyotrophic Lateral Sclerosis Progression: a Neuropsychological, Voxel-Based Morphometry, and Resting-State Functional MRI Study. Journal of Molecular Neuroscience 57, 106-113 (2015).

**References**

1 Wells GA, Tugwell P, O’Connell D et al (2015) The Newcastle-Ottawa Scale (NOS) for assessing the quality of nonrandomized studies in meta-analyses.
